# Human activity and mobility data reveal disparities in exposure risk reduction indicators among socially vulnerable populations during COVID-19 for five U.S. metropolitan cities

**DOI:** 10.1038/s41598-022-18857-7

**Published:** 2022-09-22

**Authors:** Natalie Coleman, Xinyu Gao, Jared DeLeon, Ali Mostafavi

**Affiliations:** 1 Zachry Department of Civil and Environmental Engineering, Urban Resilience.AI Lab, Texas A&M University, College Station, USA; 2 Urban Resilience.AI Lab, Texas A&M University, College Station, USA

**Keywords:** Computational science, Mathematics and computing

## Abstract

Non-pharmacologic interventions (NPIs) promote protective actions to lessen exposure risk to COVID-19 by reducing mobility patterns. However, there is a limited understanding of the underlying mechanisms associated with reducing mobility patterns especially for socially vulnerable populations. The research examines two datasets at a granular scale for five urban locations. Through exploratory analysis of networks, statistics, and spatial clustering, the research extensively investigates the exposure risk reduction after the implementation of NPIs to socially vulnerable populations, specifically lower income and non-white populations. The mobility dataset tracks population movement across ZIP codes for an origin–destination (O–D) network analysis. The population activity dataset uses the visits from census block groups (cbg) to points-of-interest (POIs) for network analysis of population-facilities interactions. The mobility dataset originates from a collaboration with StreetLight Data, a company focusing on transportation analytics, whereas the population activity dataset originates from a collaboration with SafeGraph, a company focusing on POI data. Both datasets indicated that low-income and non-white populations faced higher exposure risk. These findings can assist emergency planners and public health officials in comprehending how different populations are able to implement protective actions and it can inform more equitable and data-driven NPI policies for future epidemics.

## Introduction

Since the first COVID-19 case in the United States (US) was reported on January 21, 2020 in Snohomish County, Washington^1^, the SARS-CoV-2 virus has rapidly spread across the country. As of January 23, 2022, there have been more than 70.7 million confirmed cases and approximately 866,000 deaths are attributed to the disease in the United States^2^. To decrease the contact and transmission rate of COVID-19, many states implemented state or local level stay-at-home policies as well as the closure of non-essential services starting in mid-March 2020. Non-pharmacologic interventions (NPIs), which encourage protective actions via social distancing and sheltering-in-place, are effective measures to slow down the spread of COVID-19^3–5^.

Life and daily movement patterns were greatly altered by this pandemic. According to guidelines associated with NPIs, places regarded as non-essential such as schools, gyms, bars, and other commercial complexes, were temporally closed, and mass gatherings and celebratory events were cancelled or postponed^6^. People also tried to curtail their daily essential activities (e.g., refueling cars, purchasing goods) to decrease the risk of infection^7–9^. Such changes in movement could be a proxy measurement for the protective actions taken to reduce exposure risk^10^. To understand the influence and effectiveness of such social distancing practices, many studies have analyzed real-time movement data at the country level^11,12^, county level^13–16^, and city level^17–19^. These studies show that the implementation of NPIs significantly reduced human activities, and by extension, they also reduced possible transmission of the virus. Mobility data and population activity has shown to be an advantageous data source in collecting pattern movements and relating those to potential COVID-19 exposure^20^. However, it is difficult to ignore the varying effectiveness that COVID-19 and NPIs could have on different populations. For example, research on co-location reduction^10^, heterogenous features^21^, and urban hotspots^19,22^ shows evidence of mobility segregation patterns. However, studies of highly-aggregated human movement and mobility data may have missed critical disparity among different demographic groups, particularly those that are classified as socially vulnerable populations^23^.

Historically, socially vulnerable populations have been connected to various societal issues such as disaster recovery, educational resources, and health inequalities^24–29^. According to Centers for Disease Control (CDC), social vulnerability refers to “residents with socioeconomic and demographic factors that affect the resilience of communities”^30^. This established social vulnerability framework includes households of lower income and of racial-ethnic minority status, who are typically vulnerable for their lack of resources and exclusion from governmental planning. Disaster literature supports that socially vulnerable populations are disproportionately impacted by disasters^30^. In the ever-evolving research literature of the COVID-19 pandemic, earlier studies and reports have also captured the disparate impacts associated with different socially vulnerable groups at both the community and individual level. For example, Benitez and Yelowitz^31^ found that predominately Black and Hispanic neighborhoods had higher COVID-19 cases per capita and higher observed fatalities. Similarly, Abedi et al.^32^ concluded that counties with more diverse demographics, such as those with larger population, larger percentage of minority households, lower educational attainment, lower income, or higher disability rates were at a higher risk of COVID-19 infection. In particular, African Americans were more vulnerable to COVID-19 than other racial-ethnic groups. At the county level, Ossimetha^33^ found that counties with socioeconomic disadvantages and less reduced mobility had greater growth in COVID-19 cases and deaths. Similarly, Li et al.^21^ showed that demographic features such as population density, gross domestic product, and minority status, were of high-importance features in case predictions. Borgonovi and Andrieu^34^ found that counties whose residents present pre-existing medical conditions and low levels of community social capital were more susceptible to experiencing increased rate of infection of COVID-19, even suggesting that social distancing practices were related to behavioral changes in mobility. These studies emphasized the exposure and inherent risk disparity of socially vulnerable groups; however, only a limited number of studies have thoroughly investigated the extent to which exposure risk reduction conferred by NPIs varied across different populations. Evaluating the exposure risk reduction indicators through the exploratory analysis of granular human mobility and population activity datasets may hold the key to understanding exposure disparities among low-income and racial-ethnic minority populations.

Part of the limitation in studying the effects of NPIs is that current published research focuses on human movement and COVID-19 outcomes at highly aggregated levels (i.e., state- or county level). Coarse-level disparity analysis may ignore an important part of the variation as residential segregation by socially vulnerable populations can be significant in finer spatial scales. Studies of social vulnerability warn that coarse-scale analysis may fail to detect critical instances of disparities, such as those prominent in inner cities^35^. In fact, finer-scale analysis may yield different results compared to coarser-scale analysis as observed by the law of averages^23,36^. Studies focusing on fine-scale analysis of disparities in movements and activity reduction of different populations in the context of COVID-19 are rather limited. After analyzing census block groups (cbgs), Fan et al.^37^ suggested that localized area-specific policies could be effective measures of containing infections. In addition, Benitez and Yelowitz^31^ conducted racial-ethnic disparity analysis in COVID-19 cases per capita at the ZIP-code level for six cities, and the findings support that Black and Hispanic populations are correlated with higher rates of COVID-19 cases. The study acknowledges a knowledge gap related to the underlying mechanisms leading to such risk disparities and emphasizes a need to understand such disparities at a granular level. Even among the limited existing studies, the majority have analyzed single mobility and/or population activity datasets. This limits the ability to holistically understand different indicators of exposure risk to the COVID-19 virus. Since each dataset might have limitations regarding aspects of mobility movements and population activities captured, it is essential to conduct studies with different datasets with intentionality to dissect, interpret, and integrate multiple indicators of exposure risk.

Thus, this research study addresses the knowledge gap by examining the disparities associated with the protective actions to reduce the risk of transmission and by differentiating the mobility and population activity patterns of socially vulnerable groups. Through the exploratory analysis of networks, statistics, and spatial clusters, this study measures the extent of exposure risk reduction of different income groups and different racial-ethnic groups. Using three indicators of exposure risk, the study incorporates two datasets at a granular level to capture insights which otherwise would have been overrun by coarser scale analysis. The first indicator captures the number of trips based on a ZIP code-to-ZIP code origin–destination (O–D) network analysis. This indicator provides insights regarding cross-ZIP code transmission risk of the virus by measuring the number of trips to nodes in the network, which corresponds to the center point of each ZIP code. The greater the inflow measure of the number of trips to nodes within ZIP codes, referred in this paper as the in-degree flow of a ZIP code, the higher the exposure risk of residents in that ZIP code to virus transmission from other ZIP codes. The second indicator examines the exposure risk of population activity fluctuations which refers to contact at POIs. The third indicator captures the exposure risk from the points-of-interest to census block groups (POI-CBG) network which refers to previous transmission at POIs to home cbgs. These three indicators provide distinct measures as proxies for evaluating exposure risk reductions afforded by NPIs and enable us to examine the disparities among vulnerable populations. The spatiotemporal context of the study comprises of five US locations: (1) Cook County (Chicago), Illinois, (2) Harris County (Houston), Texas, (3) New York City, New York, (4) Los Angeles County (Los Angeles), California, (5) King County (Seattle), Washington, recorded between January 1, 2020 through July 31, 2020.

## Methods

### Description of mobility data and population activity

The research uses two datasets for mobility patterns and population activity. First, the research had a partnership with the StreetLight Data Company to obtain mobility data. StreetLight harnesses smartphones as sensors to measure vehicle, transit, bike, and foot traffic that show travel patterns in selected geographic areas^38^. Several case studies in North America have used StreetLight mobility data for transportation analytics^39^. Per month, the company processes and aggregates approximately 40 billion anonymized records that includes more than 5 million miles of roadway, sidewalk, and bike lanes^38^. On average, it captures 65 M devices in the United States and Canada which samples approximately 23% of the population and 18% of trips^40,41^. StreetLight has a distribution of the demographic characteristics of each trip using the most updated American Community Survey (ACS) data. Demographic data has been shown to be representative demographic information of the selected geographic areas^42^. This feature of the data divided income into six groups of median income and six groups of racial-ethnic populations (Supplementary Information B). The research study aggregated the mobility data to a ZIP code-to ZIP-code O–D network to analyze the number of inflow trips across ZIP-codes. After filtering the StreetLight data, 83,460,324 total datapoints were used in the final analysis which consisted of the following: Chicago had 11,638,220 points; Houston had 29,789,279 points; New York City had 15,485,617 points; Los Angeles had 22,712,811 points; and Seattle had 3,834,397. In this case, unique points refers to the O–D trip paths in the mobility network at a weekly scale.

Second, the research had a partnership with the SafeGraph Company to obtain population activity data. SafeGraph provides the most accurate points-of-interest (POIs) and store location geofences for the US. It contains more than 3.6 MM commercial points and 45 MM mobile devices, which samples approximately 10% of US devices. Aggregated demographics of the sampled devices accurately represents the demographics of the selected geographic areas^43,44^. POIs include physical locations in the community such as restaurants, retail stores, and grocery stores. The boundary of each POI is made up of polygons or single points depending on the data resolution. The dataset is connected to the physical locations of POIs to the North American Industry Classification System (NAICS) which categorize business and industry codes^45^. SafeGraph uses a thorough methodology of DBSCAN clustering and machine learning to detect visits to POIs, where visits are registered at a threshold of four minutes at a POI^46^. Population activity dataset was manually merged with downloaded 2019 ACS survey data at the cbg level. Using the cbg identification, cbgs of POIs and the home cbgs of residents were connected to their median income levels and the percentage of white-only and non-white households which total to 100%^47^. The median income were divided into six percentile groups based on the selected geographic area. A sensitivity analysis, found in Supplementary Information D, was performed on the number of bins. It showed no difference in the rankings of income groups. Using this dataset, a network was created to examine the number of visits to POIs as well as the number of visits from home cbg. After filtering SafeGraph data, the researchers used a total of 354,034 total data points which consisted of the following: Chicago had 55,292 points; Houston had 52,776 points; New York City had 72,304 points; Los Angeles had 146,037 points; and Seattle had 27,625 points. In this case, unique points refers to the number of POIs in the population activity data at a weekly scale.

Both the mobility dataset and the population activity dataset covered five US cities: (1) Cook County (Chicago), Illinois, (2) Harris County (Houston), Texas, (3) New York City (comprising five counties) (4) Los Angeles County (Los Angeles), California, (5) King County (Seattle), Washington. The first four locations include the four most populated cities in the US; Seattle was also included because the city was first recorded instance of an individual diagnosed with the COVID-19 virus in the US. The selected locations are also widespread across regions of the US to contrast differences in impact. The analysis time period was January 1, 2020 through July 31, 2020, which is generally considered to be the first wave of the pandemic. The majority of NPIs such as shelter-in-place orders took place during mid-March and were mostly released by late May and early June 2020 (Supplementary Information A). To normalize the data, a baseline level for mobility movement and average POI unique visits was established between January 13, 2020 and February 4, 2020. The baseline was selected after the early weeks of the year to give a more stable comparison that was not biased towards the heavy tourism and movement of the typical holidays. This baseline period has also been previously used by published literature studying the mobility changes during the COVID-19 pandemic^48,49^.

### Description of exposure risks

**Figure 1:**
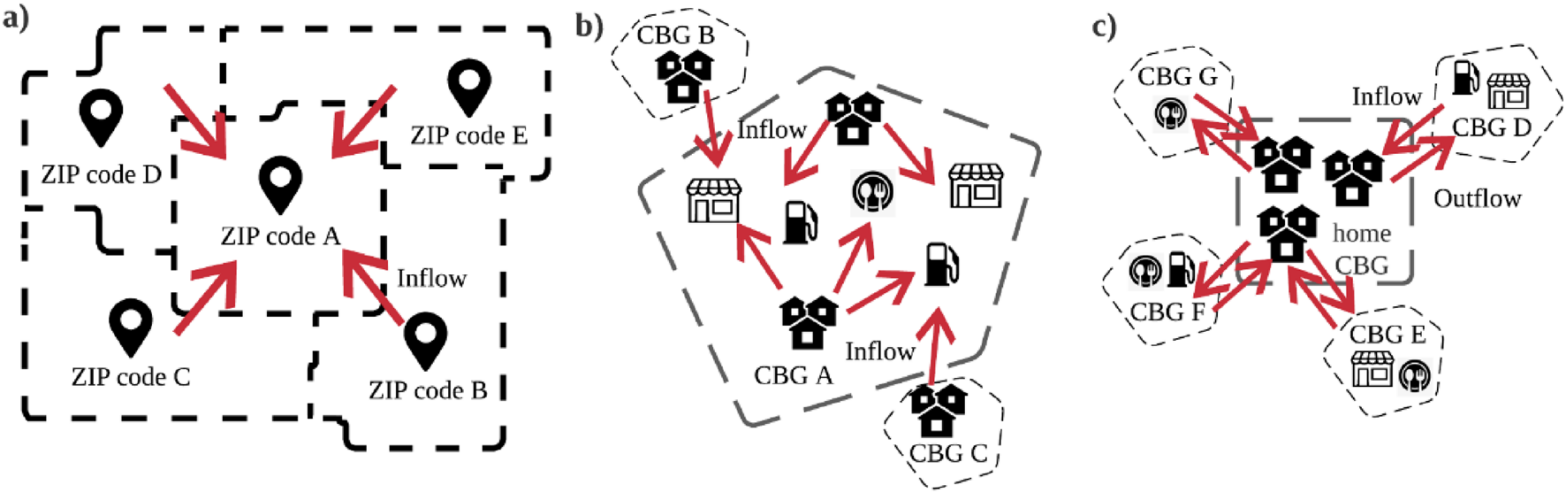
Mobility patterns and population activity yield exposure risk indicators. Exposure risk indicators are measured through (**a**) inflow measures from ZIP codes B, C, D, E to ZIP code A, (**b**) percent change of visits to POIs in CBG A with from CBG B and CBG C, (**c**) previous transmission at POIs in CBG D, E, F, G to home CBG.

The first exposure risk indicator measures the increased transmission across ZIP codes from trips. It is represented as the inflow measure of trips, or in-degree values, of ZIP codes. Greater in-degree of a ZIP code signals a higher exposure risk of residents in that ZIP code due to the possibility of virus transmission from other ZIP codes. In Fig. 1a, the research would examine the first exposure risk of ZIP code A from the surrounding ZIP codes. The second exposure risk indicator is the population activity fluctuations which refers to the possibility of contact at POIs. It is measured as the number of visits to POIs within different cbgs. Greater percent change of visits being received by POIs within a particular cbg signal a higher exposure risk to the residents residing in that cbg. In Fig. 1b, the research would examine the second exposure risk of the POIs in CBG A from the surrounding cbgs. The third exposure risk is the POI-CBG network which refers to the transmission to home cbgs due to previous contact in outside POIs. It is measured as the number of visits to POIs from different home cbgs. Greater percent change of visits to outside POIs coming from the residents of a home cbg signal a higher exposure risk to the residents residing in the same home cbg. In Fig. 1c, the research would examine the third exposure risk of the home CBG from the surrounding CBGs.

#### ZIP code-level mobility network

The mobility data describes the hourly number of trips for each pair of O–D links across all ZIP-codes. The O–D network is constructed where the centroid points of all ZIP-code areas are considered as nodes. At time $t$, if there exist trips from ZIP code $i$ to ZIP code $j$, a link between these two points will be constructed, and the number of trips, ${N}_{i,j}(t)$, is assigned as the weight of this link. For each node $i$, the weighted degree ${D}_{i}$ can be calculated by summing all the weights of links connected to node $i$ using Eq. (1). This allowed us to calculate the movement inflows referred to as in-degree values.

1 $${D}_{i}(t)={\sum }_{j}{N}_{i,j}(t)$$

where, *D*_*i*_*(t)* is the in-degree/out-degree, and *N*_*i,j*_* (t)* is the number of trips of starting node (*i*) and ending node (*j*).

#### Population activity fluctuations

The percent change of visits to POIs from the baseline was calculated as shown in Eq. (2)

2 $${\mathrm{PC}}_{\mathrm{i}}=\frac{{\mathrm{Visits}}_{\mathrm{i}}-\mathrm{Baseline}}{\mathrm{Baseline}}*100\%$$

where, PC_i_ is the percent change of visits, Visits_i_ is the number of visits at week i, and Baseline is average visits between January 13, 2020 and February 3, 2020.

#### POI-CBG network of home cbgs

When possible, SafeGraph provides the number of visits from home cbgs. Since the scope of this exposure risk was to measure the home cbgs in the selected county; captured home cbgs from a different county were not used in the analysis. This kept the exposure risk indicator as a measure within the residents of the county. A network analysis is created from the link between home cbg to POI. Equation (2) calculated the percent change of mobility.

### Exploratory analysis of exposure risks

**Figure 2:**
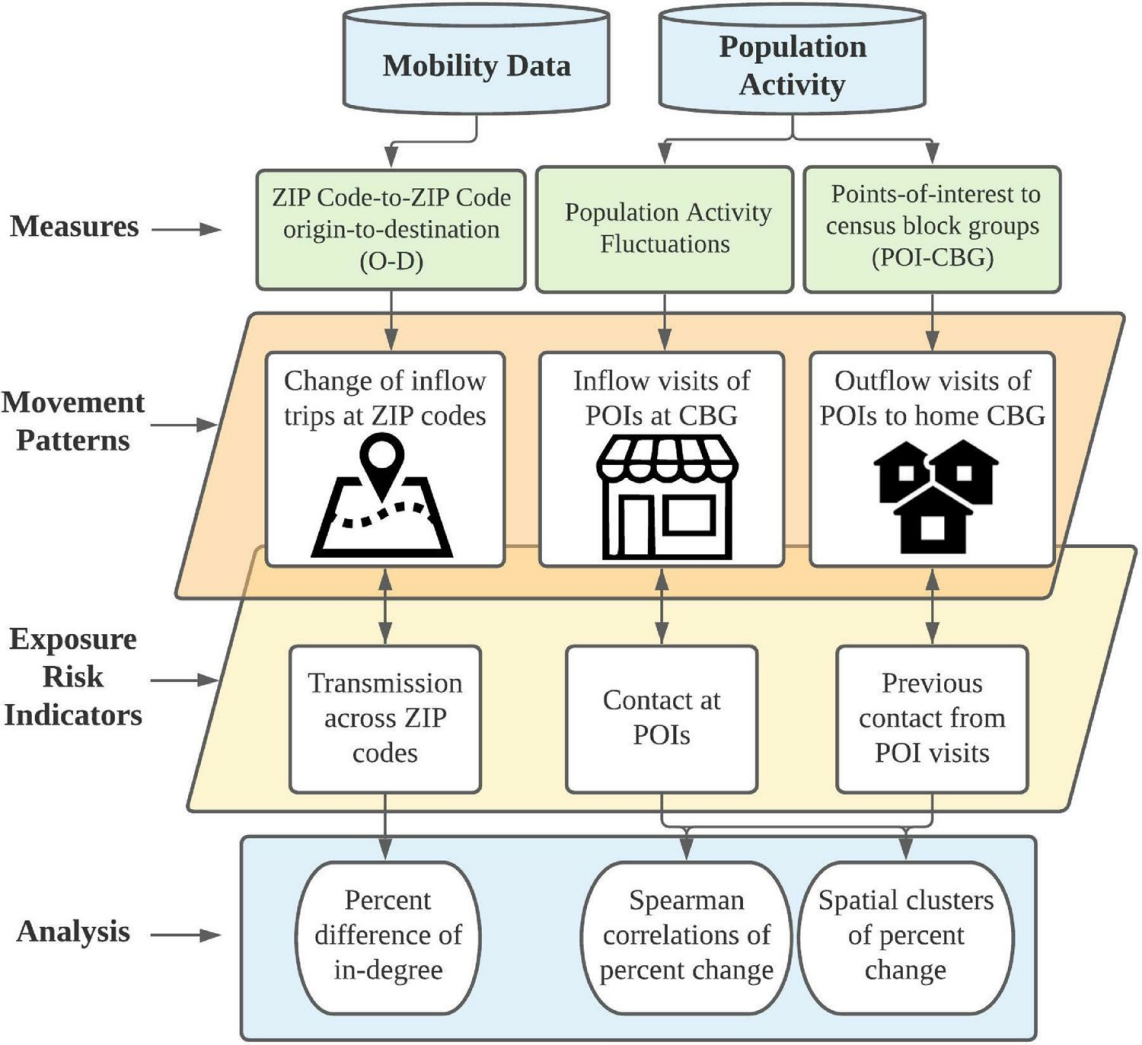
Method process of mobility data population activity from data collection to data analysis.

Figure 2 summarizes the exploratory analysis of the exposure risks. First, in-degree values were analyzed on a weekly basis for the income groups and racial-ethnic groups. Trips were normalized based on the volume of trips divided by the baseline number of trips for each social group to account for uneven distributions. An example of the O–D Network, which shows the percent change from inflow measures and outflow measures from the established baseline, can be found in Supplementary Information C. Second, spearman correlations measure the statistical significance of the median income levels and percentage of non-white populations to changes in human mobility from the baseline levels regarding the population activity fluctuations and POI-CBG network. It examines whether there is statistically significant (p < 0.05) difference between values of different income levels and different percentage of non-white populations in their ability to reduce movement compared to baseline levels. These correlations and statistical significance were calculated for each week since the relationship between demographics and mobility may vary over time. The research wanted to determine whether the potential disparity in mobility was consistent or specific to a time period. Regarding the population activity fluctuations, negative correlations indicated that lower-income cbgs or cbgs of greater percentage of non-white populations had lower percent change from the baseline and more exposure risk comparatively. Regarding the POI-CBG network, negative correlations indicate that that residents from lower-income cbgs or those with a greater percentage of non-white populations were less able to reduce their exposure risk and were traveling to more POIs. Third, bivariate Moran’s I statistic was calculated to examine the spatial autocorrelation of the population activity fluctuations and POI-CBG network. The global Moran’s I statistic was first calculated between the percentage change to the baseline at population activity fluctuations and the POI-CBG network which is represented by the correlation coefficient to determine the potential of spatial clustering. The correlation coefficient is measured from a scale of − 1 to 1, where a correlation coefficient further away from 0 represents that there is less randomness in the spatial clustering. Statistical significance was based on 999 permutations as computed through the GeoDa software. Next, the local Moran’s I bivariate revealed specific clusters of cbgs that were statistically significant (p < 0.05) shown through the LISA clusters. This revealed areas of high vulnerability and low vulnerability which is otherwise not shown through correlations. As shown in Eq. (3), clusters are generated from two variables: the percent change to the baseline and median income level or percentage of non-white population. Clusters can either be high–high (H–H), high–low (H–L), low–high (L–H), or low–low (L–L). H–H clusters represent areas of high socially vulnerable populations (low income or non-white population) and high exposure risk (less percent change from baseline). The L–L clusters represent areas of low socially vulnerable populations (high-income or white-only) and low exposure risk (greater percent change from baseline). Please see more information in Supplementary E.

3 $${I}_{t}=\frac{R*{\sum }_{i=1}^{R}{\sum }_{j=1}^{R}{{w}_{ij}x}_{i*}{x}_{j}}{{R}_{b}\sum_{i=1}^{R}{x}_{i}^{2}}$$

where, *I*_*t*_ is the Moran’s I statistic, *R* represents the number of regions (cbgs) in the dataset; *w* is the weight of the socially vulnerable population (income group or racial-ethnic group); *x* is the percent change at cbg (*i*) and cbg (*j).*

## Results

### First exposure risk indicator

**Figure 3:**
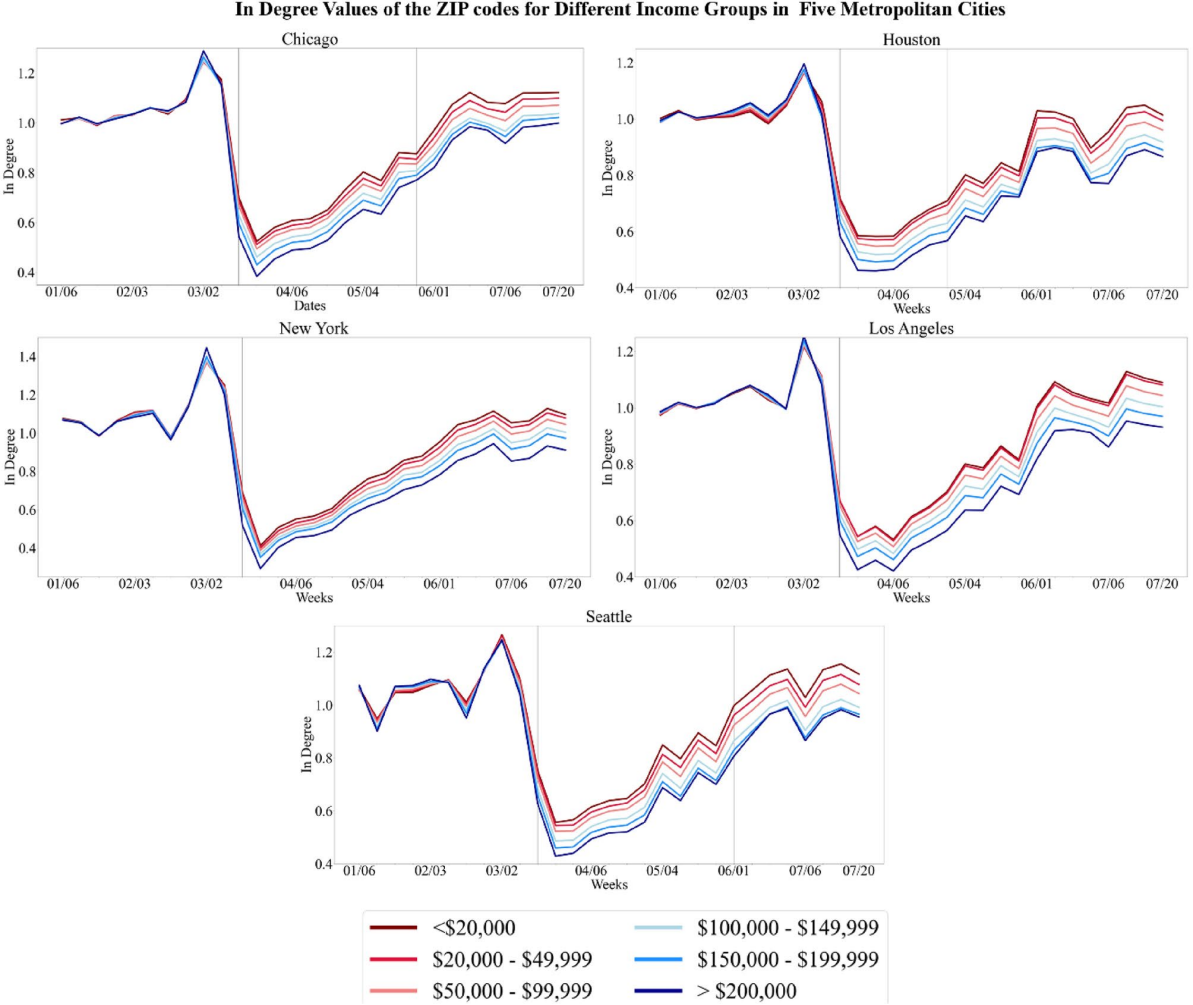

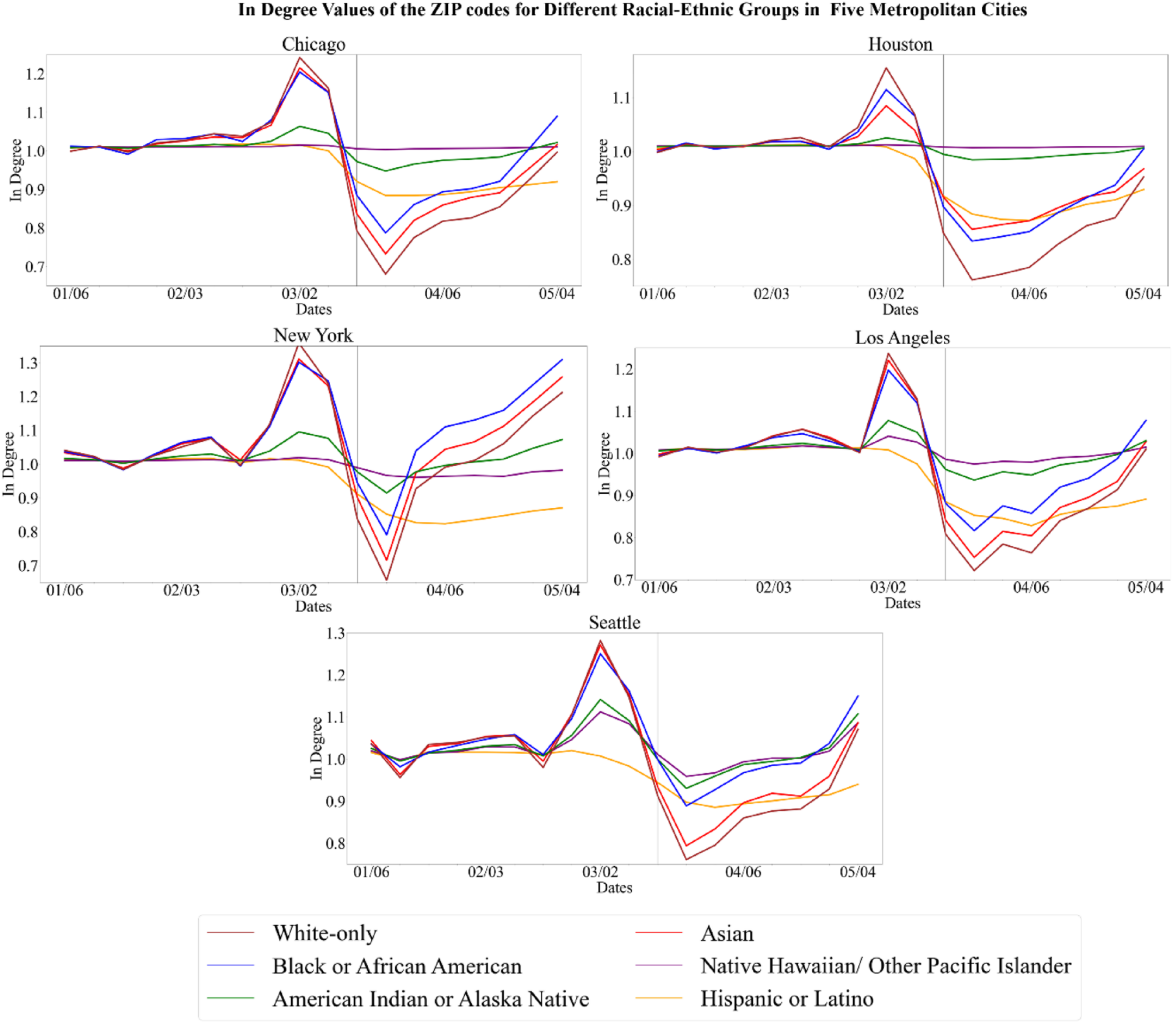
Variation of inflow of trips in ZIP codes for different income groups (top) and variation of inflow of trips in ZIP codes for different racial-ethnic groups (bottom). In-degree values, or inflow measure of trips, are normalized for each group to account for uneven distribution. The vertical black line is the week that NPI shelter-in-place was implemented in each county, and, if applicable, the vertical gray line is the week that NPI shelter-in-place was lifted. The percentage of each racial-ethnic group, except Hispanic which overlaps, totals to 100% population.

The first exposure risk indicator accounts for the transmission across ZIP codes based on O–D mobility network (Fig. 3). Following the implementation of NPIs, there is a notable divergence of in-degree values, or the inflow measure of trips, among the different groups. In-degree values dropped after March 16th but returned to baseline values for all urban cities and for all demographic groups by the end of July. However, the drop of in-degree values was comparatively less for ZIP codes with lower-income residents which indicates a higher exposure risk for lower income populations. Table 1 displays an example of comparing the percent difference of different income groups to the lowest income group. After the implementation of NPIs, there was a greater percent difference between lower and higher income groups. In the week of March 30th to April 5th, the < $20,000 income group had a 13–18% difference to the $150–$200 k group and a 20–22% to the > $200,000 income group for the five urban locations.

**Table 1:** Percent difference of in degree values for March 30th to April 5th.

Percent difference to < $20,000					
	$20,000–$49,999	$50,000–$99,999	$100,000–$150,000	$150,000–$200,000	> $200,000
Chicago	2.69	5.86	11.14	15.66	21.85
Houston	2.19	6.01	11.03	15.40	20.63
Los Angeles	0.47	4.26	8.91	13.21	20.82
New York	3.57	6.96	10.42	13.53	20.55
Seattle	3.51	7.35	13.56	18.06	22.31

Percent difference to white					
	Black or African American	American Indian or Alaska Native	Asian	Native Hawaiian/ Other Pacific Islander	Hispanic or Latino
Chicago	− 11.01	− 24.43	− 5.71	− 29.59	− 14.04
Houston	− 9.05	− 27.66	− 11.93	− 30.50	− 13.24
Los Angeles	− 11.60	− 21.90	− 3.88	− 25.07	− 7.82
New York	− 12.09	− 5.41	− 5.01	− 3.59	10.83
Seattle	− 16.57	− 20.65	− 4.84	− 21.62	− 11.32

The percent difference of the in-degree values were calculated between the different income groups and racial-ethnic groups. In-degree values indicates the inflow measure of trips to the ZIP codes. This table shows an example of the different income groups comparing to the lowest income group (< $20,000) and an example to the different racial-ethnic groups to the white population. All the values can be found in Supplementary Information F.

Regarding racial-ethnic groups, there is a notable divergence of in-degree values during the implementation of NPIs. Across five urban locations (Fig. 3), White-only populations had the greatest drop of in-degree values, meaning these populations had the lowest comparative exposure risk. Native Hawaiian/Other Pacific Islander and American Indian or Alaska Native populations showed virtually no change in their in-degree values, and thus, the highest comparative exposure risk. By comparison, Black or African American and Asian populations had a lower drop of in-degree values than White-only populations, but a higher drop of in-degree values than Native Hawaiian/Other Pacific Islander and American Indian or Alaska Native populations. Results for Hispanic populations were mixed. The group initially had the third lowest decrease of in-degree values. The ranking of this initial drop stayed consistent for a certain timeframe but the group concluded with the greatest drop of inflow measures at the end of the analysis period. This indicates that although the initial exposure risk level of the Hispanic population decreased over time when compared to the other populations. Table 1 shows an example of the percent difference tabulation by comparing white population to other racial-ethnic groups. The percent differences support the visualizations of the racial-ethnic groups.

### Second exposure risk indicator

**Figure 4:**
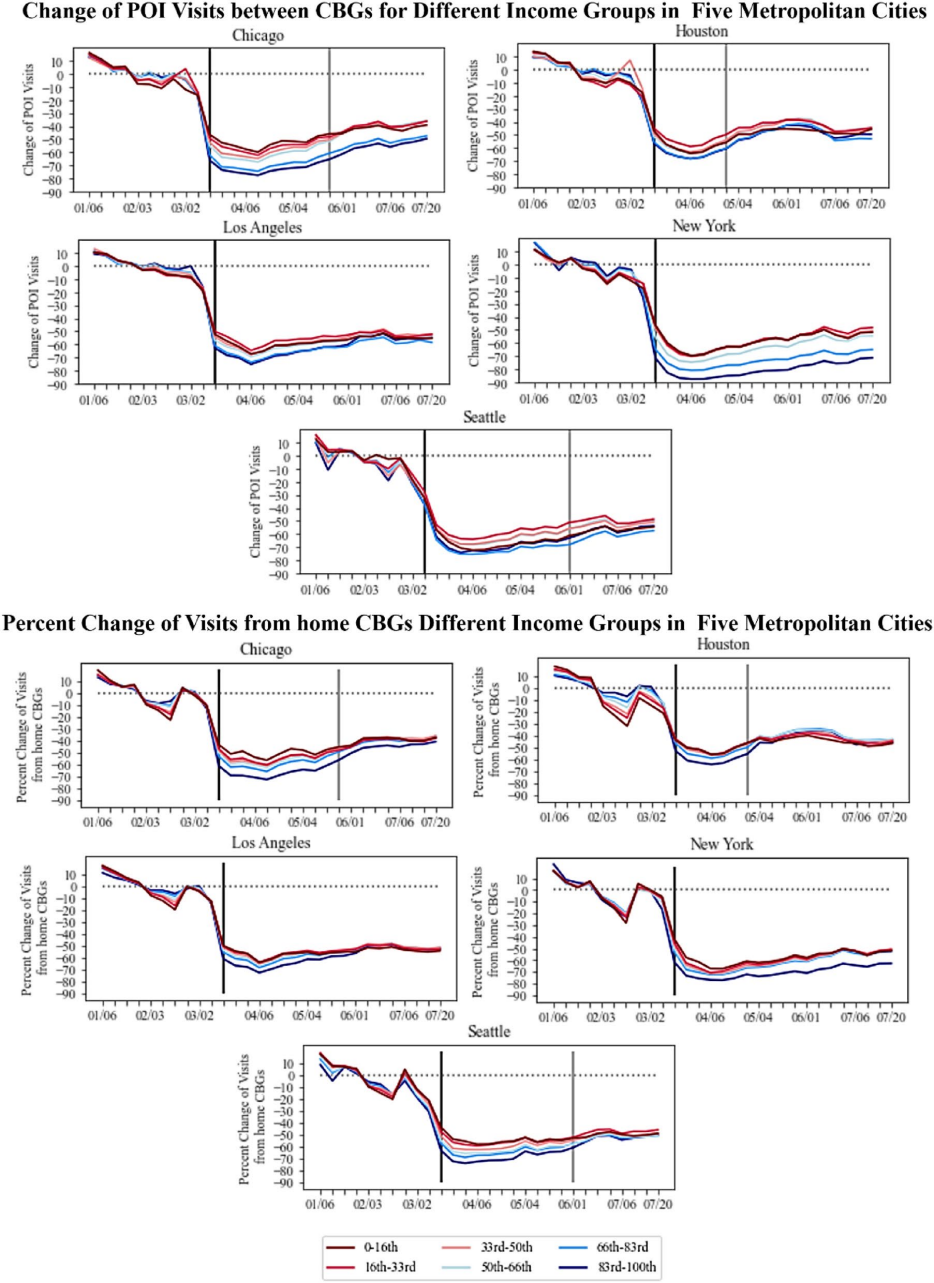
Percent change in POI visits between cbgs for different income groups (top) and POI percent change of total visits of POIs from home cbgs for different income groups (bottom). The percent change values of points-of-interest (POIs) are normalized for the median income levels and non-white percentage populations. The vertical black line is the week that NPI shelter-in-place was implemented in each county, and, if applicable, the vertical gray line is the week that NPI shelter-in-place was lifted. The bright red line represents the bottom 16th percentile of income (low income) while the bright blue line represents the top 83rd percentile of income (high income).

The second exposure risk indicator accounts for the percentage change of population activity fluctuations based on contact at POIs (Fig. 4). Greater percentage change of POI visits indicates higher comparative exposure risk. Generally, POI percent change did not return to baseline levels and stayed at − 40% from the baseline, which differs from the mobility dataset. Though mobility within a community, or number of trips, may have returned to a steady state, this does not mean that people are physically entering the businesses and organizations as some offered curbside pickup, delivery, and virtual services. Following the implementation of NPIs, lower income cbgs had a less drop in population activity fluctuations when compared to higher income cbgs. This result indicates that lower-income cbgs had higher comparative exposure risk.

**Figure 5:**
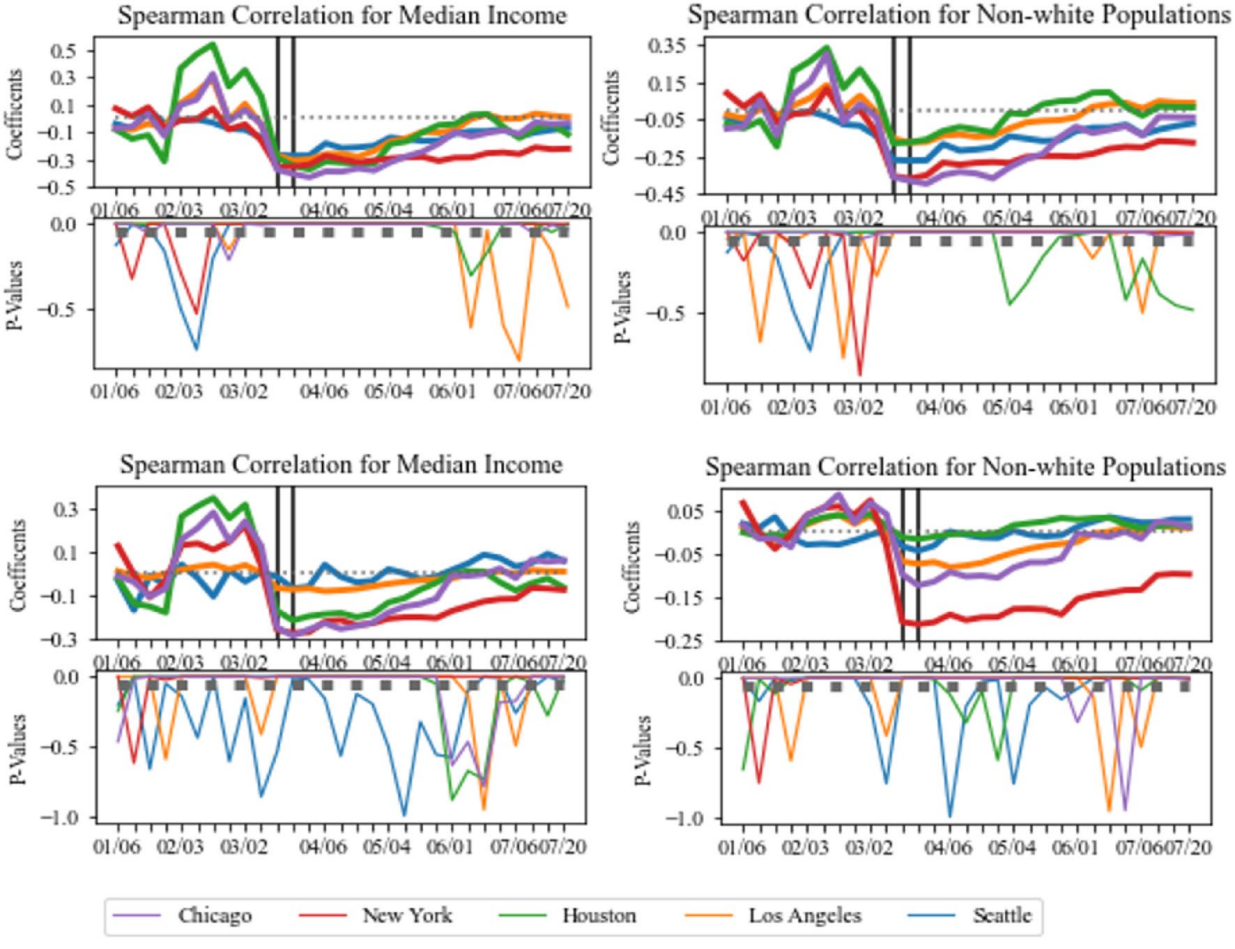
(Top) Spearman correlations of population activity fluctuations against median income levels and nonwhite populations of POI cbgs. (Bottom) Spearman correlations of POI-CBG network against median income levels and nonwhite populations of home cbgs. The two vertical black lines indicate the weeks when NPIs implemented since this varied for the five urban locations. p-values are statistically significant at p < 0.05.

Correlations were conducted between the median income levels and the percentage of nonwhite populations (Fig. 5) to determine the potential disparity in the ability of lower income and nonwhite populations to reduce their mobility over time. After the implementation of NPIs, the correlations for the median income levels flipped from positive to negative which indicates a shift in population activity for all five urban locations. This statistical significance over time did not remain consistent for all cities as the time periods varied. Between March 16th and June 1st, Chicago, New York City, and Houston had correlation values between − 0.15 and − 0.30 at statistically significant p-values. After June 1st, only New York City retained statistically significant negative correlations to the end of the analysis period. This indicates that lower income cbgs had higher comparative exposure risk in population activity. After the implementation of NPIs, Chicago and New York City had correlation values between − 0.10 and − 0.25 at statistically significant p-values. In those cities, cbgs with greater percentage of nonwhite populations were less able to reduce their mobility and thus had higher comparative exposure risk.

### Third exposure risk indicator

The third exposure risk indicator accounts for the POI-CBG network based on previous transmission from POIs to home cbgs (Fig. 4). Greater percentage change of outside POIs visits signals a higher exposure risk of residents in a home cbg. Similar to the population activity analysis, percent change stayed below − 40% from the baseline. There were also indications of exposure risk disparity across all urban locations. After the implementation of NPIs, residents from lower-income home cbgs had less percent change compared to those from higher-income home cbgs. This suggests that lower-income households were less able to reduce their exposure risk. The release of NPIs were mixed. As time continued, the results generally showed similar levels of percent change, which suggests that residents from lower-income home cbgs were approaching a similar exposure risk to those of higher-income home cbgs. The percent change of home cbgs in Los Angeles also appeared to converge despite not having a release of NPIs. In contrast, New York City, which did not have a release of NPIs, still had the greatest difference of percent change in the POI network from lower-income cbgs and higher-income cbgs, and this difference even increased by the end of the analysis period.

The correlations further support the findings that home cbgs of lower income and greater percentage of non-white populations had higher comparative exposure risk based on a lesser ability to reduce movement in the POI-CBG network (Fig. 5). Approximately after the implementation of NPIs in Chicago, Houston, and Los Angeles, there is a flip from positive to negative correlations. This signals a shift in the POI-CBG network as lower income households and higher percentage of non-white populations were less able to reduce mobility. For those three cities, correlations were between − 0.15 and − 0.45 for median income levels and between − 0.05 and − 0.45 for non-white populations at a statistically significant p-values. Before NPIs, New York City and Seattle had no significant correlations, but after the NPIs, these cities had correlations between − 0.15 and − 0.30 for median income levels and between − 0.20 and − 0.45 for the non-white populations. The time period of statistically significant correlations varied depending on the city. The disparity in lower-income households remained consistent for Chicago, Houston, and Los Angeles until the beginning of June 2020. In particular, New York City maintained the highest negative correlations at statistically significant p-values until the end of the analysis period which may suggest a great disparity in exposure risk to the CBG-POI network for low income and higher percentage of nonwhite populations.

### Spatial mapping of high exposure risk areas

**Figure 6:**
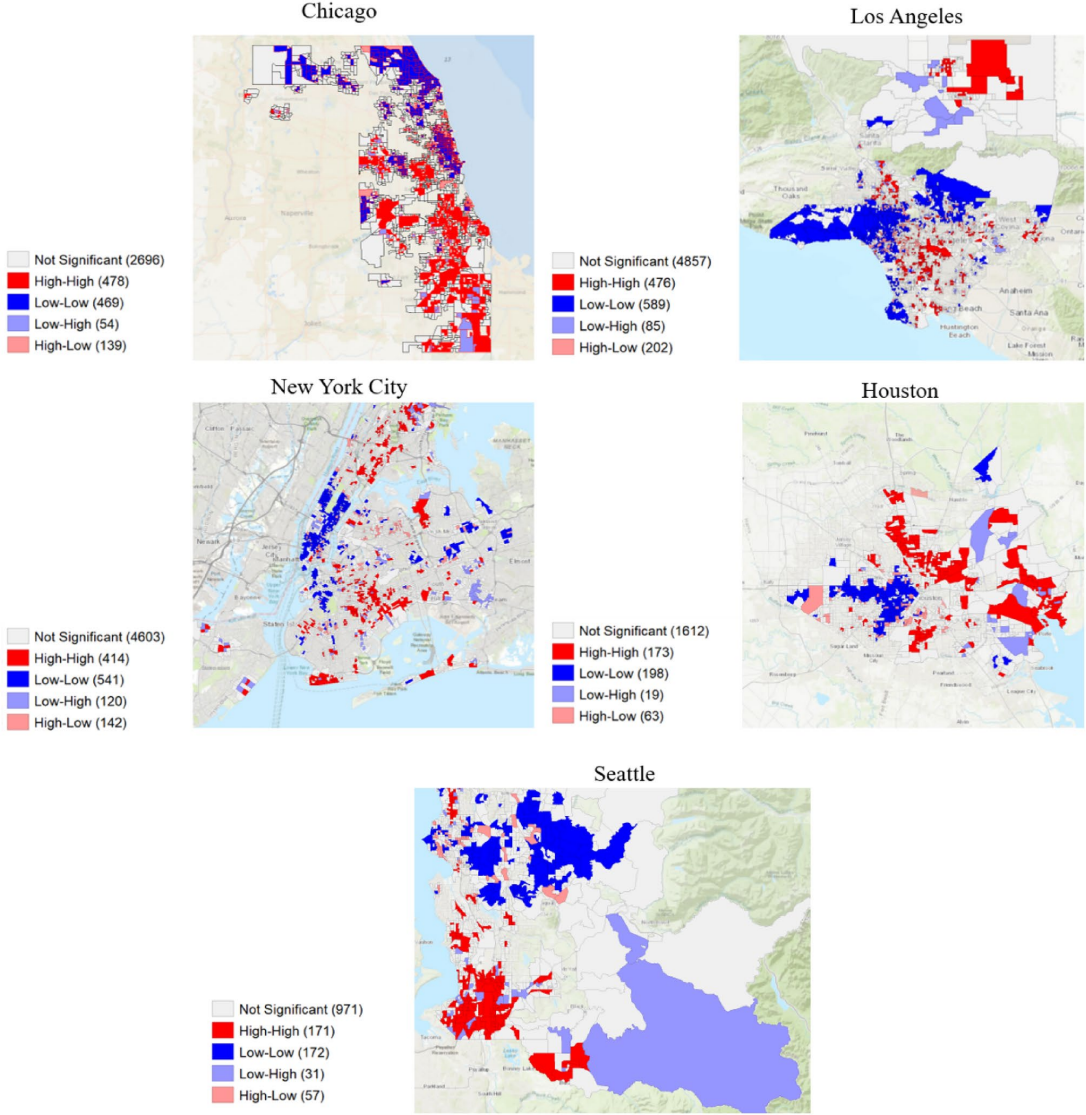
Visualization of exposure risk of the median income levels of cbgs on the POI-CBG network. Spatial clusters are statistically significant for p < 0.05 with the median income levels percentage change from the baseline at week 14. H–H (High–High) clusters are represented by the darkest shade of red and L–L (Low–Low) are represented by the darkest shade of blue. The authors used open-source GeoDa Software (https://geodacenter.github.io/, 1.20.0.0) for calculations and maps^50^.

To visualize the changing spatial clusters of percent change, Moran’s I statistic and spatial maps were calculated for before the implementation of NPIs (January 27th to February 2nd), after the implementation of NPIs (April 6th to April 12th), and, if applicable, after the release of NPIs (June 15th to June 21st). The spatial maps clustered the percentage change of population activity fluctuations and change of visits in POI-CBG network to the median income levels and percentage of nonwhite populations. Table 2 shows the changing Moran’s I correlation coefficients which are statistically significant at p < 0.05 along with the number of H–H and L–L clusters in the POI-CBG network for all urban locations (full information in Supplementary Information E). Figure 6 provides an example of the spatial maps by showing the significant clusters of the five metropolitan cities in the POI-CBG network according to the median income levels.

**Table 2:** Bivariate Moran’s I statistic for POI-CBG network for income groups.

City	Jan 27–Feb 2			Apr 6–12			Jun 15–21		
	Moran’s I	H–H	L–L	Moran’s I	H–H	L–L	Moran’s I	H–H	L–L
**Income groups**									
Chicago	0.091	176	109	0.343	478	469	0.132	234	347
Houston	0.212	167	124	0.283	173	198	0.035	108	143
Los Angeles	0.046	213	172	0.235	476	589	0.003	262	316
New York City	0.039	192	154	0.238	414	541	0.289	375	698
Seattle	0.076	47	41	0.285	171	172	0.099	141	88
**White and non-white groups**									
Chicago	0.126	150	156	0.341	445	547	0.125	186	428
Houston	0.141	116	124	0.077	93	199	− 0.087	59	169
Los Angeles	0.018	179	189	0.118	365	619	− 0.200	180	399
New York City	0.033	158	178	0.225	422	584	0.178	286	758
Seattle	0.021	27	45	0.156	138	160	0.078	105	130

The Bivariate Moran’s I statistic was calculated for the five urban locations for the median income groups and the percentage of white and non-white groups. Jan 27–Feb 2 represents a time period before the stay-at-home policy; Apr 6–12 represents a time period after stay-at-home policy and Jun 15–21 represents a time period following weeks after the stay-at-home policy. H–H represents the number of high–high clusters and L–L represents the number of low–low clusters; both of the number of observed clusters were statistically significant of at least p-values < 0.05.

The results indicated that there were few significant clusters for the second exposure risk of population activity fluctuations, which suggests that the demographic characteristics had little influence on the percent change to POIs from a spatial perspective. However, the results support that there may be a spatial component in the third exposure risk for home cbgs which could be further explored. There is an association with the percentage change in POI-CBG network to the median income levels and non-white populations which varies depending on the urban location. Following the implementation of NPIs, Chicago, Houston, Los Angeles, New York City and Seattle had higher correlation values and an increase of H–H clusters and L–L clusters related to income groups whereas Chicago, Los Angles, New York City, and Seattle had higher correlation values and an increase of H–H clusters and L–L clusters related to white and non-white groups. The number of H–L and L–H slightly increased but not to the same extent (Supplementary Information E).

## Discussion

The COVID-19 pandemic has exacerbated systematic inequalities embedded in the health care system including poor access to medical services, costly medical treatments, inattention to underlying medical conditions, and misinformation and misunderstanding of safety policies^51–53^. The ever-growing body of research literature continues to uncover risk disparities associated with the pandemic, specifically the ability for different populations to follow protective actions which reduce mobility around the community and limit exposure to the virus. Therefore, human mobility data is a valuable resource to understand the potential exposure to COVID-19^54,55^. Several studies have investigated different mobility metrics, such as the ability to stay at home^20,56,57^, the intensity and duration of social distancing^58^, exposure density of different neighborhoods^59^, and the spatiotemporal contact density in particular industries^60^, all of which reveal insights on the increased risk for socially vulnerable populations. However, there remains a knowledge gap of the underlying mechanisms which contribute to such disparities as well as a lack of granular analysis across multiple cities using different indicators of exposure risk.

Thus, in this study, we examined anonymized mobility data and population activity data from two datasets, to measure three indicators of exposure risk indicators to the COVID-19 virus: (1) transmission across ZIP codes, (2) population activity fluctuations for contact at POIs, and (3) the POI-CBG network for previous transmission at POIs back to home cbgs. First, the mobility data was used to create a ZIP code-to-ZIP code origin–destination network to record the inflow measures, or number of trips, to different nodes. Second, the population activity data recorded the fluctuations to POI visits. Third, the population activity data was used to establish a CBG-POI network to record POI visits from different home cbgs. The frequency of inflow measures of the mobility dataset, percent change to POI visits of population activity fluctuations, and percent change of total visits to POIs from different home cbgs through a POI-CBG network all indicate notable separations of mobility and visits patterns.

The significant finding of the research is that there exist disparities in human mobility regarding different income and percentage of nonwhite populations which were measured through three exposure risk reduction. Such disparity in mobility could be a contributing factor to the increased exposure risk to COVID-19 by vulnerable populations^51,53,61^. The findings also suggest an association between the implementation of stay-at-home practices and the disproportionate impacts on different demographic populations. While the research study acknowledges the importance of non-pharmacological interventions, such as stay-at-home policies, which are effective in reducing the contact and transmission of the COVID-19 virus^62,63^, the findings also support literature which has found that staying-at-home may be a privilege primarily held by higher-income demographics^57^. For the first exposure risk, lower-income residents and nonwhite populations were less able to reduce their exposure risk across ZIP codes. For the second and third risk exposures, correlations supported that lower income and greater percentage non-white populations had greater exposure risks for contact at POIs and from home cbgs. The significance of the results also varied across time and location. The implementation of NPIs was associated with differences in exposure risk reduction; however, the release of NPIs did not greatly influence the level of disparity. Indeed, although all urban locations showed instances of exposure risk disparity, New York City maintained the highest negative correlations to low-income and nonwhite populations being less able to reduce their mobility to the end of the analysis period. In addition, the potential spatial connection must be further explored to understand the underlying mechanisms between exposure risk disparity of mobility and population activity. This was shown through the moderate spatial correlations in New York City, Chicago, and Los Angeles for the POI-CBG network.

It is important to also note the potential limitations in the datasets to put the significant findings in context. For one, the resolution and nature of the datasets means that the results do not account for whether people were following all the safety guidelines of CDC, such as maintaining at the recommended six-foot distances and wearing approved masks, but the three exposure risk indicators can be used as proxies for measuring the protective actions of limiting movements around the community and around physical locations. It is also important to note, as with the majority of studies using mobility and location-intelligence, the data imbalance towards individuals and demographics owning smartphones. Given the quantity distribution of the mobility dataset and population activity dataset, the researchers feel that an accurate demographic was captured to measure the different patterns and behaviors of the five urban communities.

In the conversation of social health disparities surrounding the pandemic, Chowkwanyun and Reed^64^ discuss the importance of gathering data and information to develop a “precise picture of how vulnerability is distributed” while also emphasizing the importance of “[contextualizing] such data with adequate analysis”. Though the findings highlight certain individuals and areas with high exposure risk to the virus because of an inability to reduce mobility and population activities, it is critical that researchers, policymakers, and the general public avoid stereotypes and stigmatization associated with socially vulnerable populations, which could delay resources, hinder participation, and limit voices in the recovery process. It is the responsibility for research studies to contextualize the possible factors influencing mobility disparity and exposure risk. While the results do capture and bring awareness to the vulnerability of different populations, they also encapsulate the additional social disparities exacerbated by stay-at-home policies. Such policies, as previously implemented, do not consider that low-income groups and racial-ethnic minorities are more likely to work as essential and frontline workers in addition to having minimal pay, no sick leave, and being uninsured or underinsured^65^. Higher paying jobs may also be more flexible and accommodating to external shocks, such as the COVID-19 pandemic, and thus, they are able to offer work-from-home protocols^66^. On the other hand, those individuals with lower paying jobs would be more restricted in their work options, which could lead to many choosing between income and health.

Various studies have highlighted that socially vulnerable populations have been disproportionately impacted by the COVID-19 pandemic; however, the conversation of how to move forward from these significant impacts and, most importantly, prevent future ones must be centered on the notion that the ability to protect oneself is often a luxury perpetuated by external factors. Proper use of anonymized mobility data and population activity data can shed light on the effectiveness and equitability of closing and reopening policies. Although NPIs demonstrate to be effective in reducing mobility, there may be unintended consequences that must be addressed through careful governmental policies and protections which not only focus on direct connections to viruses but also the underlying mechanisms contributing to such exposure risk disparities.

## Supplementary Information

Supplementary Information.

## Data Availability

The data that support the findings of this study are available from SafeGraph and StreetLight Data, but restrictions apply to the availability of these data, which were used under license for the current study. The data can be accessed upon request submitted on StreetLight Data and SafeGraph. Other data we use in this study are all publicly available.

## Code availability

The code that supports the findings of this study is available from the corresponding author upon request.
