# Supplementary material for: Human activity and mobility data reveal disparities in exposure risk reduction indicators among socially vulnerable populations during COVID-19 for five U.S. metropolitan cities
Source: Sci Rep. 2022 Sep 22;12:15814. doi: 10.1038/s41598-022-18857-7 (PMC9500070; doi:10.1038/s41598-022-18857-7)
Supplement: Supplementary file 1 — Supplementary Information. [file 41598_2022_18857_MOESM1_ESM.docx]

**Supplementary Information**

**Authors:** **Natalie Coleman^1^, Xinyu Gao^2^ , Jared DeLeon^3^, Dr. Ali Mostafavi^4^**

**Affiliations:**

^1^ Ph.D. Student, Zachry Department of Civil and Environmental Engineering, Urban Resilience.AI Lab, Texas A&M University, College Station; email: [ncoleman@tamu.edu](mailto:ncoleman@tamu.edu)

^2^ Urban Resilience.AI Lab, Texas A&M University, College Station; email: [xy.gao@tamu.edu](mailto:xy.gao@tamu.edu)

^3^ Urban Resilience.AI Lab, Texas A&M University, College Station; email: [jareddeleon@tamu.edu](mailto:jareddeleon@tamu.edu)

^4^ Associate Professor, Zachry Department of Civil and Environmental Engineering, Urban Resilience.AI Lab, Texas A&M University, College Station; e-mail: [amostafavi@civil.tamu.edu](mailto:amostafavi@civil.tamu.edu)

**Table of Contents**

Supplementary Information A. Implementation of NPIs across five urban locations

Supplementary Information B. Sociodemographic Information of Selected Urban Locations

Supplementary Information C. In Degree/ Out Degree Percent Change- Cook County

Supplementary Information D. Sensitivity Analysis of Income Bins for Population Activity Data

Supplementary Information E. Bivariate Spatial Clustering for Contact at POIs

Supplementary Information F. Percent Difference to In Degree Values

## **Implementation of NPIs across five urban locations**

Washington, King County (Seattle)

- Non-essential services closure 3/16/2020 ~ 5/5/2020
- Shelter-in-place 3/23/2020 ~ 6/1/2020

California, Los Angeles (Los Angeles)

- Non-essential services closure 3/19/2020 ~ 5/25/2020
- Shelter-in-place 3/19/2020 ~

Illinois, Cook County (Chicago)

- Non-essential service closure 3/21/2020 ~ 5/29/2020
- Shelter-in-place 3/21/2020 ~ 5/29/2020

New York City

- Non-essential services closure 3/22/2020 ~ 6/8/2020
- Shelter-in-place 3/22/2020 ~

Texas, Harris County (Houston)

- Non-essential services closure 3/24/2020 ~ 5/1/2020
- Shelter-in-place 3/23/2020 ~5/1/2020

## **Sociodemographic Information of Selected Urban Locations**

The mobility data and population activity data were merged in different ways to income and racial-ethnic datasets. The StreetLight data used a distribution of demographic information based on the most updated 2019 American Community Survey data. The mobility data obtained from StreetLight was analyzed with the following income groups: (1) < $20,000, (2) $20,000–$49,999, (3) $50,000–$99,999, (4) $100,000–$149,999, (5) $150,000–$199,999, and (6) ≥$200,000. It used the following six racial-ethnic groups, which follows the United States Office of Management and Business (OMB) standards: (1) White-only, (2) Black or African American, (3) American Indian (also known as Native American) or Alaska Native, (4) Asian, (5) Native Hawaiian/ Other Pacific Islander, and (6) Hispanic or Latino. The percentage of White-only, Black or African American, American Indian or Alaska Native, Asian, and Native Hawaiian/ Other Pacific Islander populations total to 100 percent; however, the percentage of Hispanic or Latino populations overlaps with other categories. In contrast, population activity data obtained from SafeGraph was not already connected to demographic data. Instead, the researchers manually merged the SafeGraph data with demographic information of 2019 American Community Survey data at a cbg level. The values used were continuous median income levels and percentage of non-white populations. Table B1 summarizes the median demographics of the collected data while Figure B1 shows the distribution of median household income. The data is collected at a county level for each urban location. New York City is made up of the following five boroughs: Bronx, Brooklyn, Manhattan, Queens and Staten Island.

**Table B1.** Sociodemographic Information from U.S Census Quick Tables

| **Urban Locations** | **Median of the Household (in 2019 dollars)** | **White alone, percent** | **White alone, not Hispanic or Latino, percent** |
| --- | --- | --- | --- |
| Cook County (Chicago) | $58,247 | 50.0% | 33.3% |
| Harris County (Houston) | $52,338 | 57.0% | 24.4 % |
| Los Angeles County (Los Angeles) | $68,044 | 70.7% | 26.1% |
| New York City (New York) | $63,998 | 42.7% | 32.1% |
| King County (Seattle) | $92,263 | 67.3% | 63.8% |
|  |  |  |  |

**
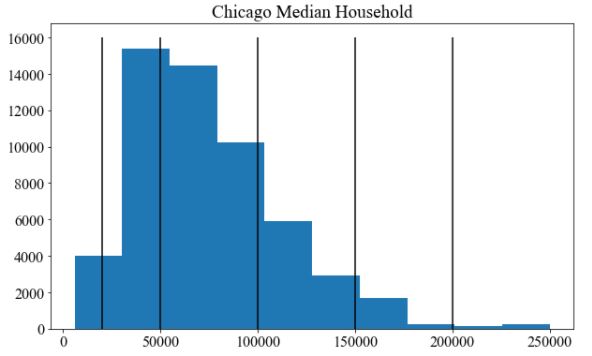

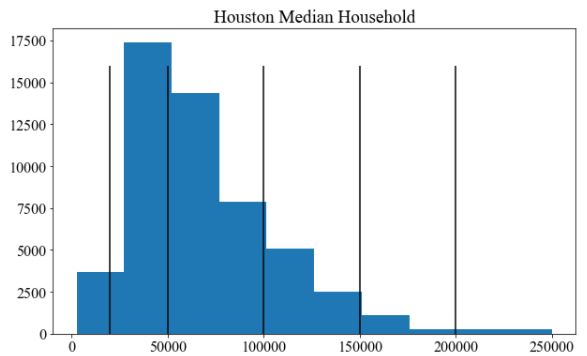

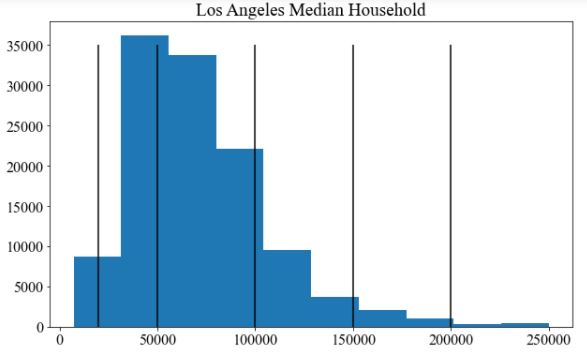

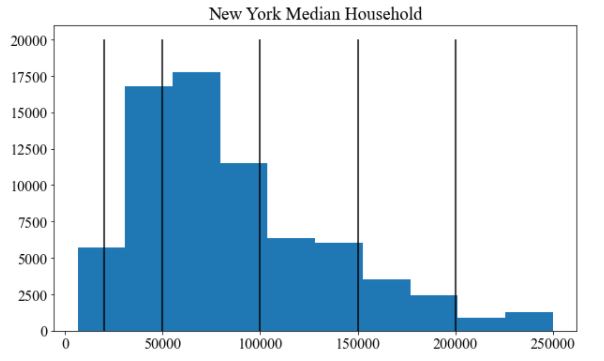

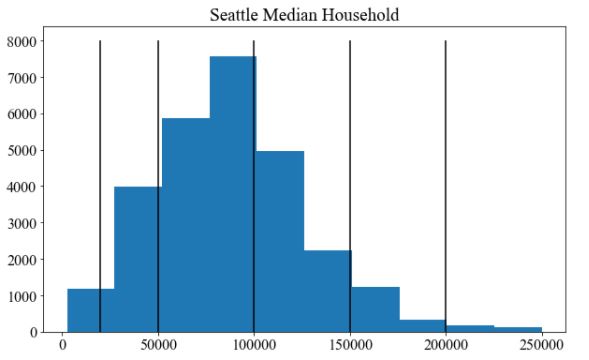
**

**Figure B1.** Median household income of the five urban locations


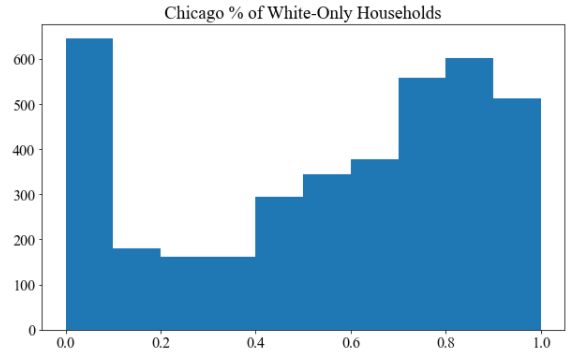

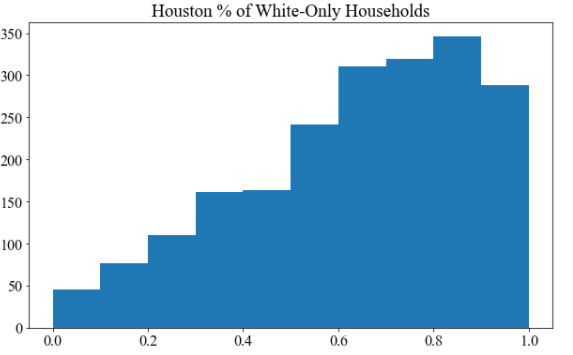


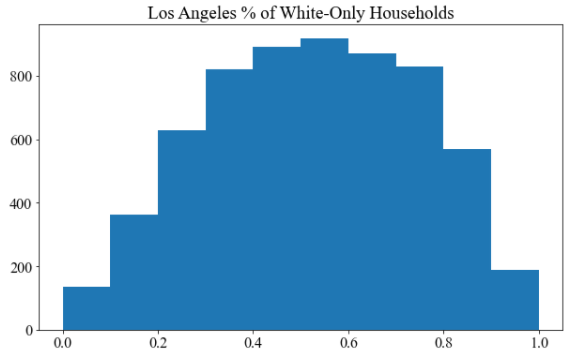

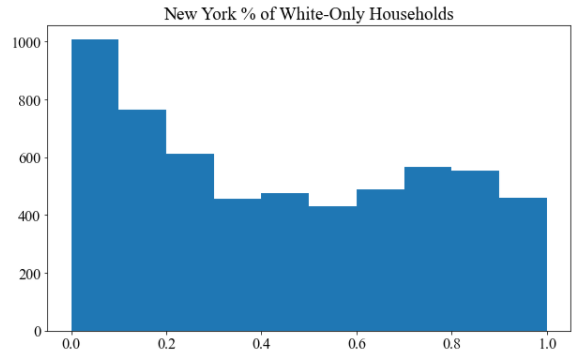


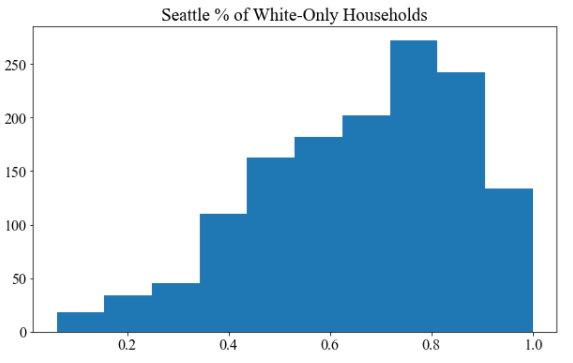


**Figure B2.** Percentage of white-only populations of the five urban locations

## **In Degree/ Out Degree Percent Change- Cook County**

**a)**
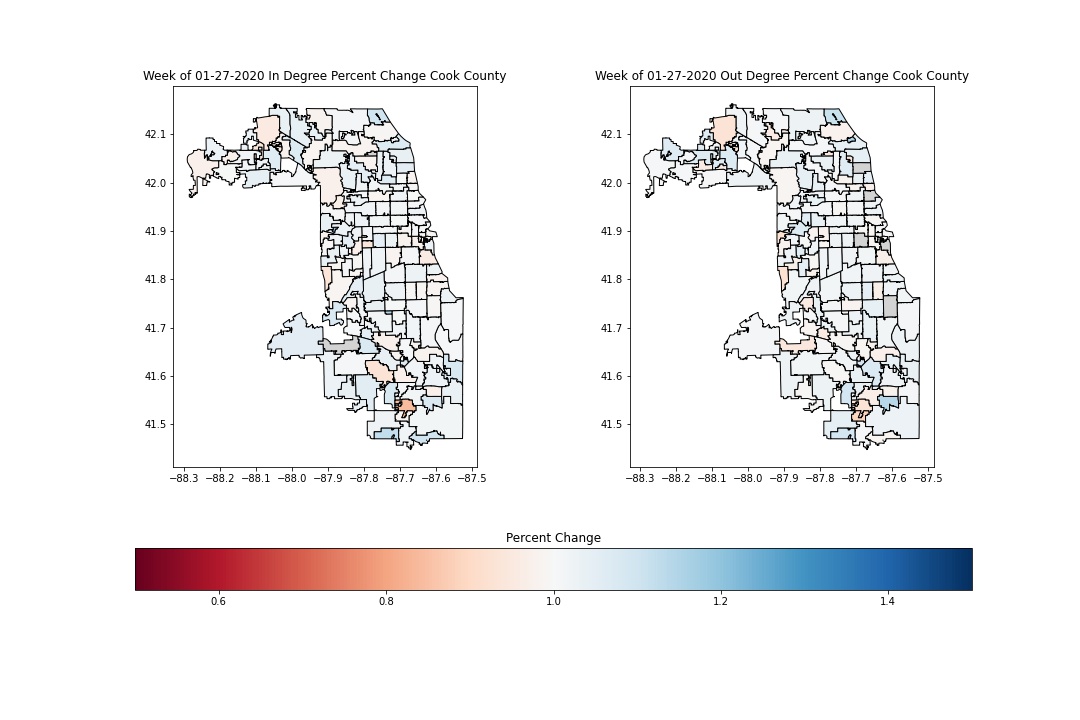


**b)**
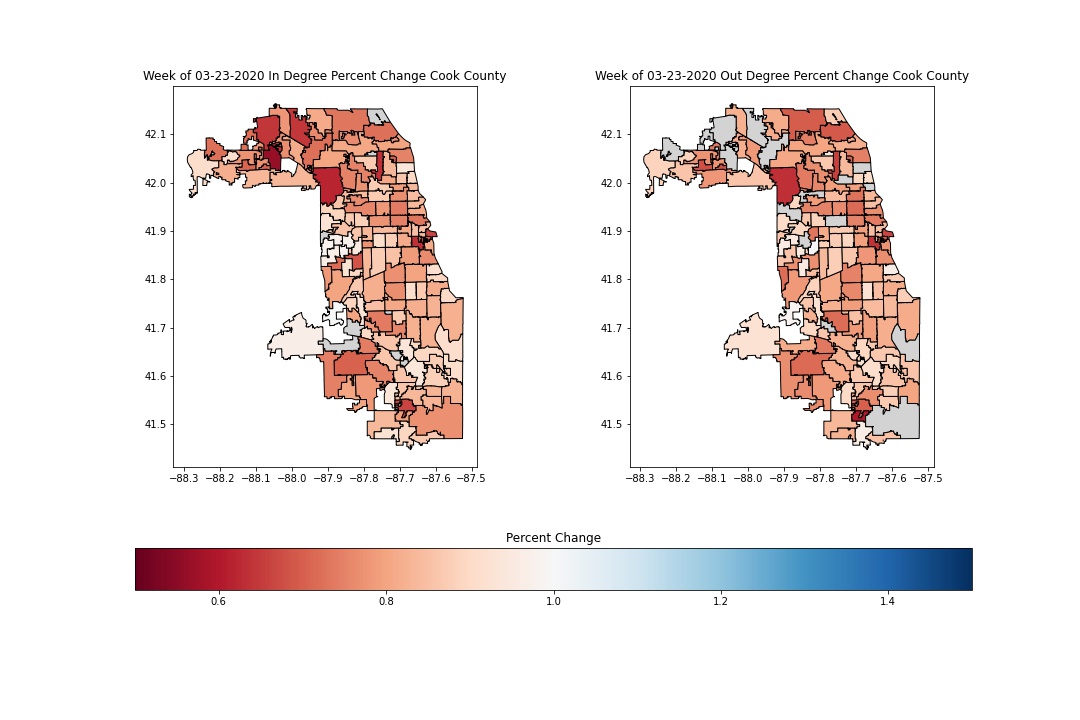


**Figure C1.** In Degree and Out Degree Percent Change of Cook County as an Example of O-D network. The images of the O-D network use the third week of Jan. (Jan. 20^th^ – Jan 26^th^) as a baseline to the percent change of In Degree and Out Degree values, which are measures of inflow and outflow to the nodes. a) represents the week of Jan. 27^th^ – Feb. 2^nd^ while b) represents the week of Mar. 23^rd^ – Mar. 29^th^.

## **Sensitivity Analysis of Income Bins for Population Activity Data**

Sensitivity analysis was conducted to ensure that the binning of the income groups did not significantly influence the final results. The left side of each figure shows the results of the second exposure risk indicator for the population activity fluctuations and the right side of each figure. The top row of subfigures have five bins that represent five equal percentiles: 0-20^th^ , 20-40^th^ , 40-60^th^, 60-80^th^, and 80-100^th^. The middle row of subfigures have six bins that represent six equal percentiles: 0-16^th^, 16-33^rd^, 33-50^th^, 50-66^th^, 66-83^rd^, 83-100^th^. The bottom row of subfigures have seven bins that represent seven equal percentiles: 0-14^th^, 14-29^th^, 29-43^rd^, 43-57^th^, 57-71^st^, 71-85^th^, 85-100^th^. Examining the bins, the same trends appear for all the cities. This means that the graphs with the five bins, six bins, and seven bins similarly rank the income groups, and the income groups follow similar trends.


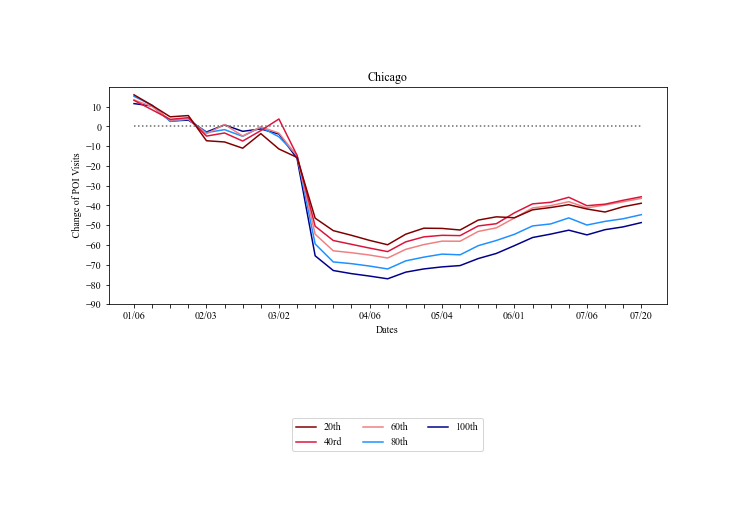

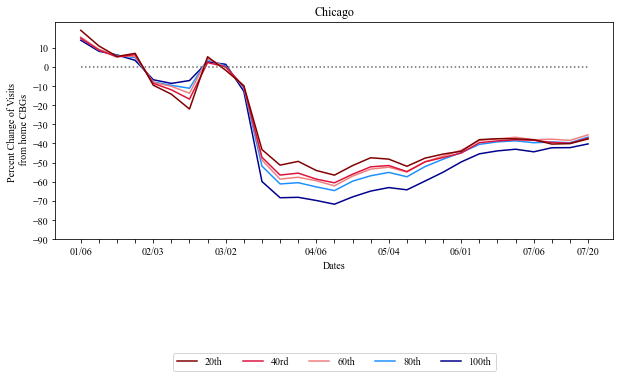


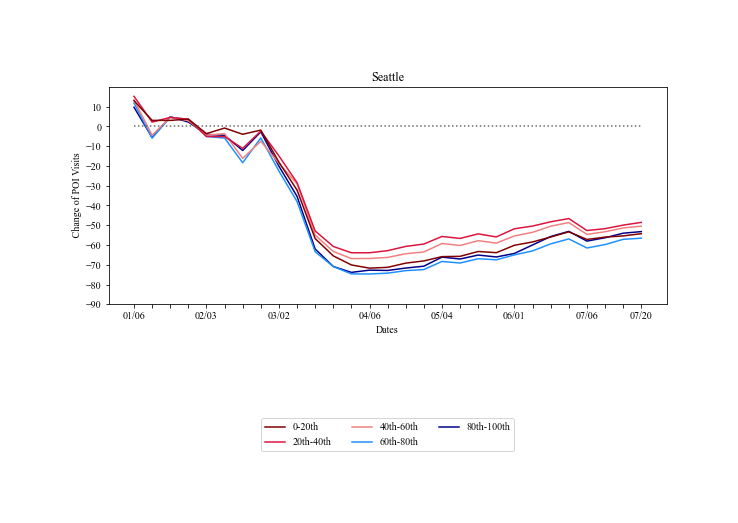


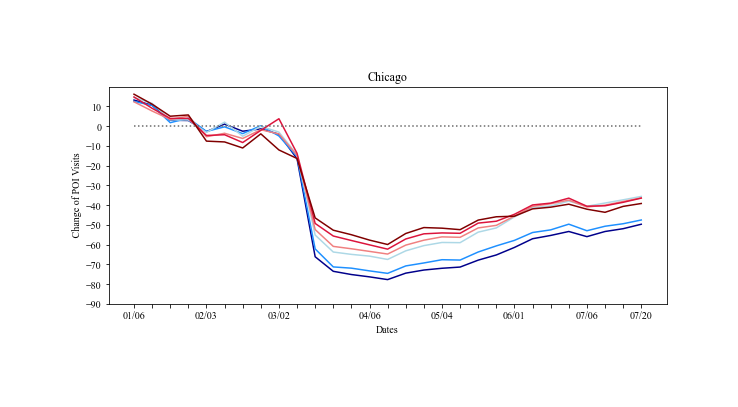

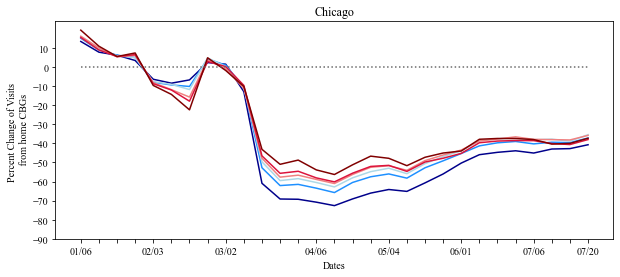


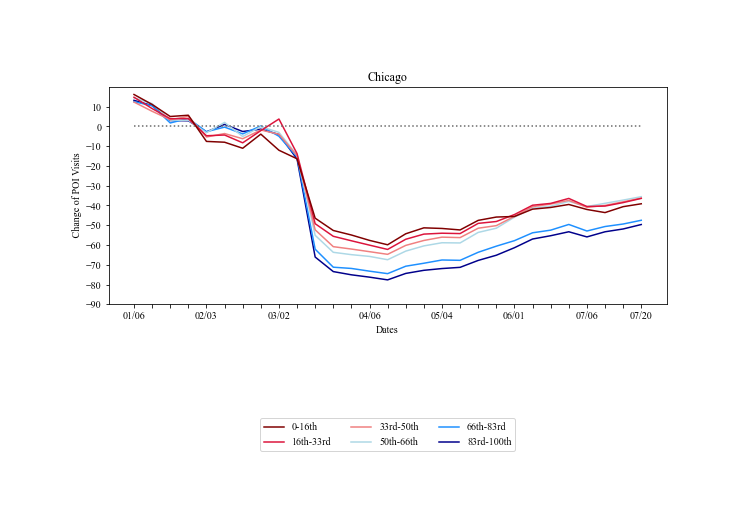


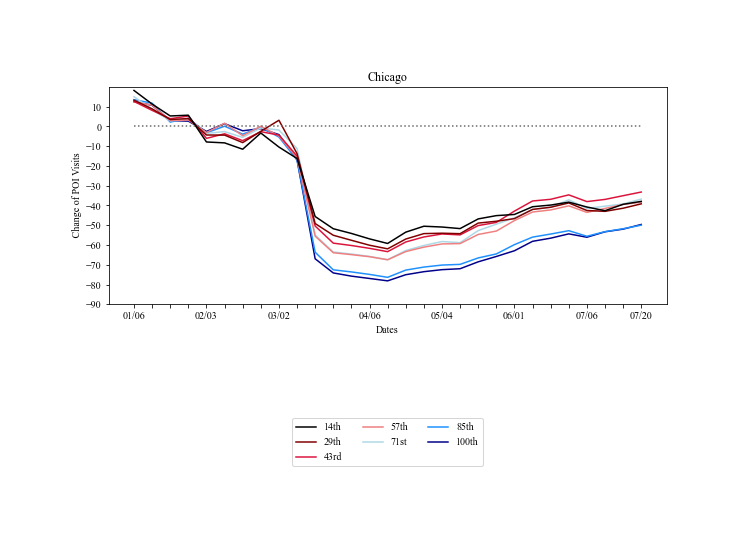

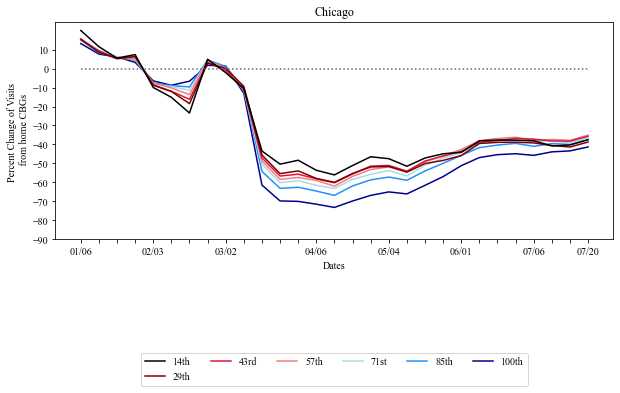


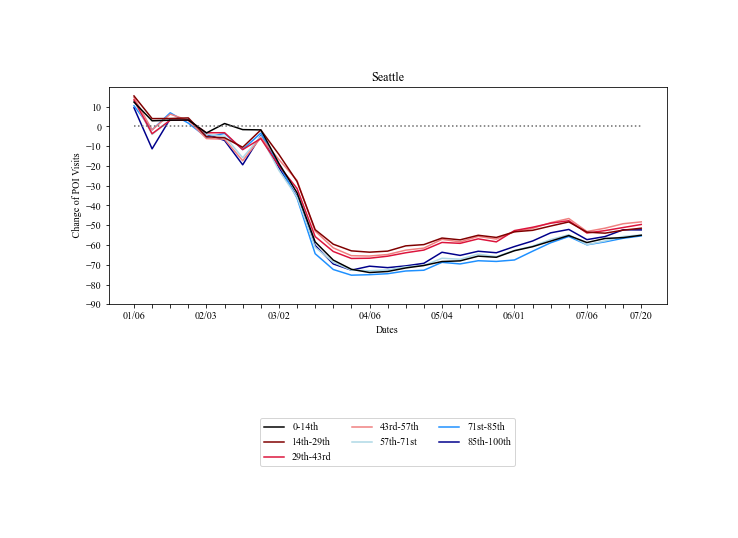


**Figure D1.** Population activity fluctuations and POI-CBG network for Chicago


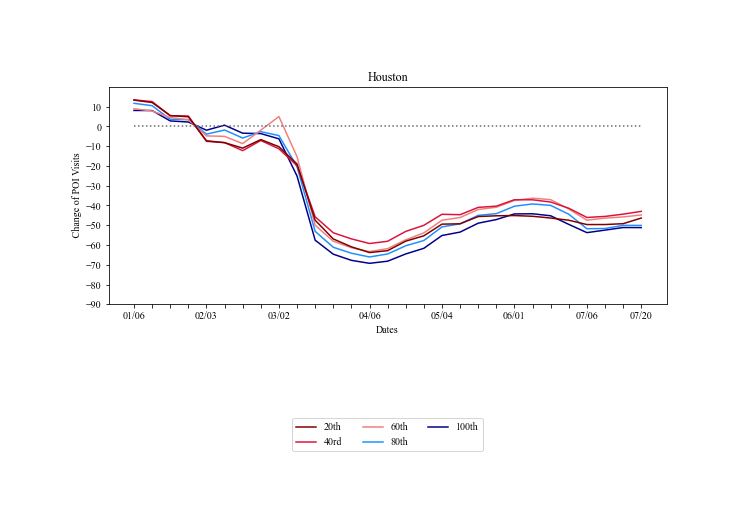

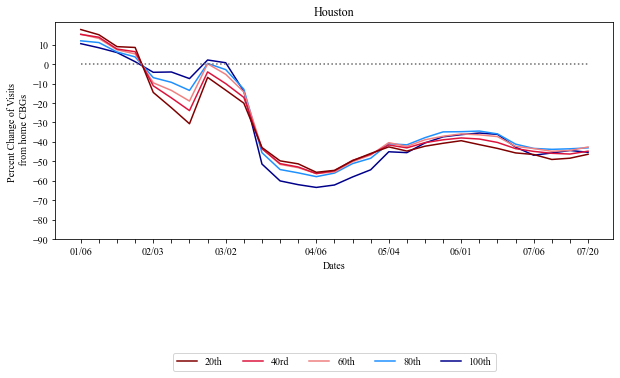


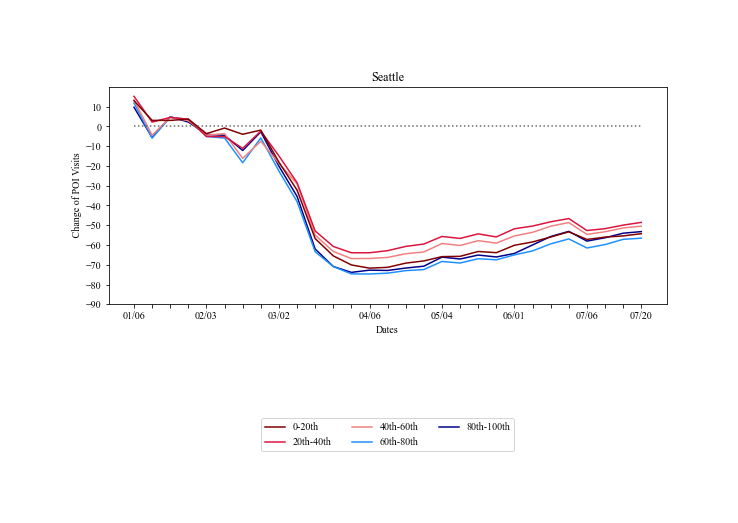


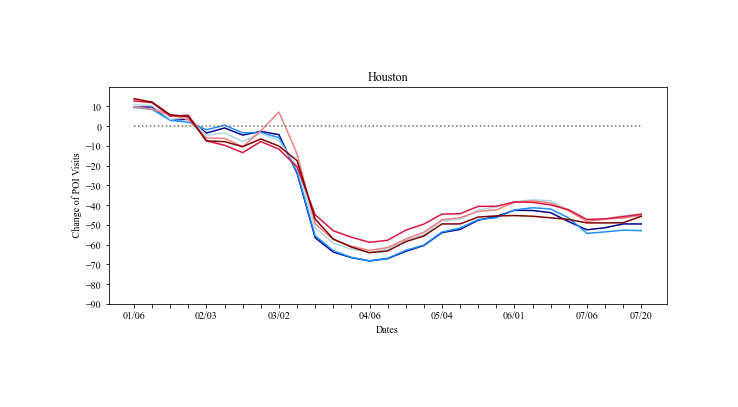

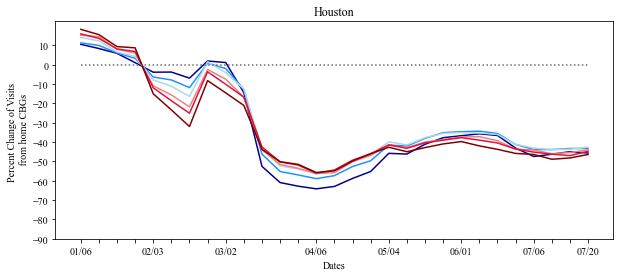


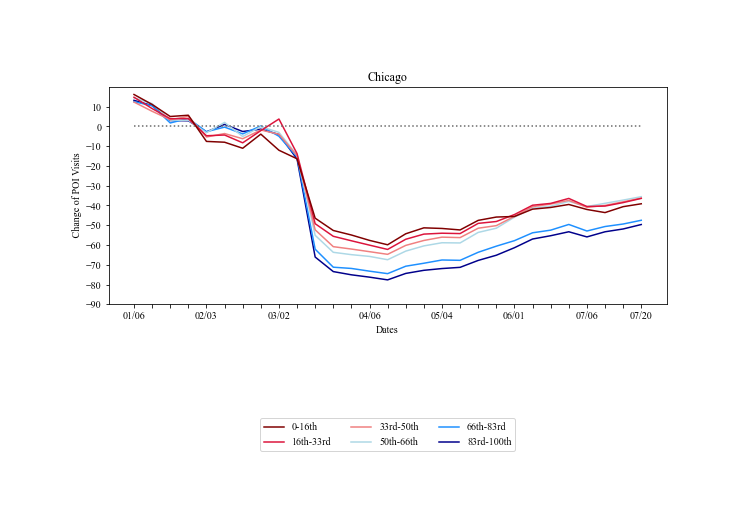


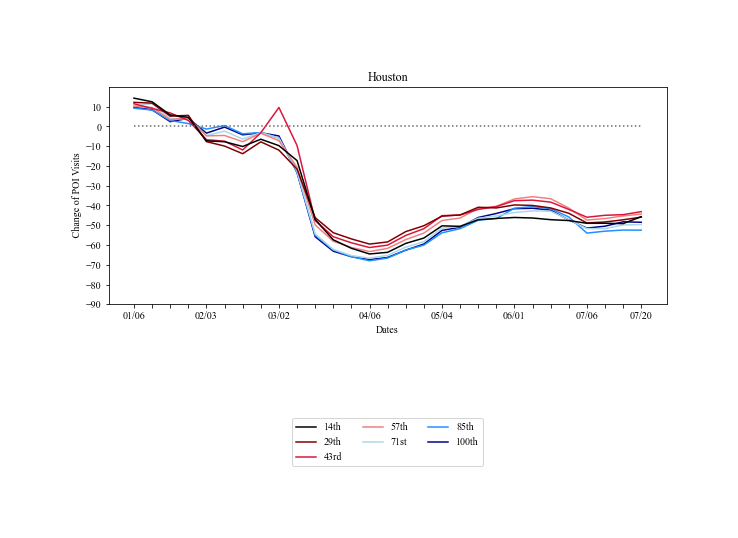

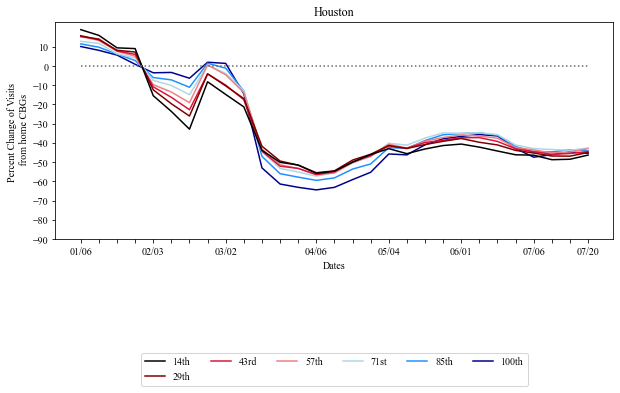


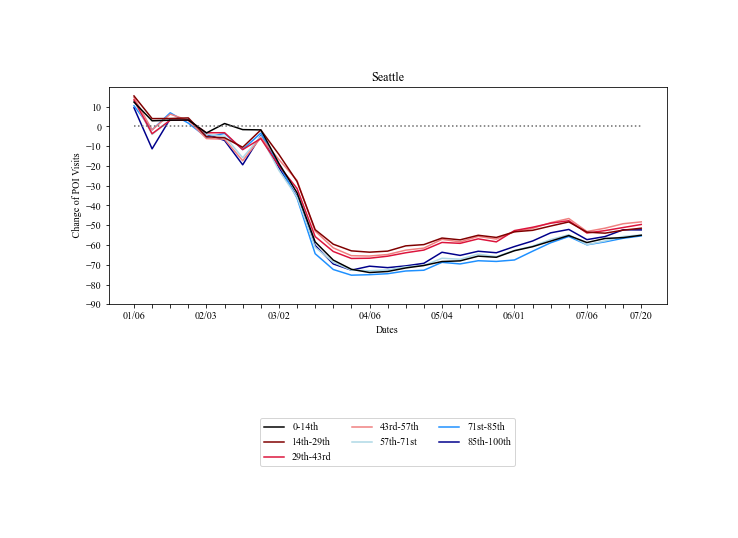


**Figure D2.** Population activity fluctuations and POI-CBG network for Houston


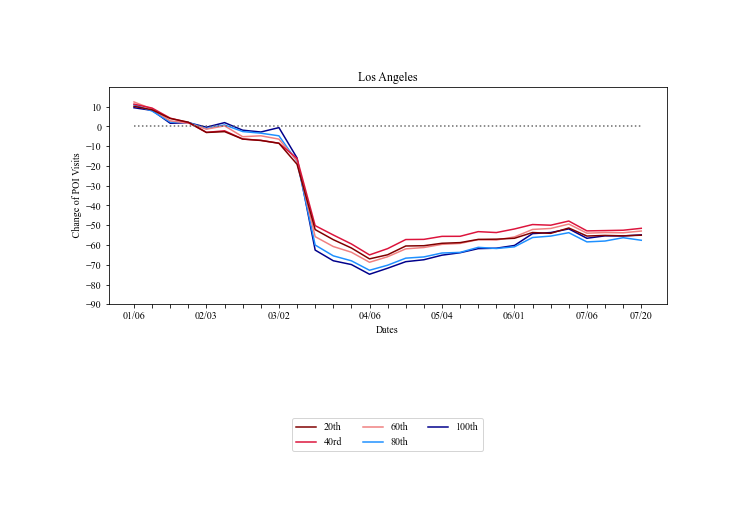

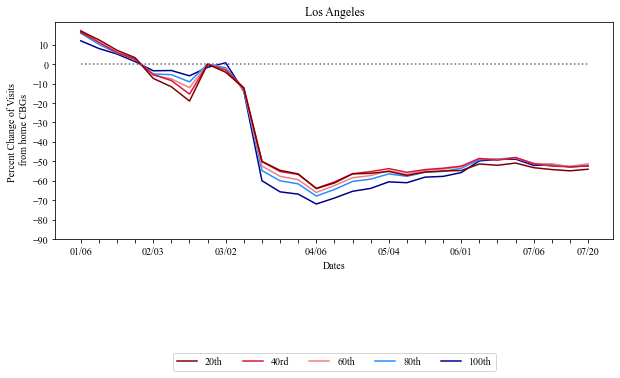


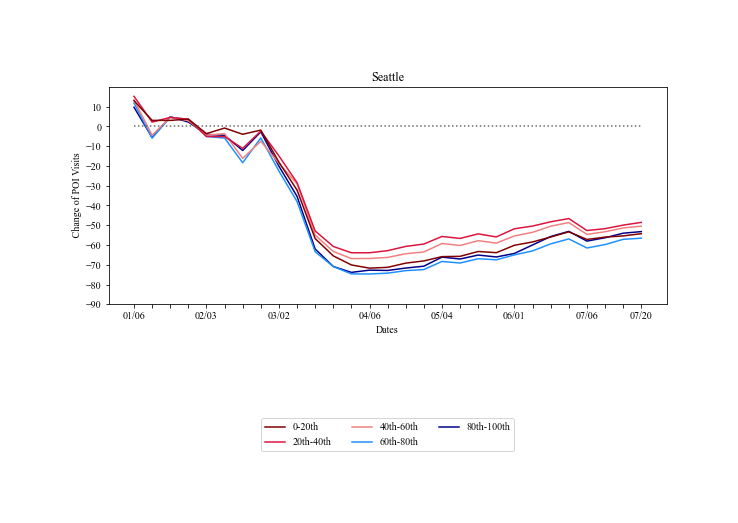


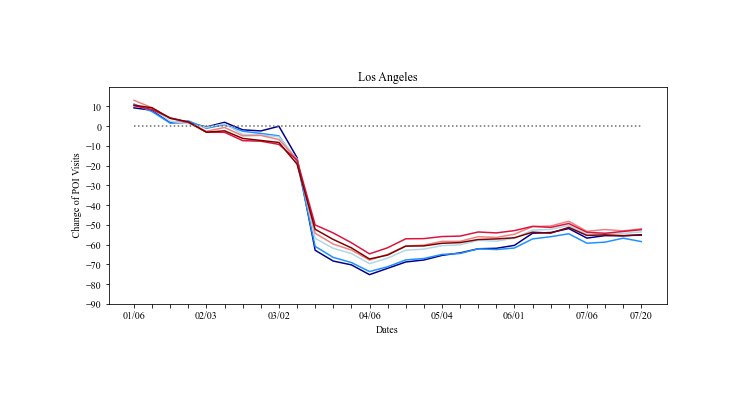

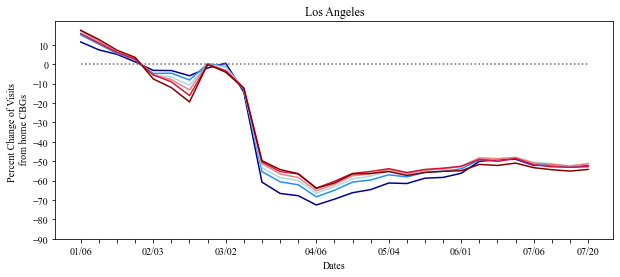


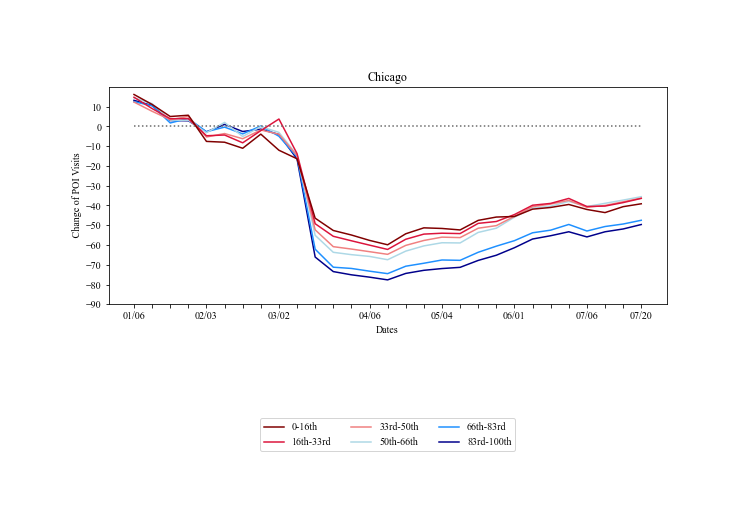


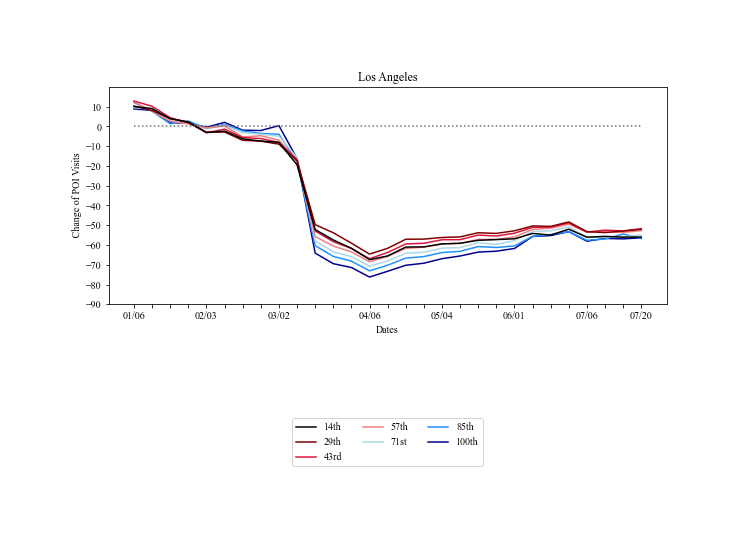

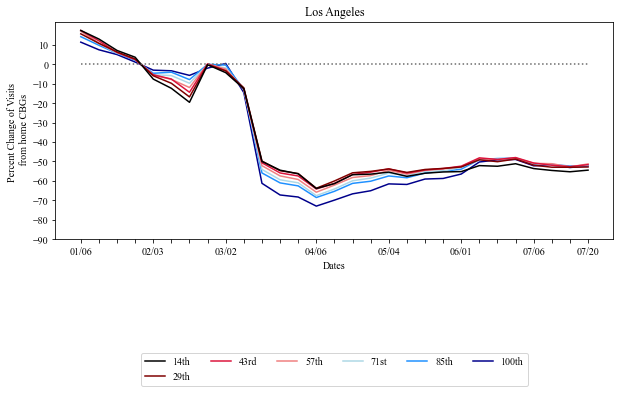


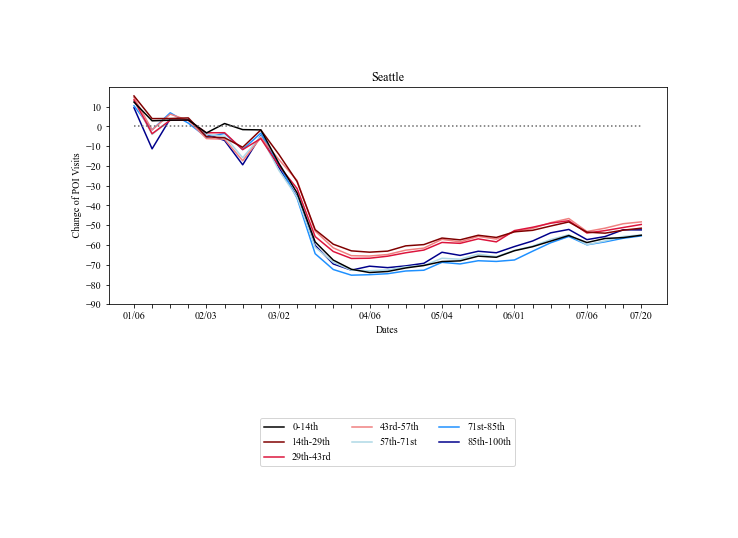


**Figure D3.** Population activity fluctuations and POI-CBG network for Los Angeles


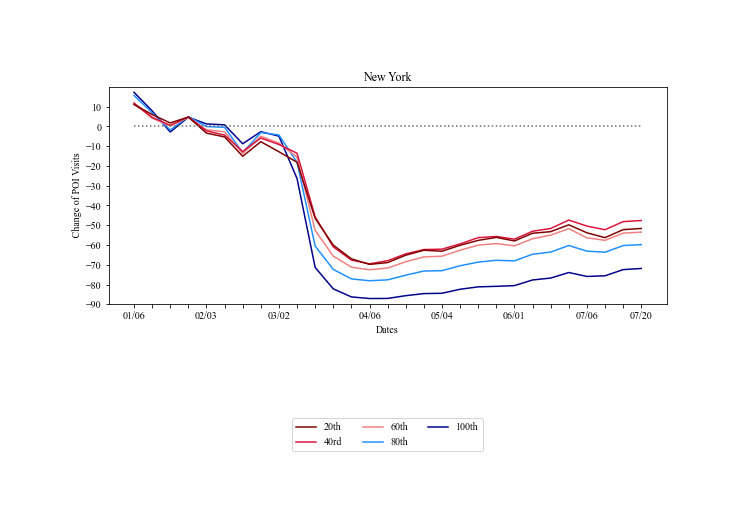

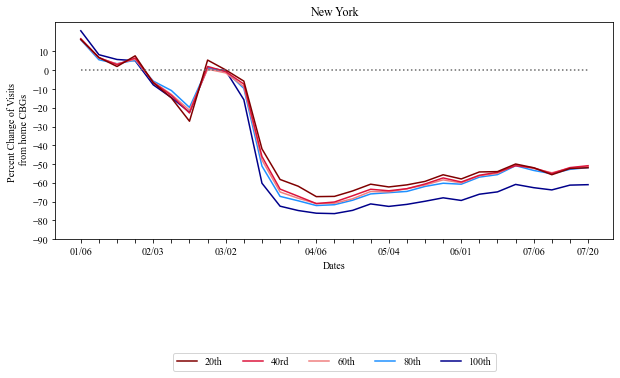


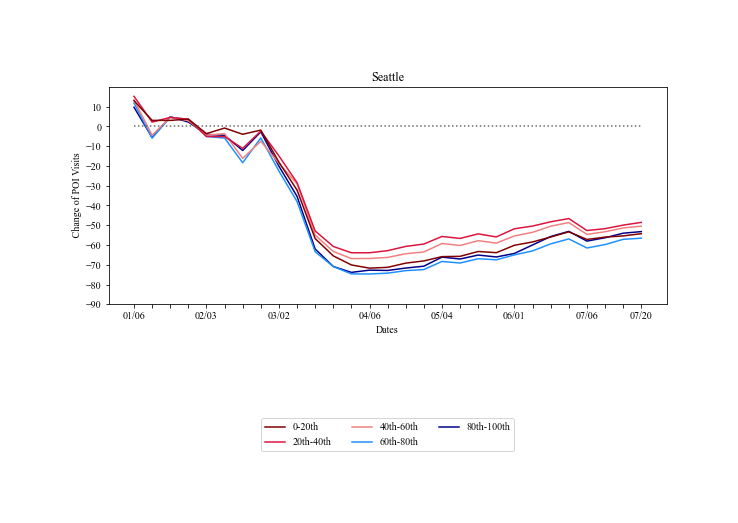


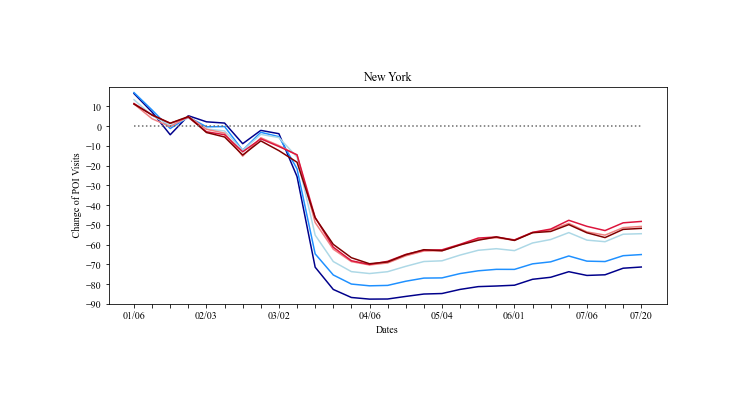

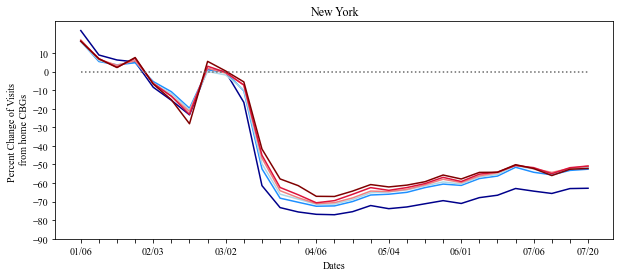


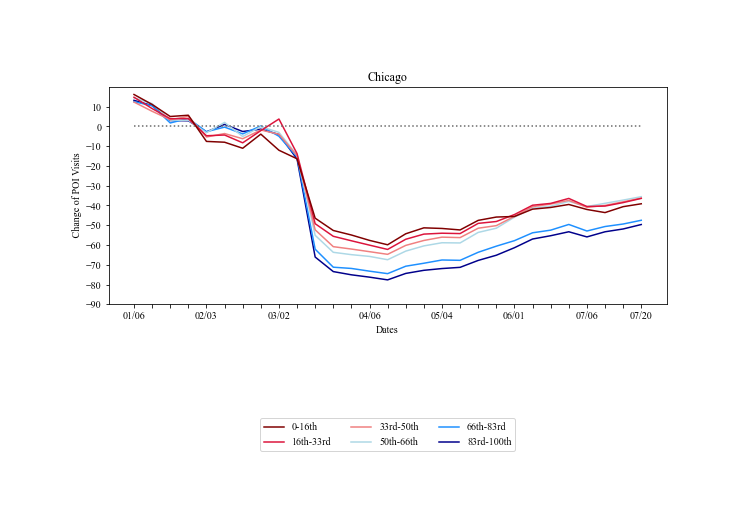


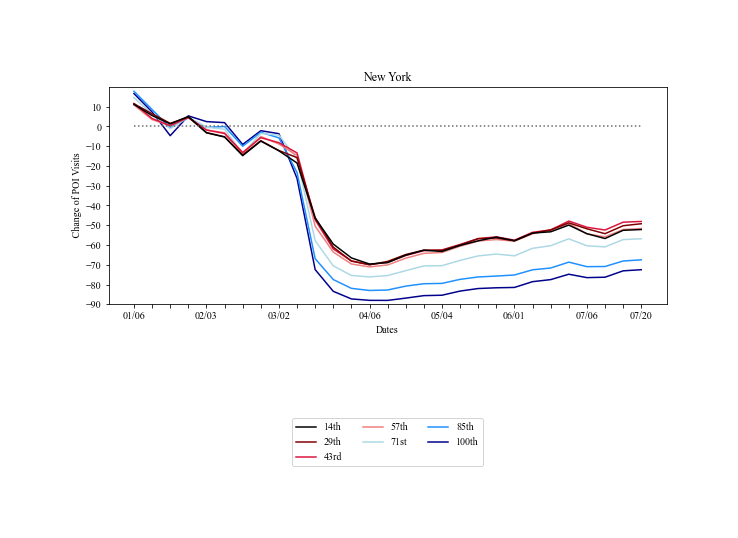

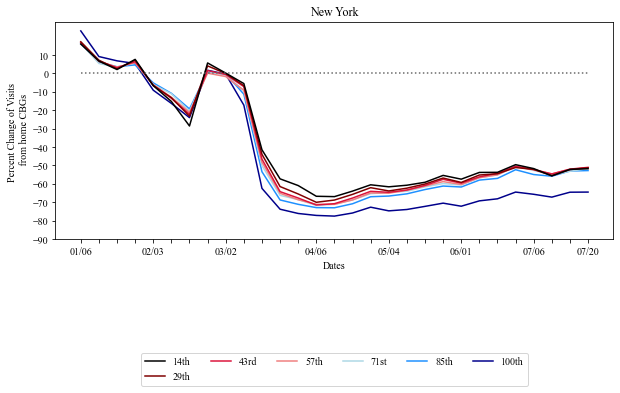


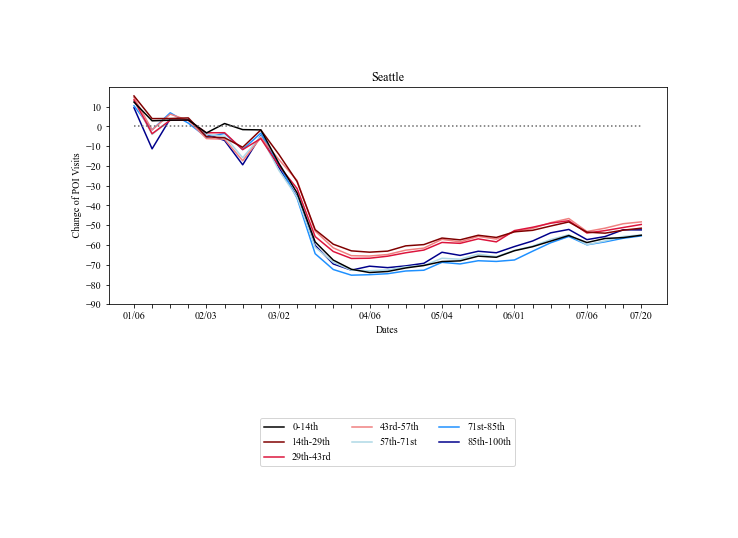


**Figure D4.** Population activity fluctuations and POI-CBG network for New York


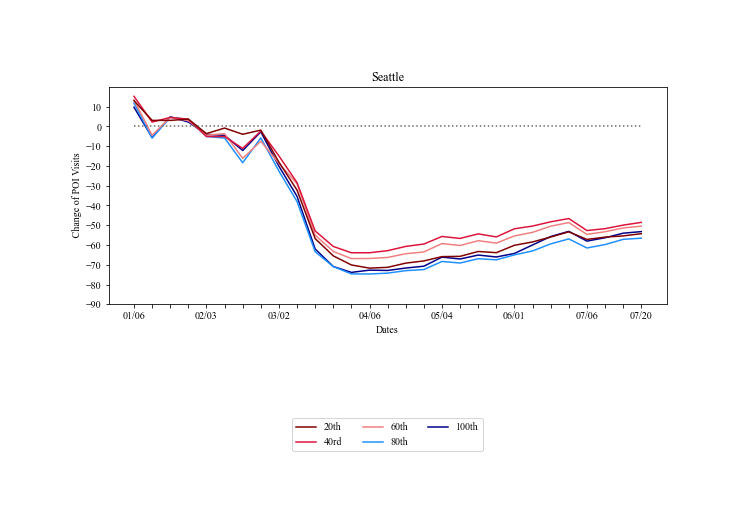

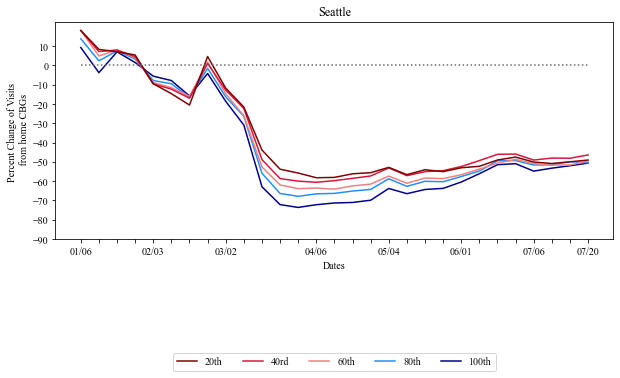


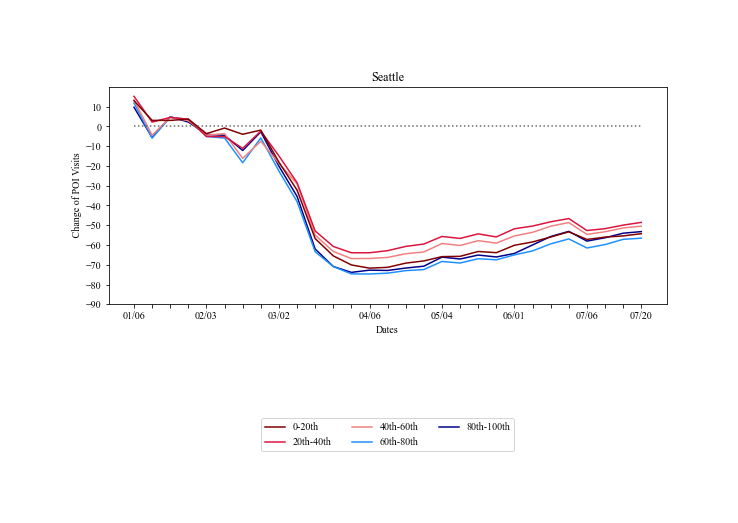


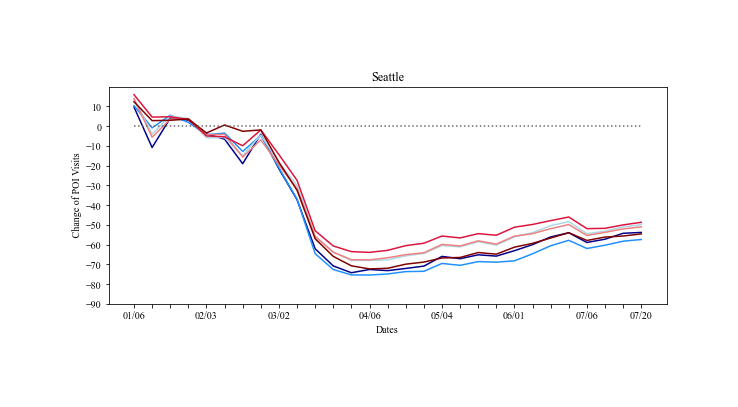

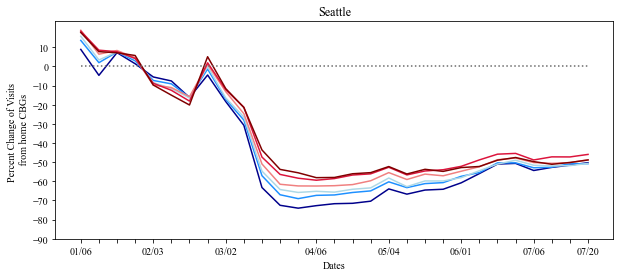


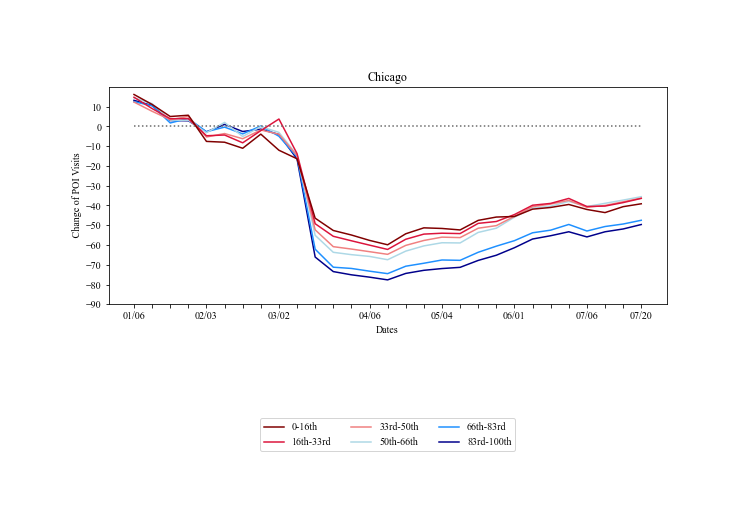


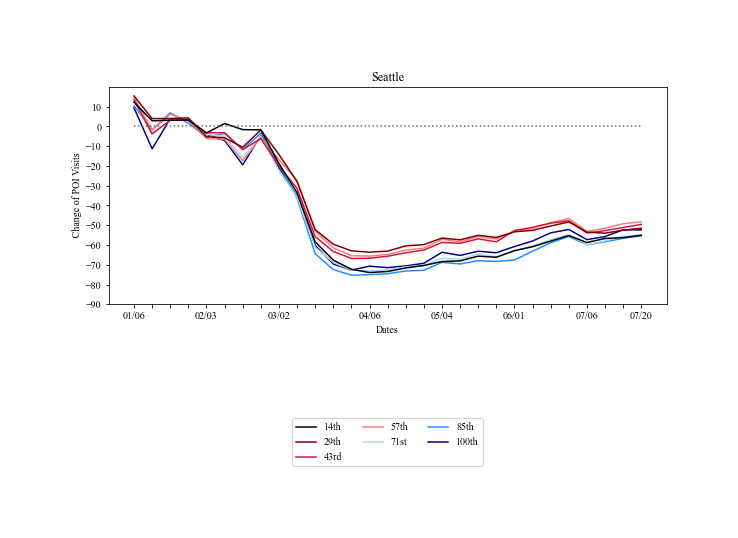

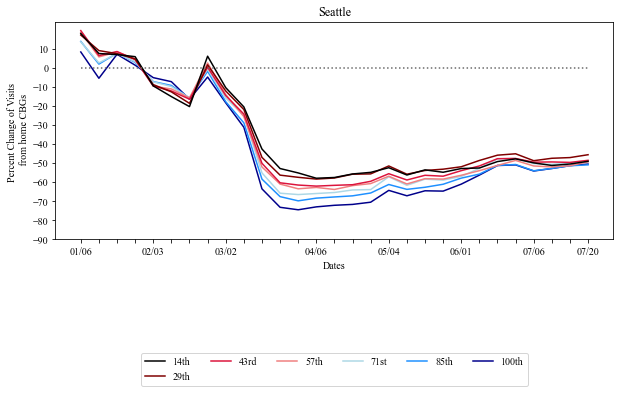


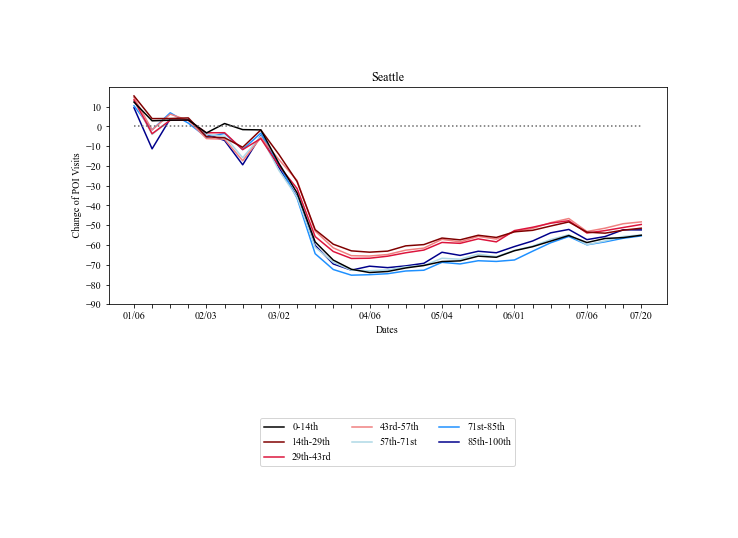


**Figure D5.** Population activity fluctuations and POI-CBG network for Seattle

## **Bivariate Spatial Clustering for Contact at POIs**

Figures E1- E10 are the Bivariate LISA spatial clusters and the Moran’s I values. The maps compare two variables: (1) continuous median income levels or the percentage of non-white populations against the (2) population activity fluctuations and POI-CBG network. High-High clusters represent high exposure risk and high social vulnerability (low income and greater percentage of non-white populations. Nonsignificant areas have a spatial clustering of at least p>0.05. Undefined areas have missing values, where missing values could be the non-recording of visits or missing information of the social groups. Neighborless areas refers to the isolation of specific cbg(s) due to spatial weight; in this case, the Moran’s I will remove those isolations. PC_4 is the percent change from the baseline for Jan 27^th^,2020 to Feb 2^nd^, 2020. PC_14 is the percent change from the baseline for April 6^th^ , 2020 to April 12^th^, 2020 and PC_24 is the percent change from the baseline for June 15^th^ , 2020 to June 21^st^, 2020. The results are also summarized in Tables E1-E4.

**Software Reference:** The authors created Figures E1-E10 using GeoDa software, a open-source analysis software developed by Dr. Luc Anselin and his team in UChicago. The following is the citation for the software: Anselin, L., I. Syabri, and Y. Kho, *GeoDa: An introduction to spatial data analysis*, in *Geographical Analysis*. 2006. <https://geodacenter.github.io/>. 1.20.0.0

1.
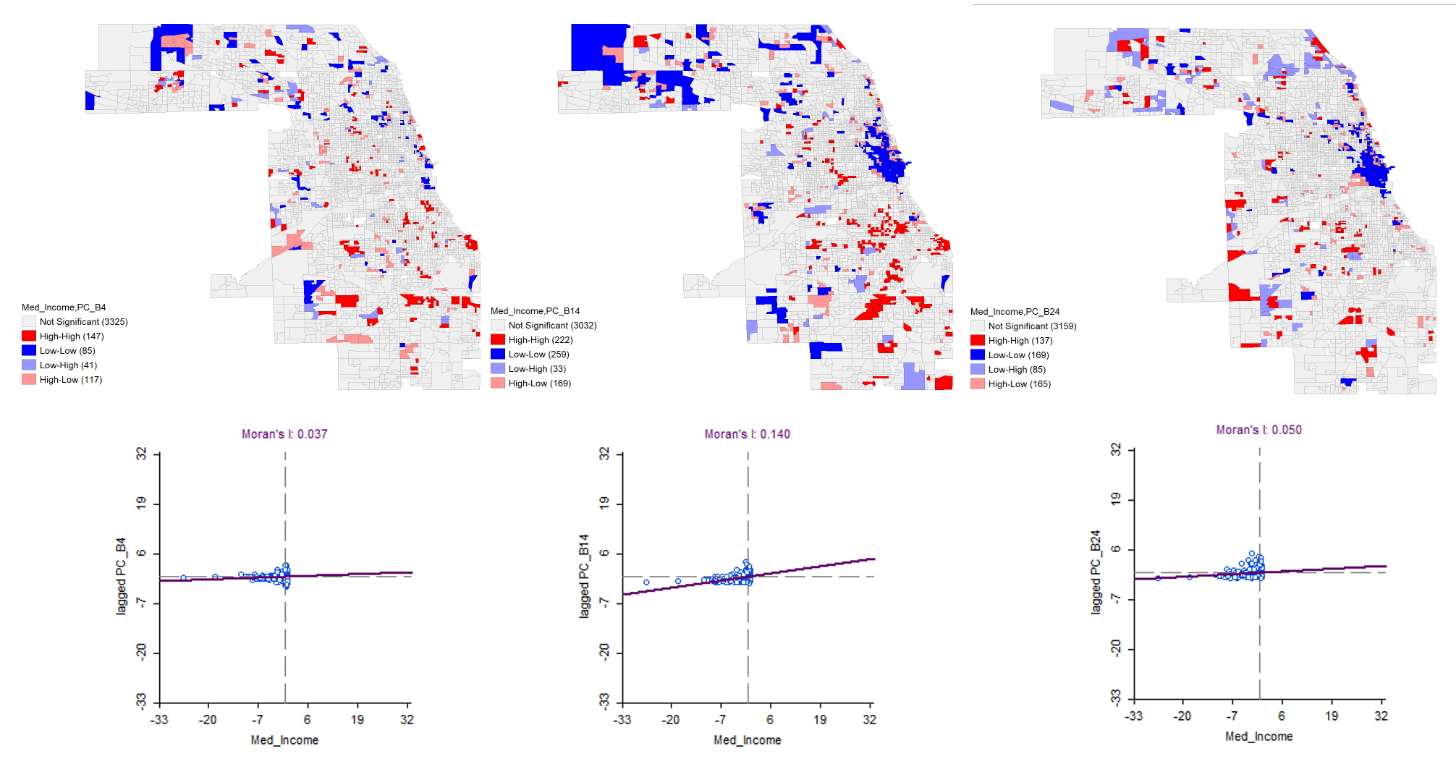

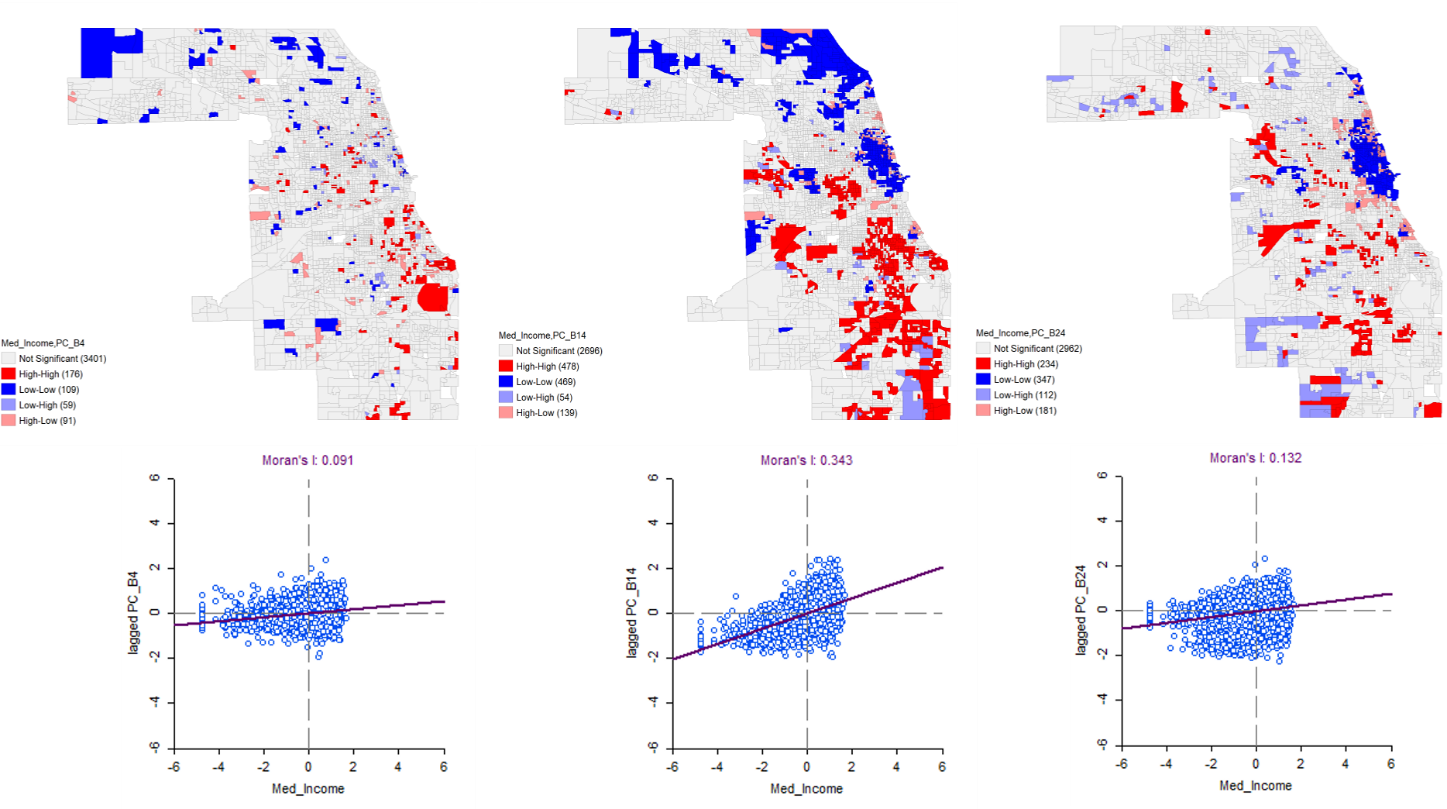


**b)**

**Figure E1a.** Spatial clusters of median income and population activity fluctuations in Chicago

**Figure E1b.** Spatial clusters of median income and POI-CBG network in Chicago

**a)**
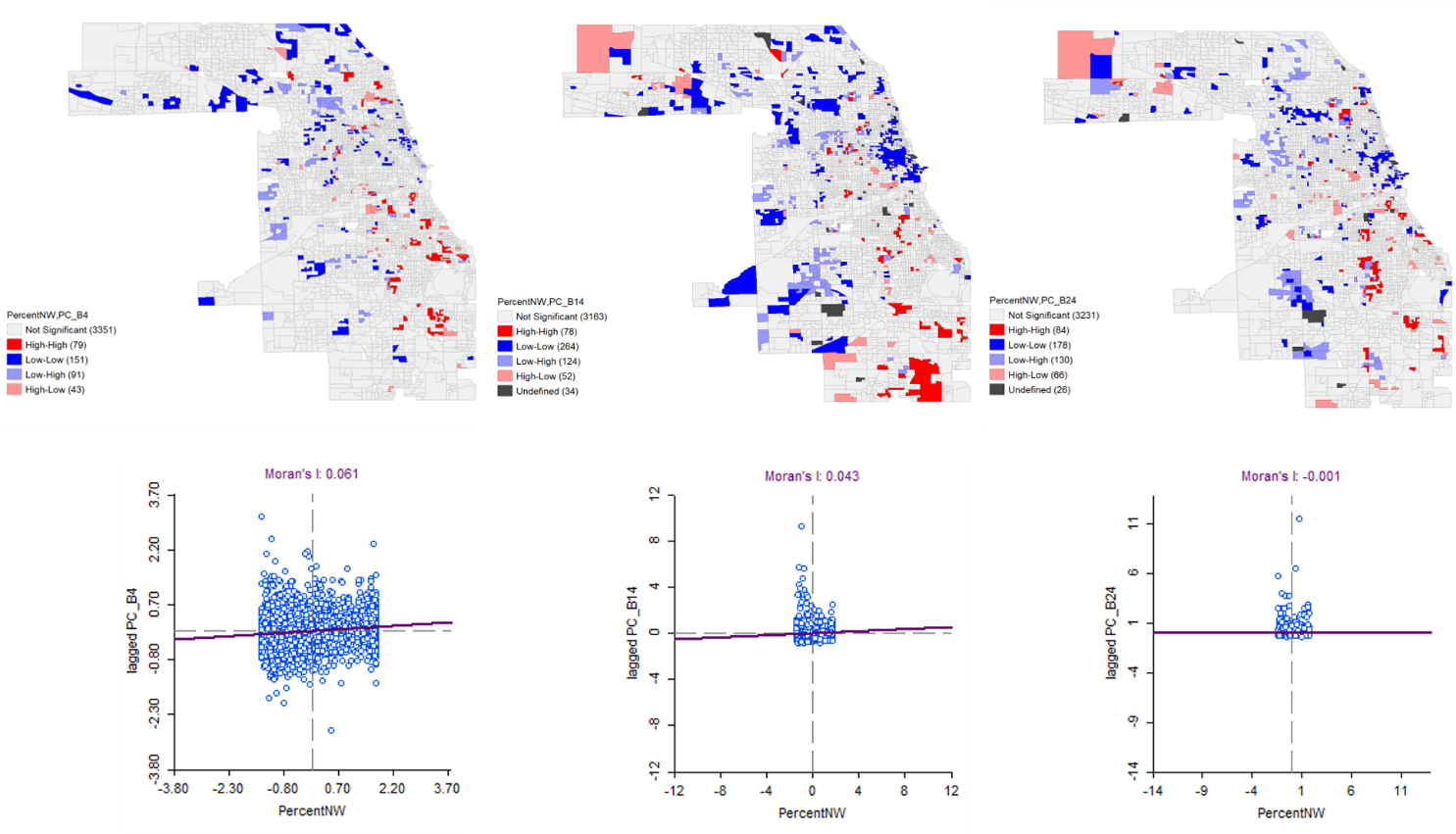

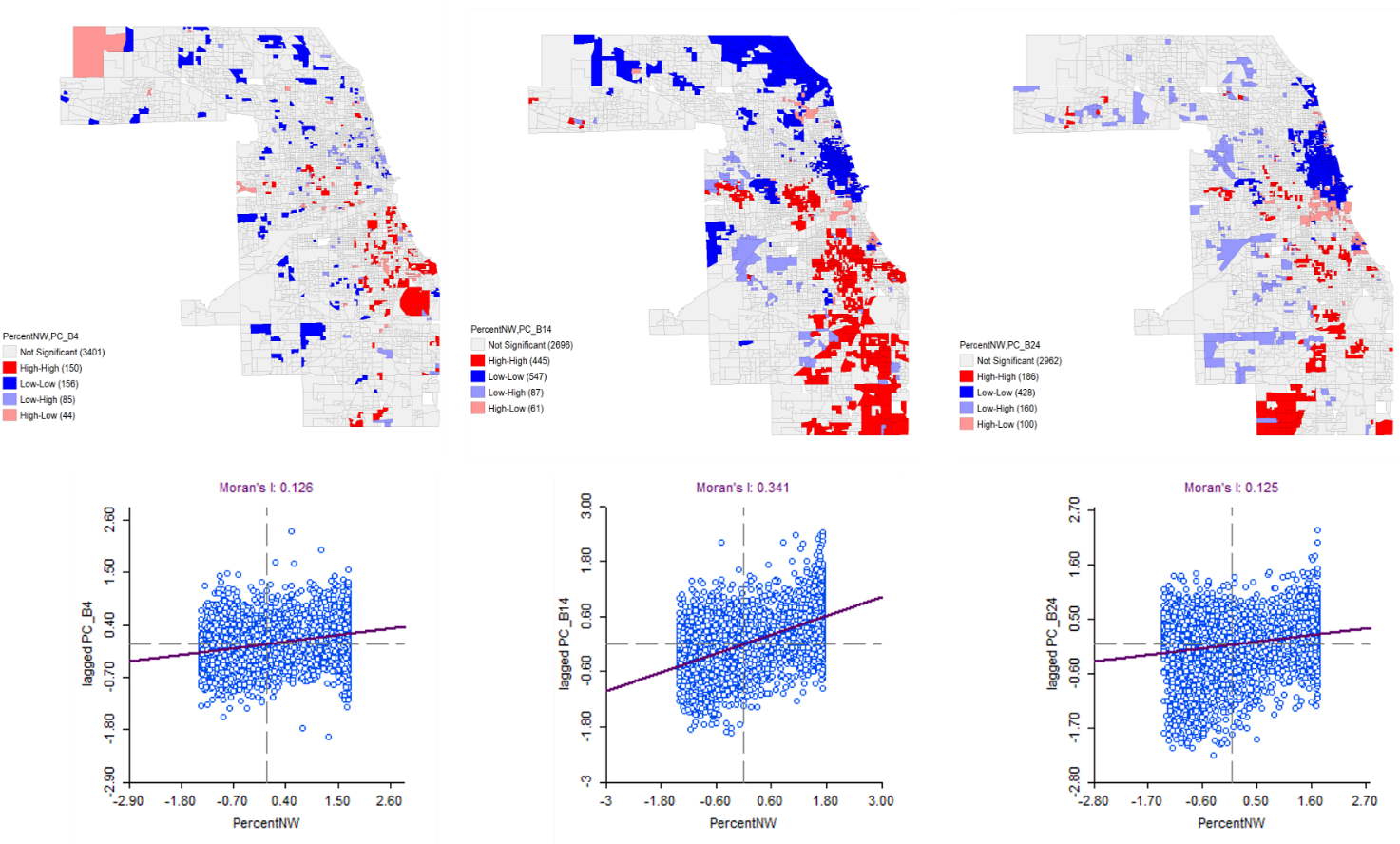


**b)**

**Figure E2a.** Spatial clusters of non-white populations and population activity fluctuations in Chicago

**Figure E2b.** Spatial clusters of non-white populations and POI-CBG network in Chicago

**a)
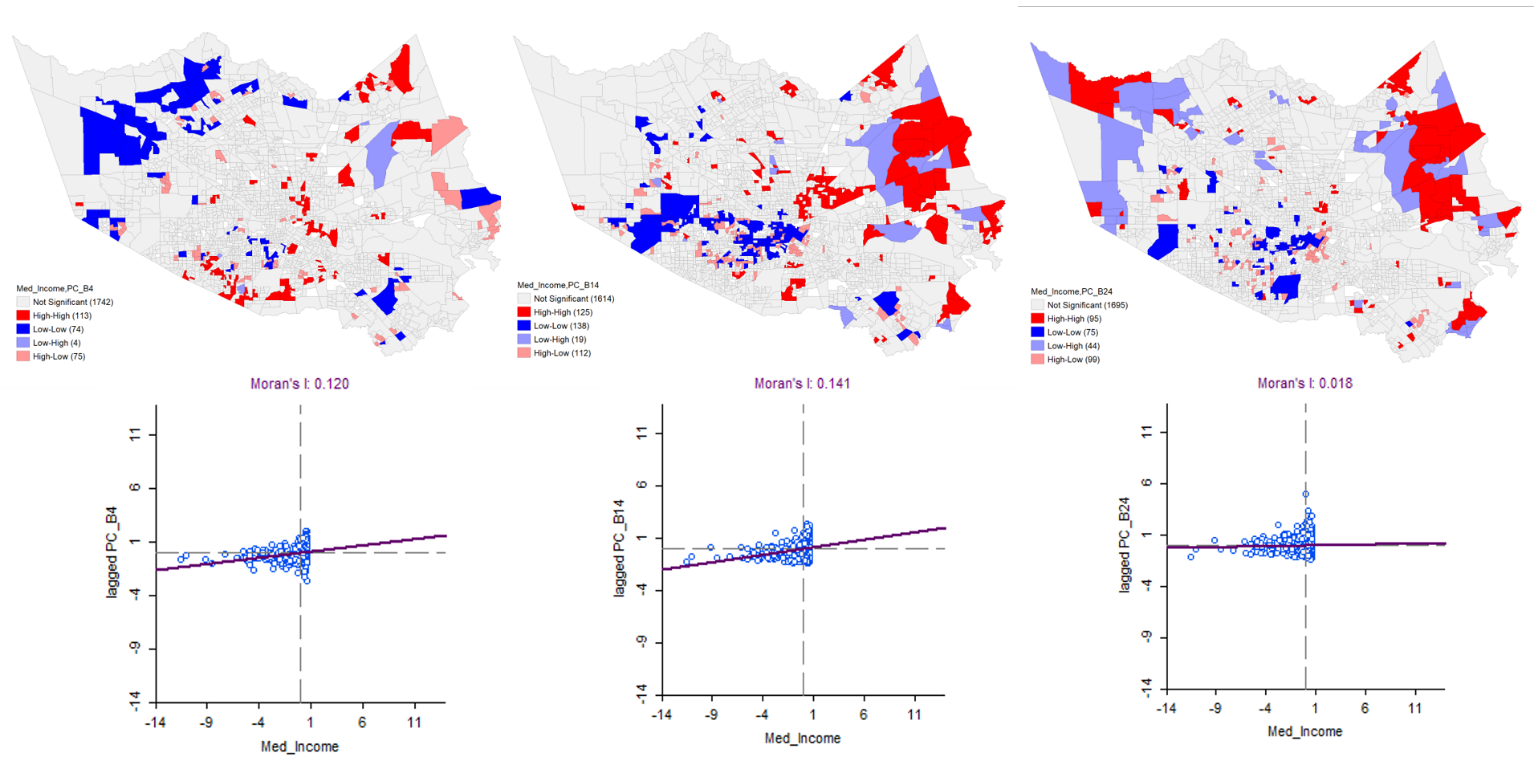
**
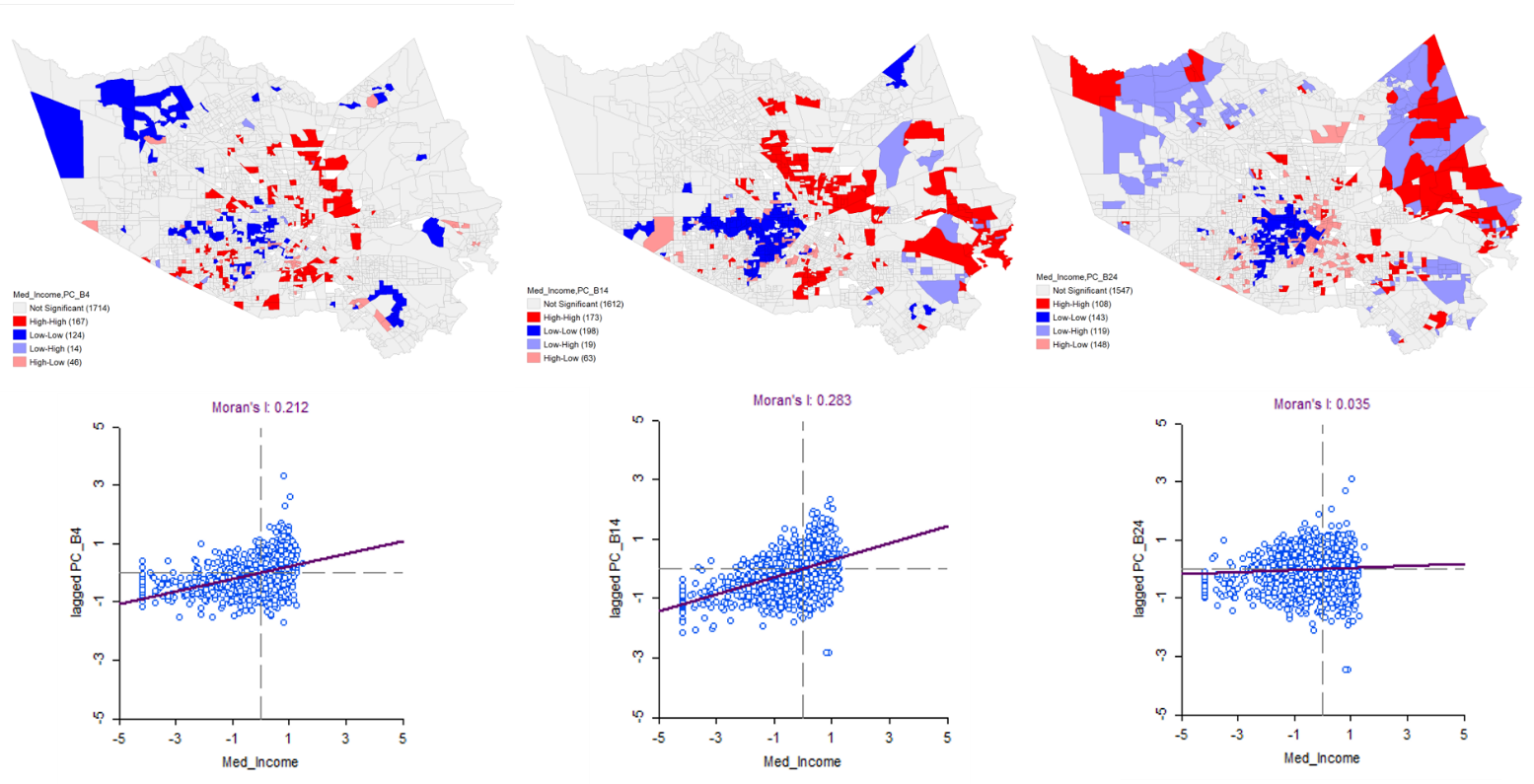


**b)**

**Figure E3a.** Spatial clusters of median income and population activity fluctuations in Houston

**Figure E3b.** Spatial clusters of median income and POI-CBG network in Houston

**a)
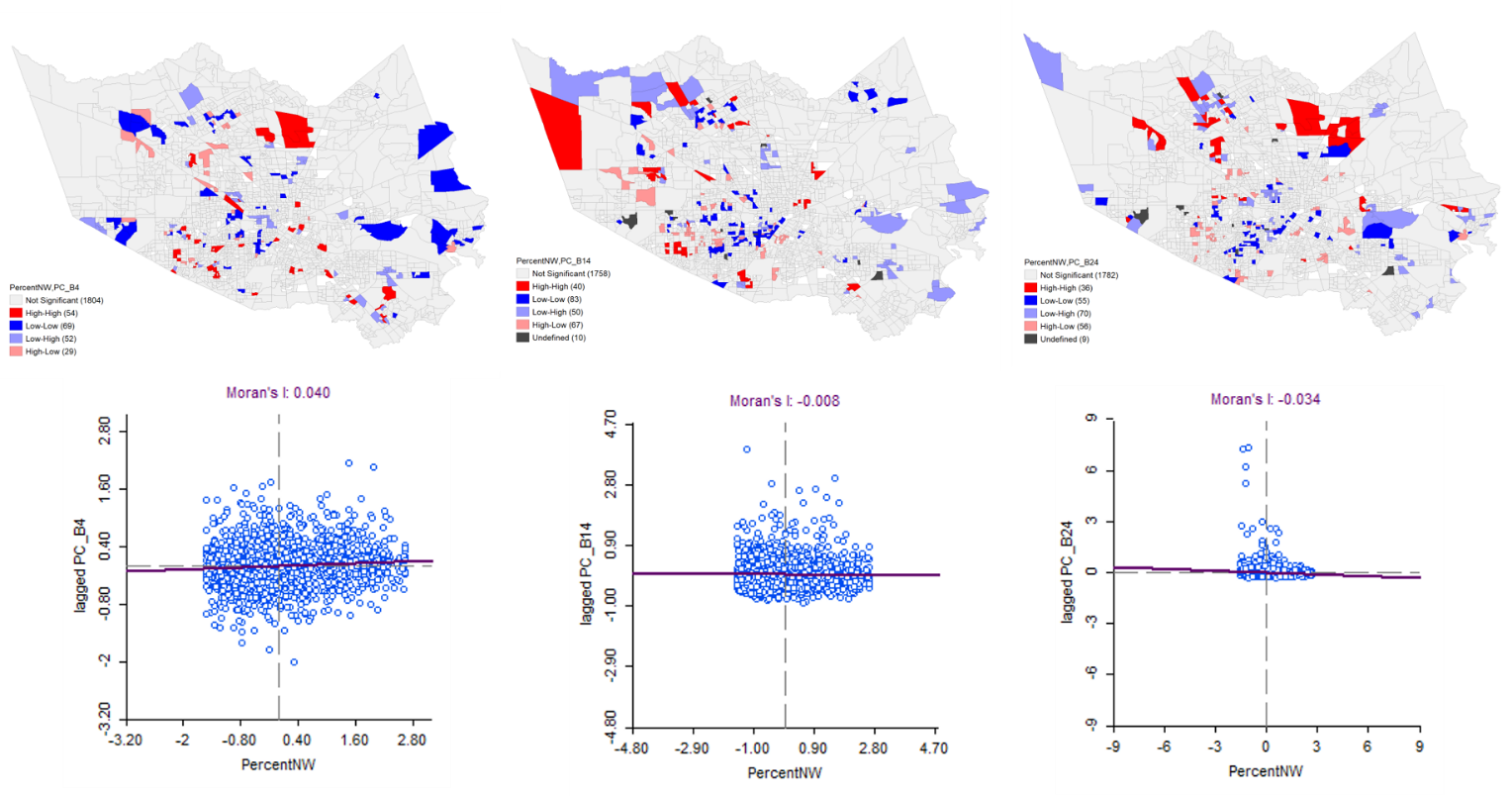
**
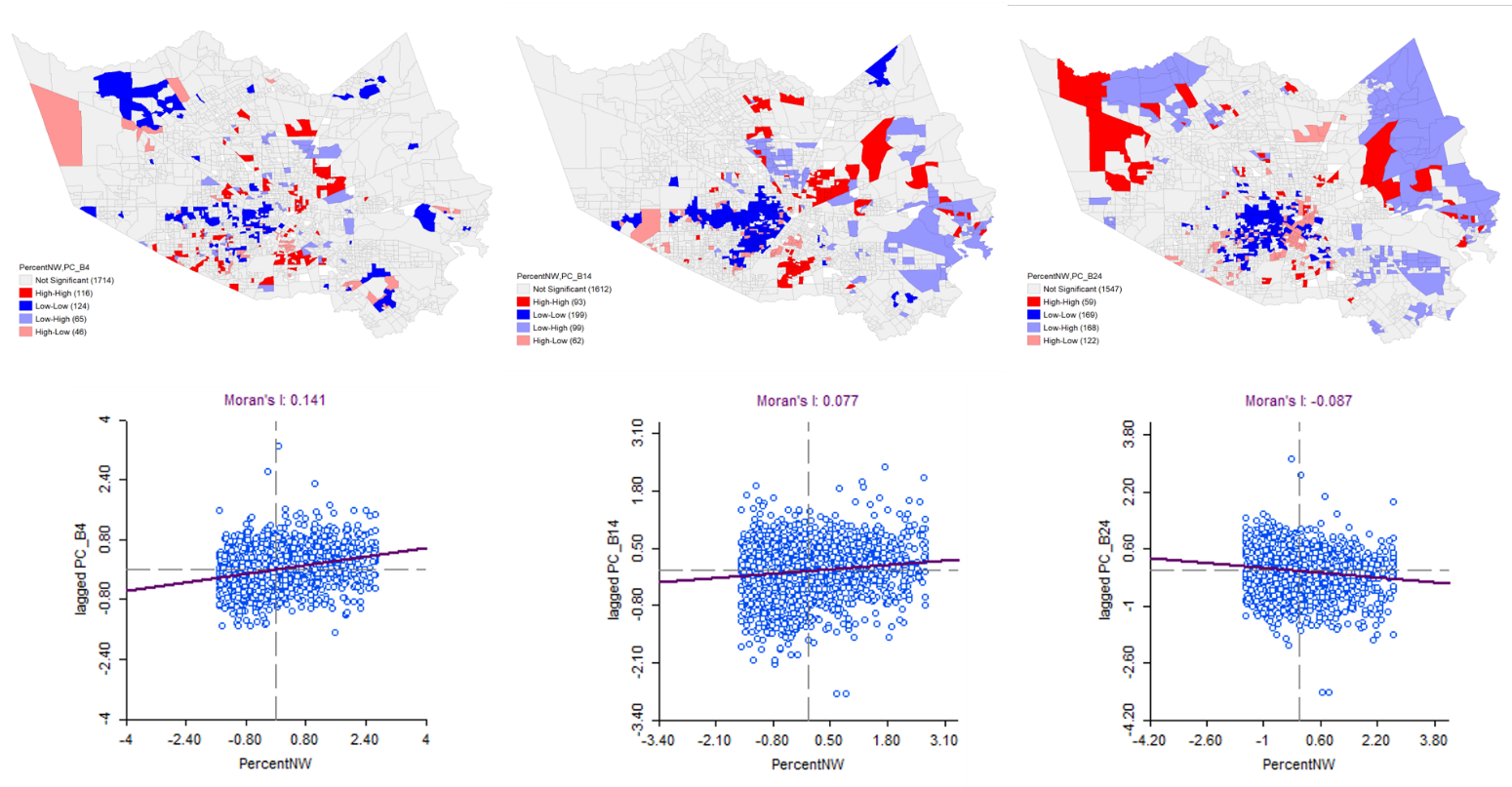


**b)**

**Figure E4a.** Spatial clusters of non-white populations and population activity fluctuations in Houston

**Figure E4b.** Spatial clusters of non-white populations and POI-CBG network in Houston

**a)
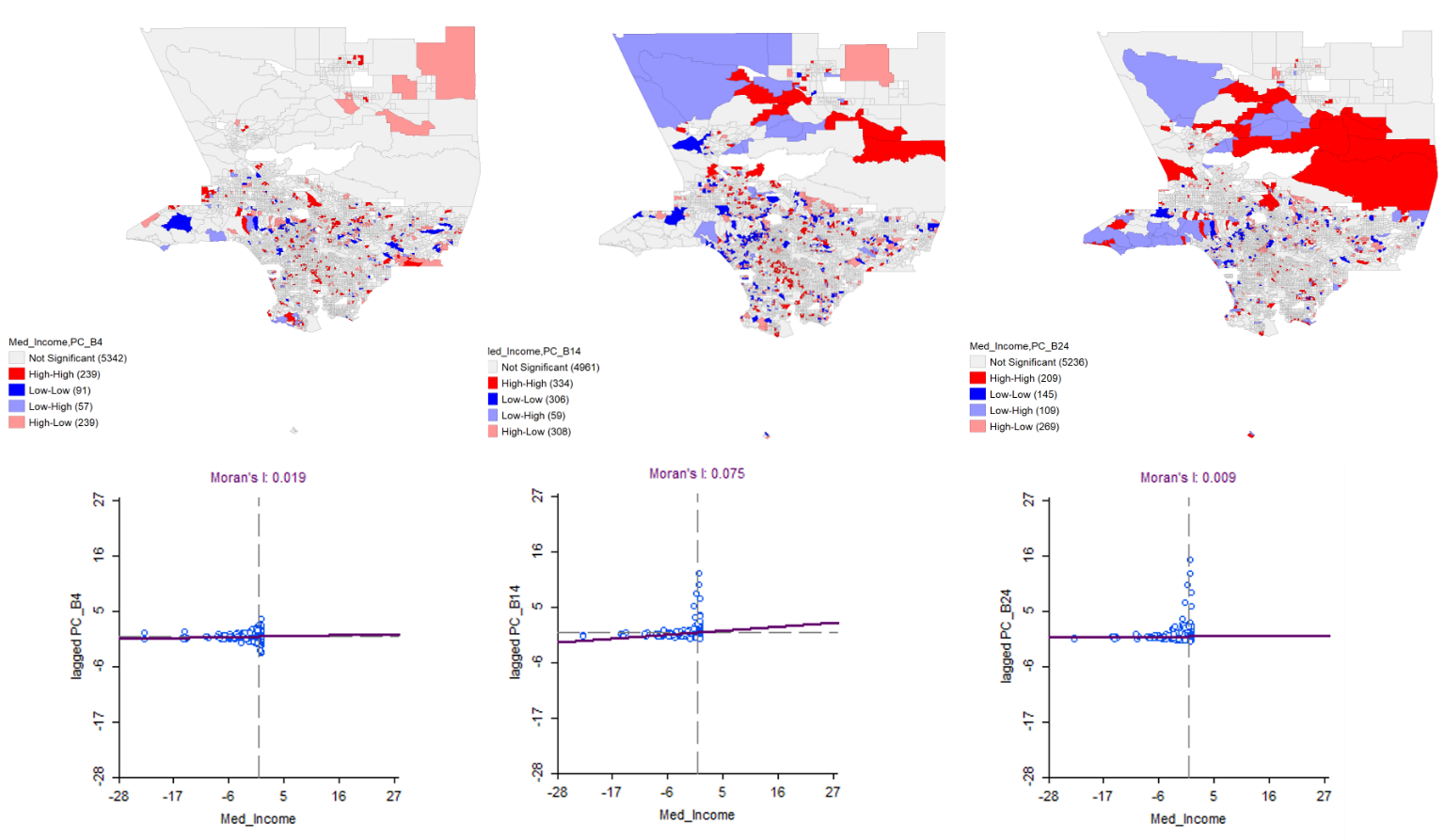
**
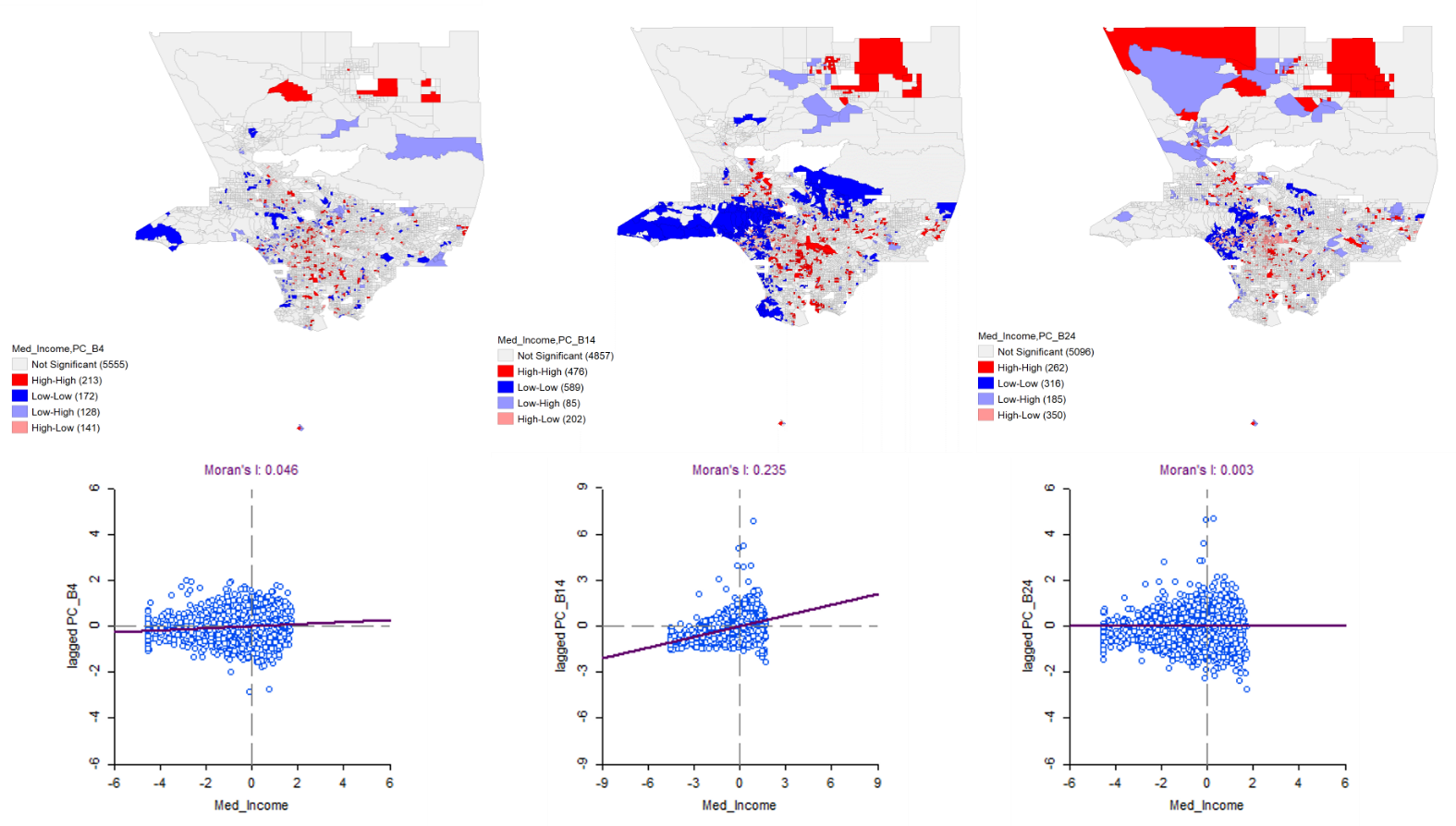


**b)**

**Figure E5a.** Spatial clusters of median income and population activity fluctuations in Los Angeles

**Figure E5b.** Spatial clusters of median income and POI-CBG network in Los Angeles

**a)
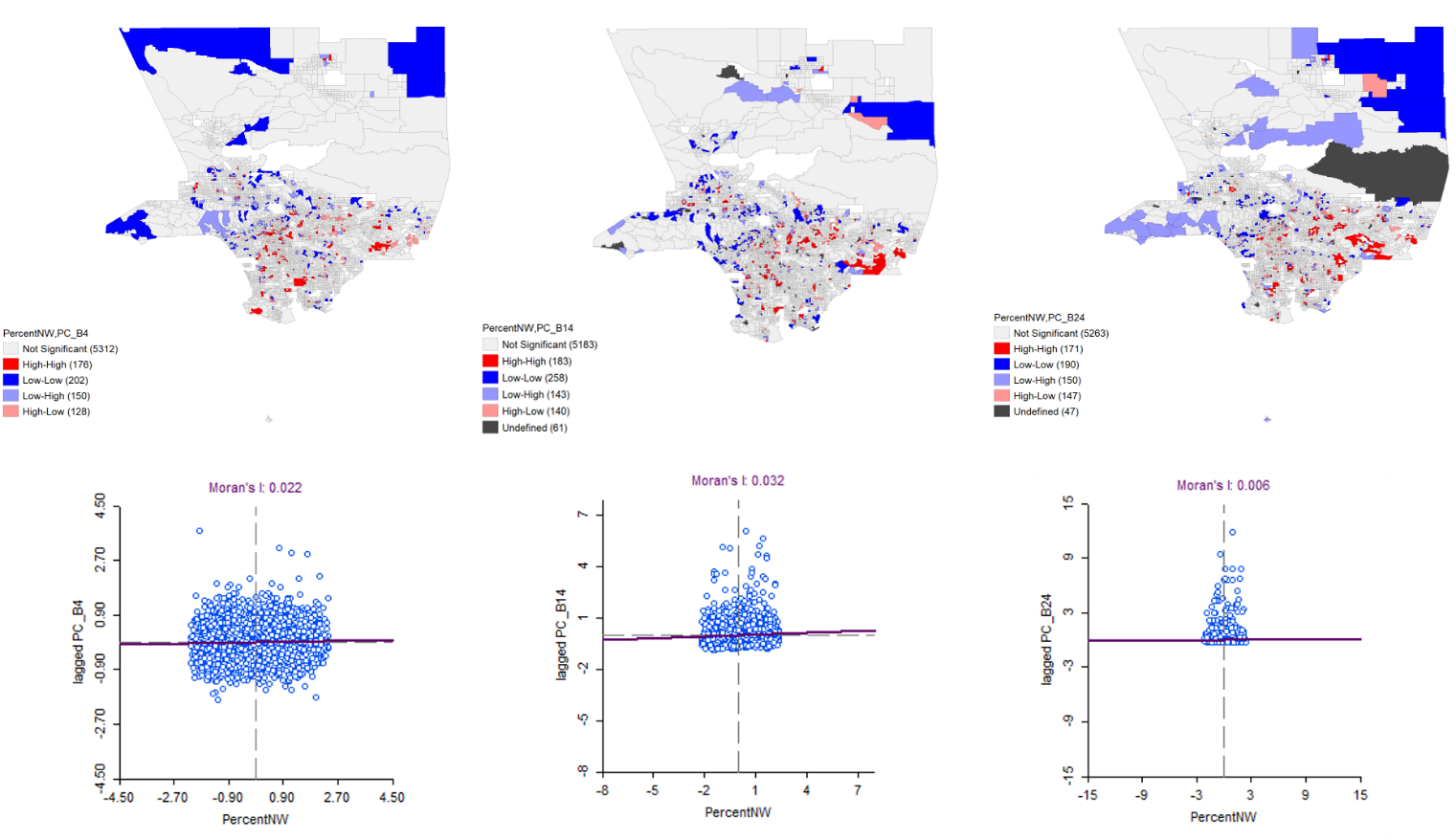

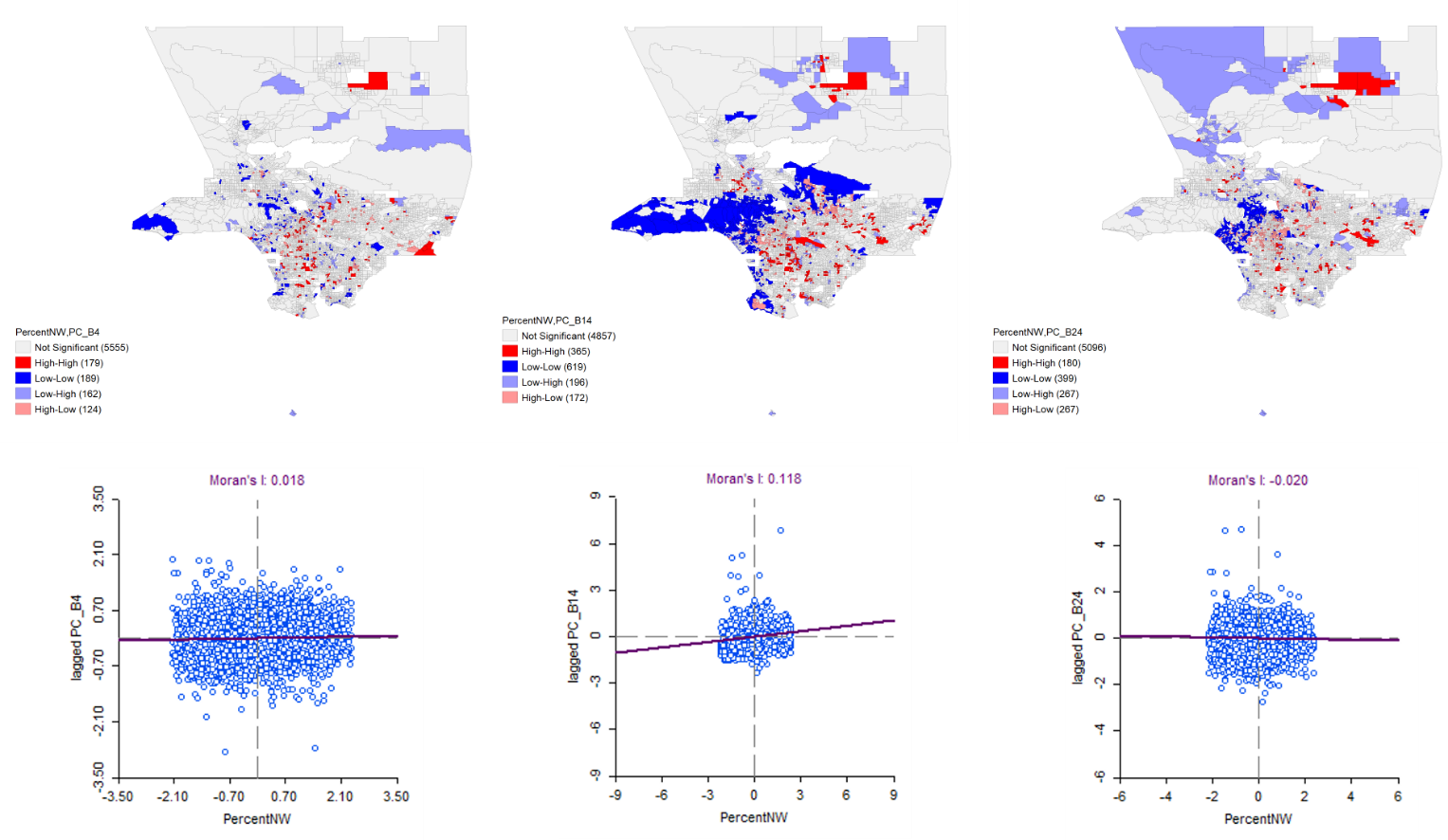
**

**b)**

**Figure E6a.** Spatial clusters of non-white populations and population activity fluctuations Los Angeles

**Figure E6b.** Spatial clusters of non-white populations and POI-CBG network in Los Angeles

**a)
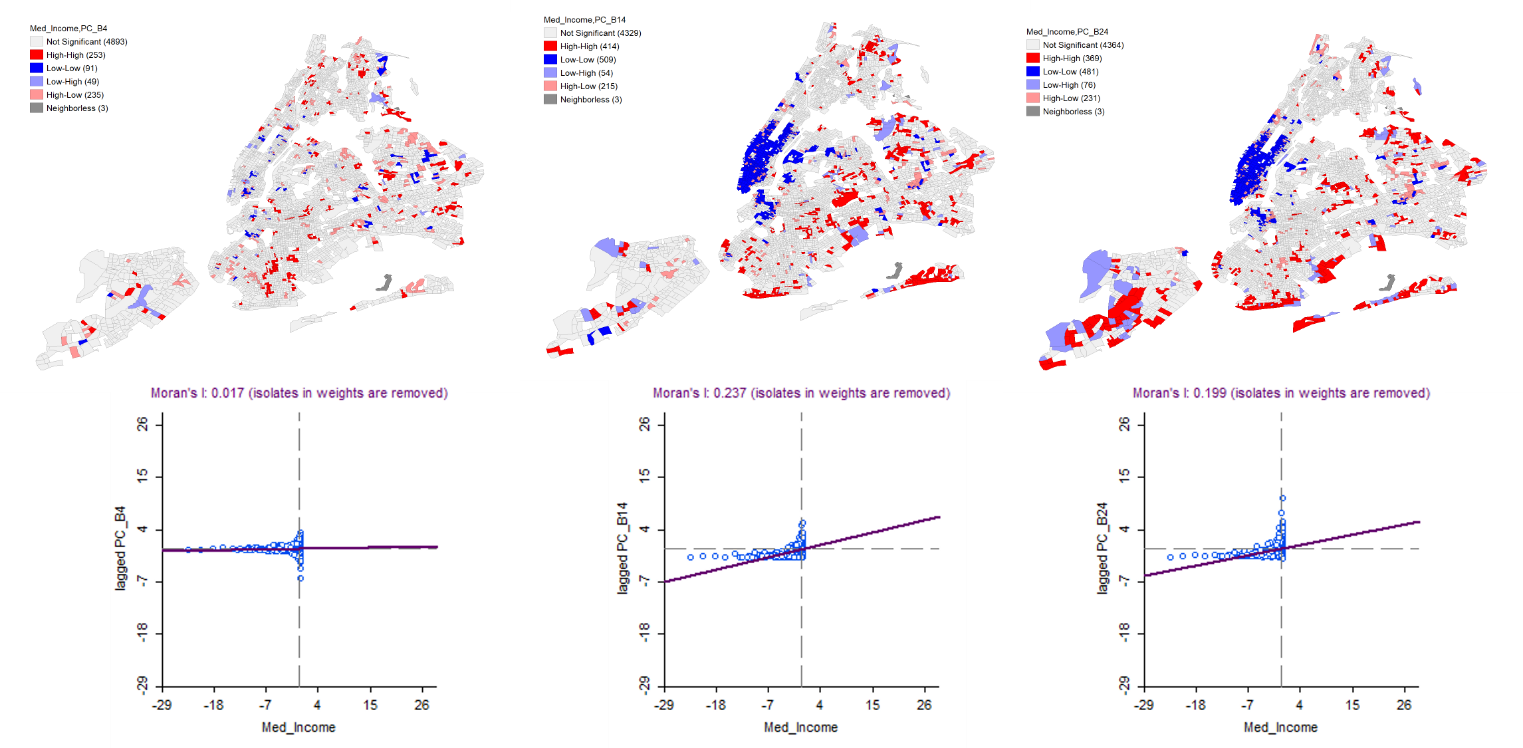
**
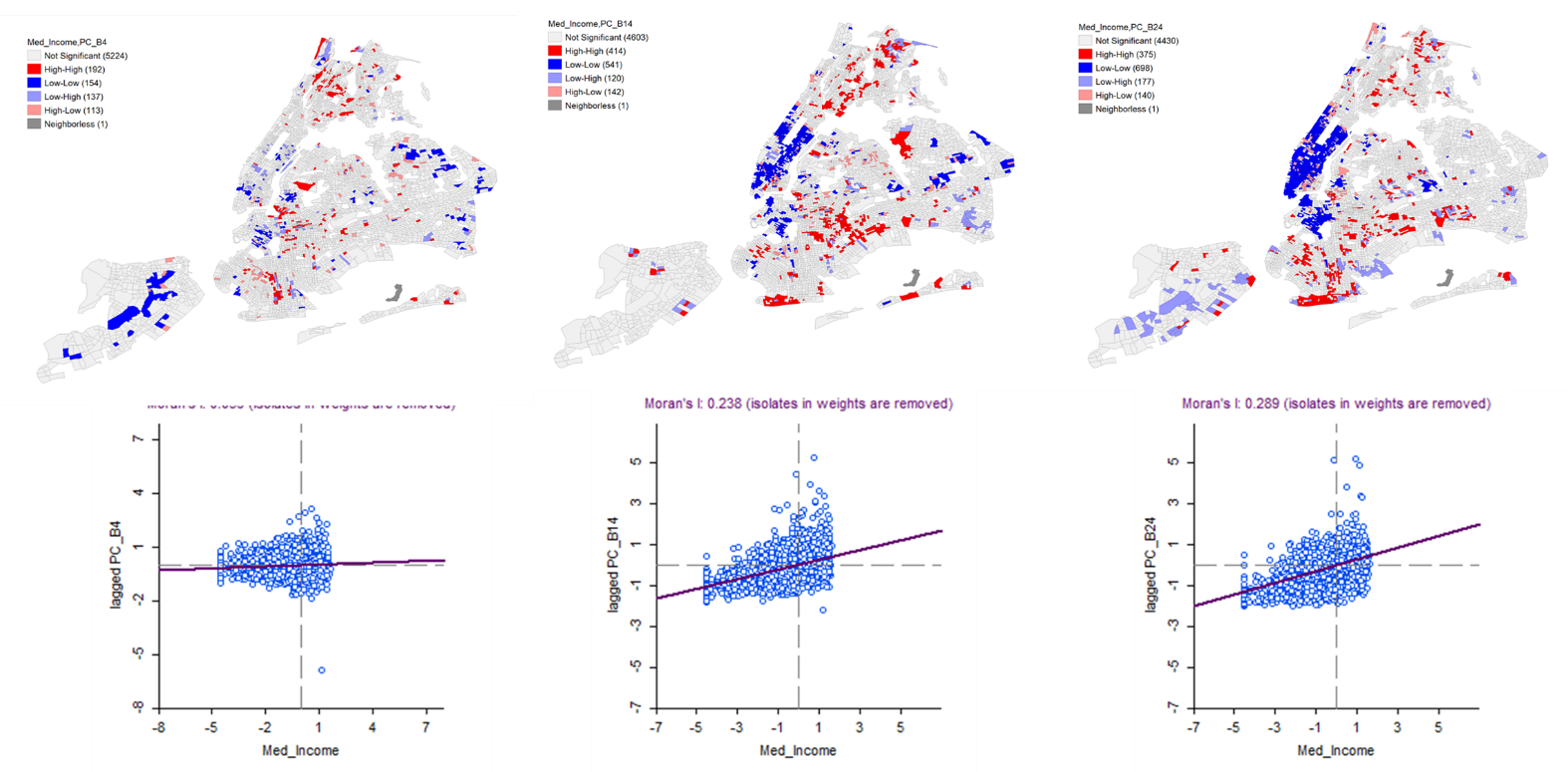


**b)**

**Figure E7a.** Spatial clusters of median income and population activity fluctuations in New York

**Figure E7b.** Spatial clusters of median income and POI-CBG network in New York

**a)**
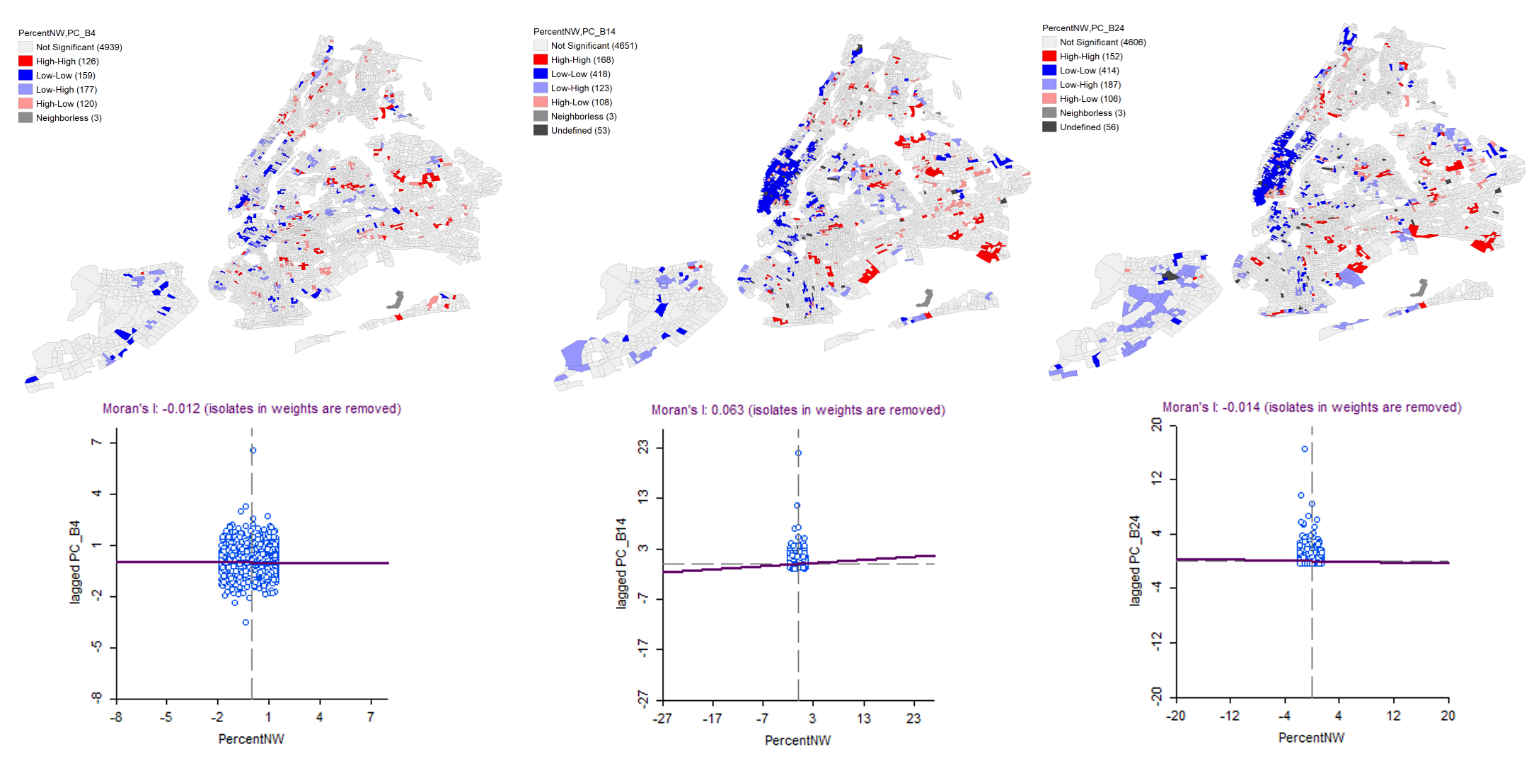
**
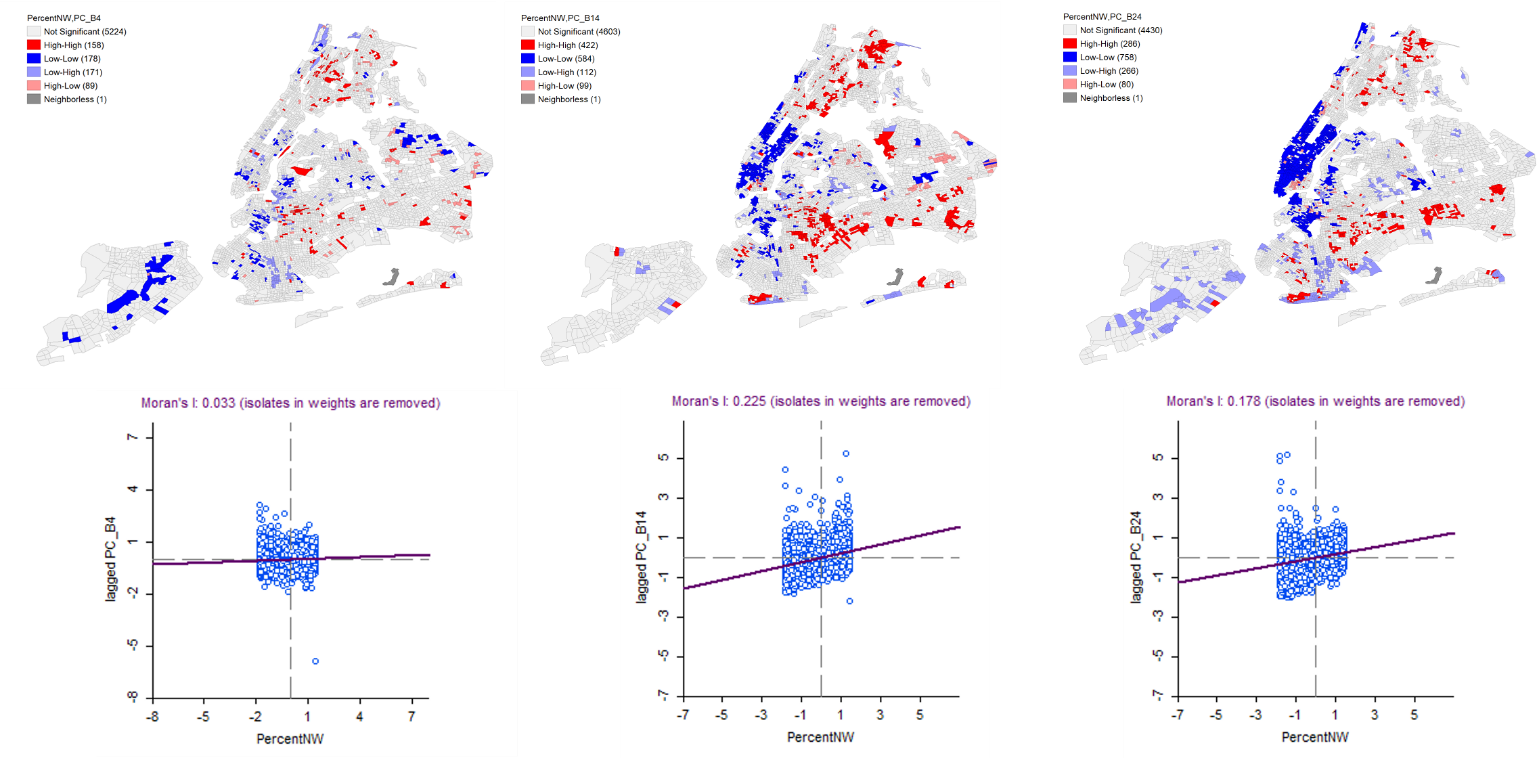
**

**b)**

**Figure E8a.** Spatial clusters of non-white populations and population activity fluctuations in New York

**Figure E8b.** Spatial clusters of non-white populations and POI-CBG network in New York

**a)
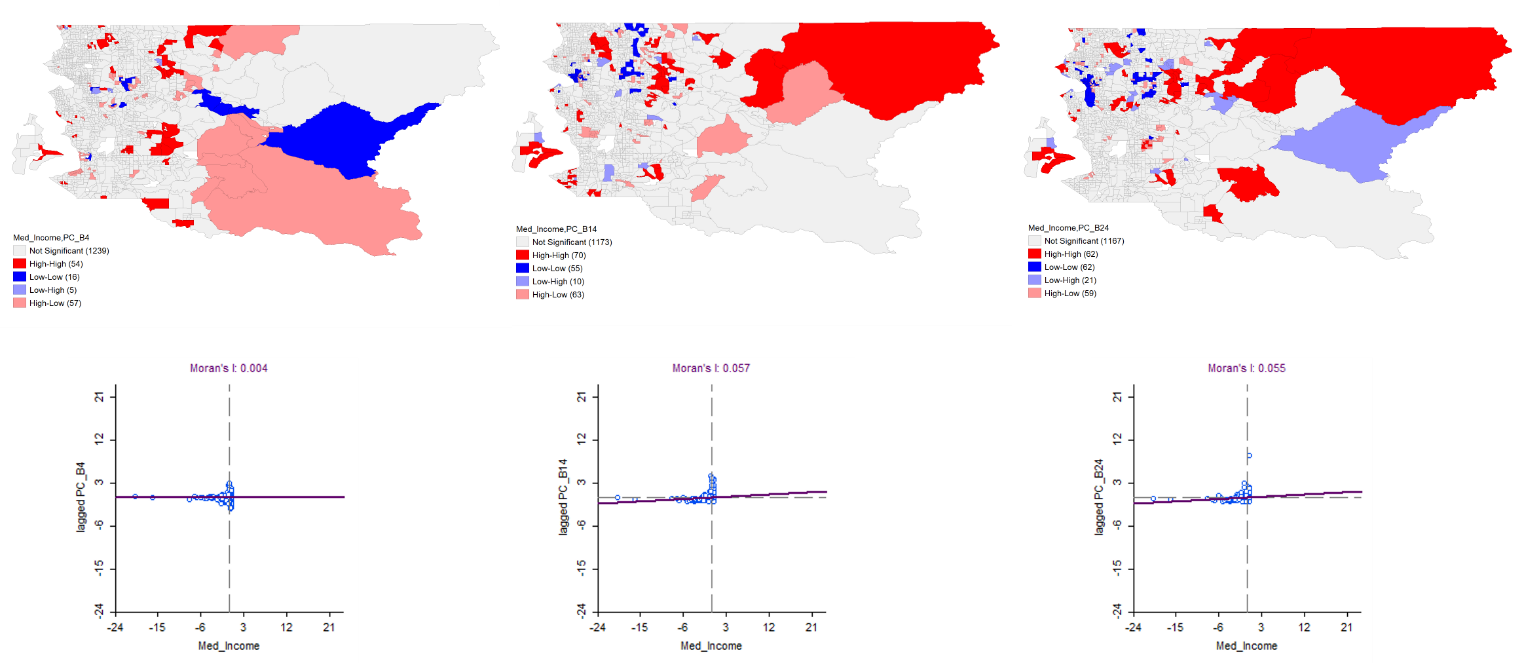

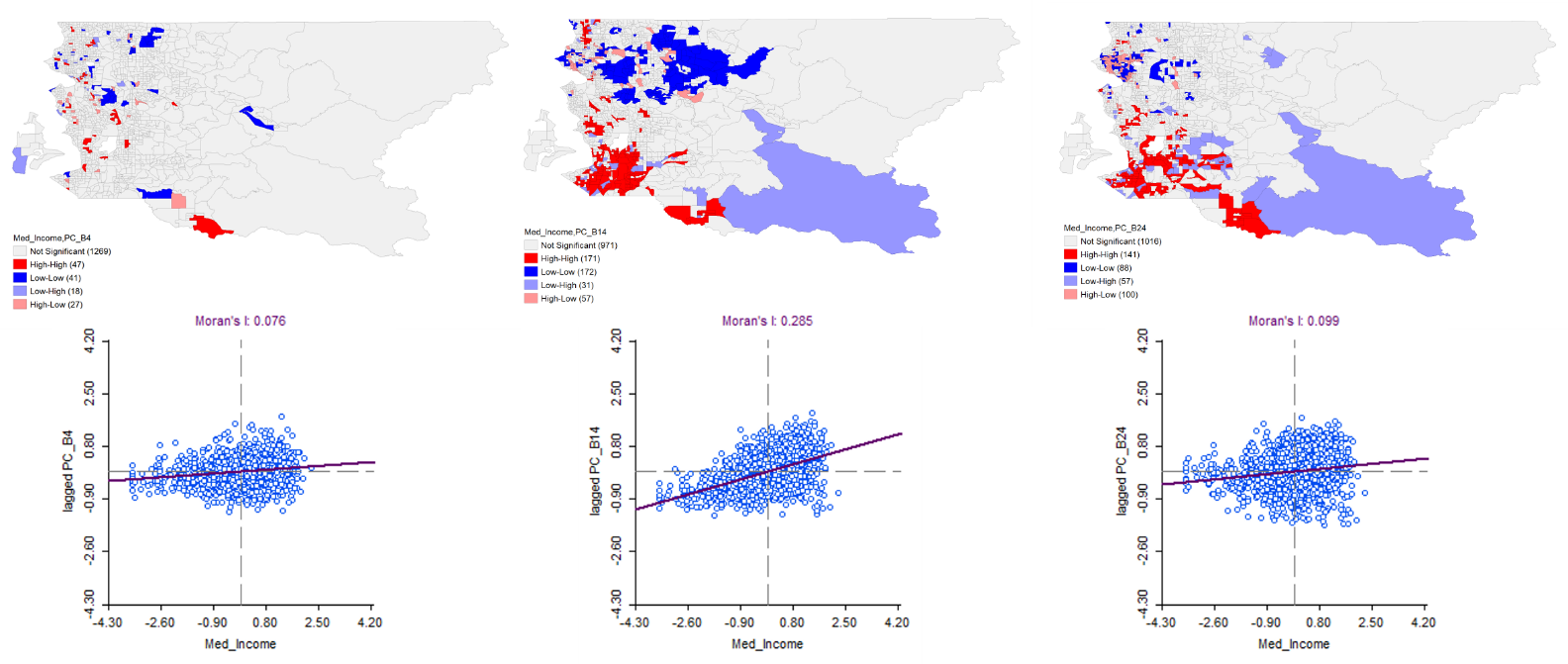
**

**b)**

**Figure E9a.** Spatial clusters of median income and population activity fluctuations in Seattle

**Figure E9b.** Spatial clusters of median income and POI-CBG network in Seattle

**a)
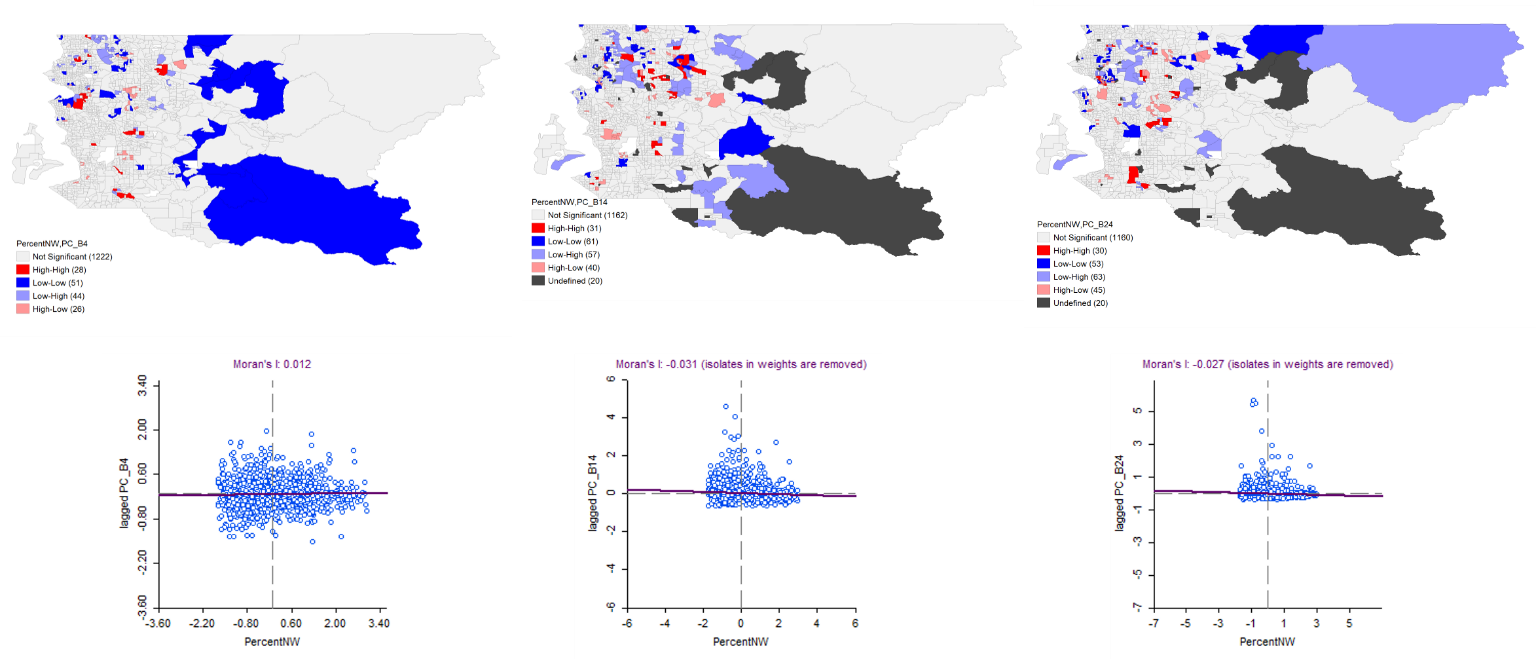

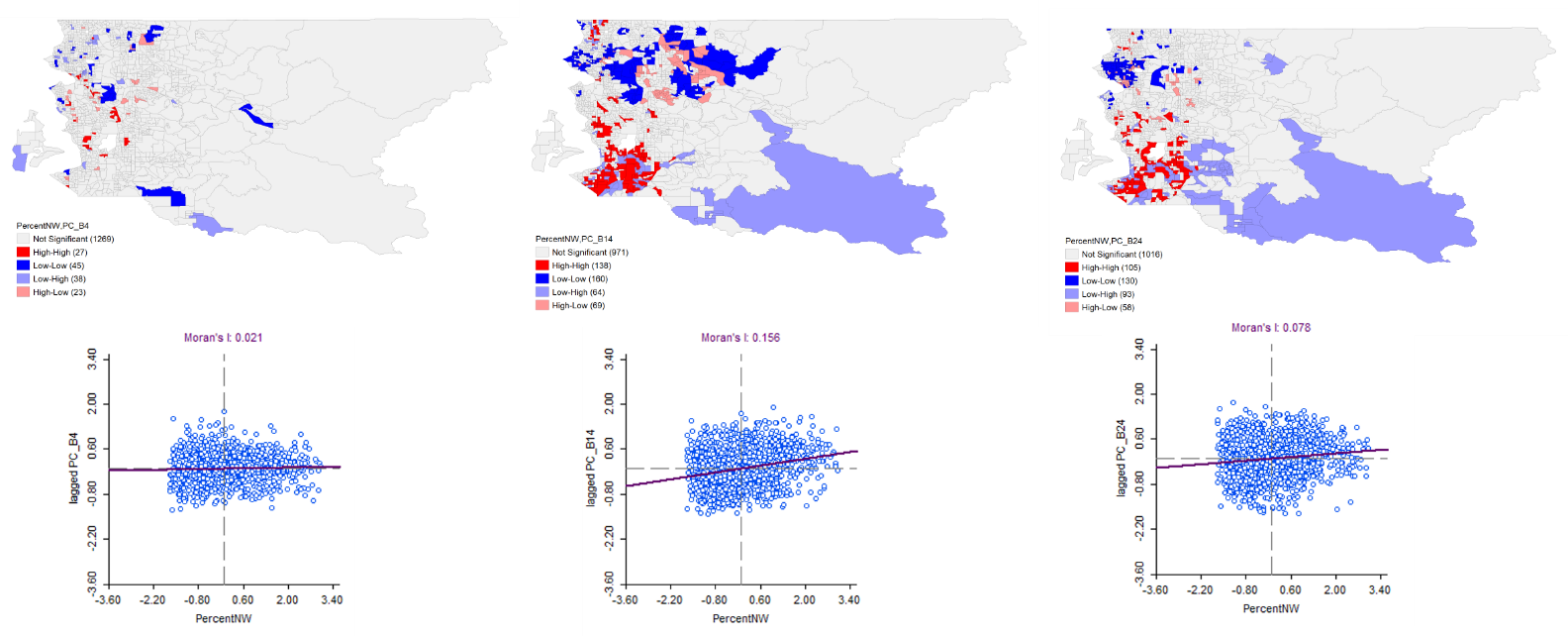
**

**b)**

**Figure E10a.** Spatial clusters of non-white populations and population activity fluctuations in Seattle

**Figure E10b.** Spatial clusters of non-white populations and POI-CBG network in Seattle

| Table E1. Spatial Clustering of Median Income and Population Activity Fluctuations | | | | | | | | | | | | | | | |
| --- | --- | --- | --- | --- | --- | --- | --- | --- | --- | --- | --- | --- | --- | --- | --- |
|  | Jan 27^th^,2020 to Feb 2^nd^, 2020 | | | | | April 6^th^ , 2020 to April 12^th^, 2020 | | | | | June 15^th^ , 2020 to June 21^st^, 2020 | | | | |
|  | Moran’s I | H-H | L-L | H-L | L-H | Moran’s I | H-H | L-L | H-L | L-H | Moran’s I | H-H | L-L | H-L | L-H |
| **Chicago** | .037 | 147 | 85 | 117 | 41 | .140 | 222 | 259 | 169 | 33 | .050 | 137 | 169 | 165 | 85 |
| **Houston** | .120 | 113 | 74 | 75 | 4 | .141 | 125 | 138 | 112 | 19 | .018 | 95 | 75 | 99 | 44 |
| **Los Angeles** | .019 | 239 | 91 | 239 | 57 | .075 | 334 | 306 | 308 | 59 | .009 | 209 | 145 | 269 | 109 |
| **New York** | .017 | 253 | 91 | 235 | 49 | .237 | 414 | 509 | 215 | 54 | .199 | 369 | 481 | 231 | 76 |
| **Seattle** | .004 | 54 | 18 | 57 | 5 | .057 | 70 | 55 | 63 | 10 | .055 | 62 | 62 | 59 | 21 |

| Table E2. Spatial Clustering of Median Income and POI-CBG Networks | | | | | | | | | | | | | | | |
| --- | --- | --- | --- | --- | --- | --- | --- | --- | --- | --- | --- | --- | --- | --- | --- |
|  | Jan 27^th^,2020 to Feb 2^nd^, 2020 | | | | | April 6^th^ , 2020 to April 12^th^, 2020 | | | | | June 15^th^ , 2020 to June 21^st^, 2020 | | | | |
|  | Moran’s I | H-H | L-L | H-L | L-H | Moran’s I | H-H | L-L | H-L | L-H | Moran’s I | H-H | L-L | H-L | L-H |
| **Chicago** | .091 | 176 | 109 | 91 | 59 | .343 | 478 | 469 | 139 | 54 | .132 | 234 | 347 | 181 | 112 |
| **Houston** | .212 | 167 | 124 | 46 | 14 | .283 | 173 | 198 | 63 | 19 | .035 | 108 | 143 | 148 | 119 |
| **Los Angeles** | .046 | 213 | 172 | 141 | 128 | .235 | 476 | 589 | 202 | 85 | .003 | 262 | 316 | 350 | 185 |
| **New York** | .039 | 192 | 154 | 113 | 137 | .238 | 414 | 541 | 142 | 120 | .289 | 375 | 698 | 140 | 177 |
| **Seattle** | .076 | 47 | 41 | 27 | 18 | .285 | 171 | 172 | 57 | 31 | .099 | 141 | 88 | 100 | 57 |

| Table E3. Spatial Clustering of Percentage of Non-white Populations and Population Activity Fluctuations | | | | | | | | | | | | | | | | |
| --- | --- | --- | --- | --- | --- | --- | --- | --- | --- | --- | --- | --- | --- | --- | --- | --- |
|  | Jan 27^th^,2020 to Feb 2^nd^, 2020 | | | | | April 6^th^ , 2020 to April 12^th^, 2020 | | | | | June 15^th^ , 2020 to June 21^st^, 2020 | | | | | |
|  | Moran’s I | H-H | L-L | H-L | L-H | Moran’s I | H-H | L-L | H-L | L-H | Moran’s I | H-H | L-L | H-L | L-H |  |
| **Chicago** | .061 | 79 | 151 | 43 | 91 | .043 | 78 | 264 | 52 | 124 | -.001 | 84 | 178 | 66 | 130 |  |
| **Houston** | .040 | 54 | 69 | 29 | 52 | -.008 | 40 | 83 | 67 | 50 | -.034 | 36 | 55 | 56 | 70 |  |
| **Los Angeles** | .022 | 176 | 202 | 128 | 150 | .032 | 183 | 258 | 140 | 143 | .006 | 171 | 190 | 150 | 147 |  |
| **New York** | -.012 | 126 | 159 | 120 | 177 | .063 | 168 | 418 | 108 | 123 | -.014 | 152 | 414 | 106 | 187 |  |
| **Seattle** | .012 | 28 | 51 | 26 | 44 | -.031 | 31 | 61 | 40 | 57 | -.027 | 30 | 53 | 45 | 63 |  |

| Table E4. Spatial Clustering of Percentage of Non-white Populations and POI-CBG Networks | | | | | | | | | | | | | | | |
| --- | --- | --- | --- | --- | --- | --- | --- | --- | --- | --- | --- | --- | --- | --- | --- |
|  | Jan 27^th^,2020 to Feb 2^nd^, 2020 | | | | | April 6^th^ , 2020 to April 12^th^, 2020 | | | | | June 15^th^ , 2020 to June 21^st^, 2020 | | | | |
|  | Moran’s I | H-H | L-L | H-L | L-H | Moran’s I | H-H | L-L | H-L | L-H | Moran’s I | H-H | L-L | H-L | L-H |
| **Chicago** | .126 | 150 | 156 | 44 | 85 | .341 | 445 | 547 | 61 | 87 | .125 | 186 | 428 | 100 | 160 |
| **Houston** | .141 | 116 | 124 | 46 | 65 | .077 | 93 | 199 | 99 | 62 | -.087 | 59 | 169 | 122 | 168 |
| **Los Angeles** | .018 | 179 | 189 | 124 | 162 | .118 | 365 | 619 | 172 | 196 | -.200 | 180 | 399 | 267 | 267 |
| **New York** | .033 | 158 | 178 | 89 | 171 | .225 | 422 | 584 | 99 | 112 | .178 | 286 | 758 | 80 | 266 |
| **Seattle** | .021 | 27 | 45 | 23 | 38 | .156 | 138 | 160 | 69 | 64 | .078 | 105 | 130 | 58 | 93 |

## **Percent Difference to In Degree Values**

The tables show the percent difference of the In Degree values. It compares each income group and each racial-ethnic group with one another before the implementation of NPIs (Feb 3^rd^ – Feb 8^th^ and March 2^nd^ – March 7^th^) and after the implementation of NPIs (March 30^th^- April 5^th^, April 6^th^- April 12^th^, May 4^th^- May 10^th^). The title of the subtables indicates which variable is being compared, or “Percent Difference to X”. If the value is positive, it signals that X has a lower In Degree value, and thus, a higher comparative exposure risk. If the value is negative, it signals that X has a higher In Degree value, and thus, a lower comparative exposure risk.

Table F1. Percent difference values for in degree values among different income groups

| April 6^th^, 2020 - April 12^th^, 2020 | | | | | | |
| --- | --- | --- | --- | --- | --- | --- |
|  | <$20,000 | $20,000-$49,999 | $50,000-$99,999 | $100,00-$150,000 | $150,00-$200,000 | > $200k |
| Chicago | 0.61 | 0.59 | 0.57 | 0.54 | 0.52 | 0.49 |
| Houston | 0.61 | 0.59 | 0.57 | 0.54 | 0.52 | 0.49 |
| Los Angeles | 0.53 | 0.53 | 0.51 | 0.48 | 0.46 | 0.42 |
| New York | 0.55 | 0.53 | 0.51 | 0.50 | 0.49 | 0.46 |
| Seattle | 0.61 | 0.60 | 0.57 | 0.54 | 0.52 | 0.49 |
| Percent Difference to <$20,000 | | | | | |  |
| Chicago | 0.00 | 3.17 | 5.95 | 10.71 | 14.37 | 19.49 |
| Houston | 0.00 | 3.13 | 5.92 | 10.63 | 14.36 | 19.40 |
| Los Angeles | 0.00 | 0.88 | 4.69 | 9.33 | 13.45 | 20.95 |
| New York | 0.00 | 3.51 | 6.60 | 9.58 | 11.96 | 17.38 |
| Seattle | 0.00 | 3.15 | 6.57 | 12.01 | 15.67 | 19.73 |
| Percent Difference to $20,000-$49,999 | | | | | |  |
| Chicago | -3.28 | 0.00 | 2.87 | 7.78 | 11.57 | 16.86 |
| Houston | -3.23 | 0.00 | 2.88 | 7.75 | 11.59 | 16.80 |
| Los Angeles | -0.89 | 0.00 | 3.84 | 8.52 | 12.68 | 20.25 |
| New York | -3.64 | 0.00 | 3.21 | 6.29 | 8.76 | 14.37 |
| Seattle | -3.25 | 0.00 | 3.53 | 9.15 | 12.92 | 17.12 |
| Percent Difference to $50,000-$99,999 | | | | | |  |
| Chicago | -6.33 | -2.95 | 0.00 | 5.06 | 8.95 | 14.40 |
| Houston | -6.29 | -2.97 | 0.00 | 5.01 | 8.97 | 14.33 |
| Los Angeles | -4.92 | -3.99 | 0.00 | 4.86 | 9.19 | 17.06 |
| New York | -7.07 | -3.31 | 0.00 | 3.19 | 5.74 | 11.53 |
| Seattle | -7.03 | -3.66 | 0.00 | 5.82 | 9.74 | 14.09 |
| Percent Difference to $100,000-$150,000 | | | | | |  |
| Chicago | -11.99 | -8.44 | -5.33 | 0.00 | 4.10 | 9.84 |
| Houston | -11.90 | -8.40 | -5.27 | 0.00 | 4.17 | 9.81 |
| Los Angeles | -10.29 | -9.31 | -5.11 | 0.00 | 4.54 | 12.82 |
| New York | -10.60 | -6.72 | -3.30 | 0.00 | 2.63 | 8.62 |
| Seattle | -13.65 | -10.07 | -6.18 | 0.00 | 4.16 | 8.78 |
| Percent Difference to >$150,000 - $200,000 | | |  |  |  |  |
| Chicago | -16.78 | -13.08 | -9.83 | -4.28 | 0.00 | 5.98 |
| Houston | -16.77 | -13.12 | -9.85 | -4.35 | 0.00 | 5.89 |
| Los Angeles | -15.54 | -14.52 | -10.12 | -4.76 | 0.00 | 8.67 |
| New York | -13.59 | -9.60 | -6.09 | -2.70 | 0.00 | 6.15 |
| Seattle | -18.58 | -14.84 | -10.79 | -4.34 | 0.00 | 4.82 |
| Percent Difference to $>200k | |  |  |  |  |  |
| Chicago | -24.21 | -20.27 | -16.82 | -10.91 | -6.36 | 0.00 |
| Houston | -24.08 | -20.19 | -16.73 | -10.88 | -6.26 | 0.00 |
| Los Angeles | -26.51 | -25.39 | -20.57 | -14.71 | -9.49 | 0.00 |
| New York | -21.03 | -16.78 | -13.04 | -9.43 | -6.55 | 0.00 |
| Seattle | -24.58 | -20.66 | -16.40 | -9.62 | -5.07 | 0.00 |

| May 4^th^, 2020 - May 10^th^, 2020 | | | | | | |
| --- | --- | --- | --- | --- | --- | --- |
|  | <$20,000 | $20,000-$49,999 | $50,000-$99,999 | $100,00-$150,000 | $150,00-$200,000 | > $200k |
| Chicago | 0.80 | 0.78 | 0.75 | 0.72 | 0.69 | 0.65 |
| Houston | 0.80 | 0.77 | 0.75 | 0.72 | 0.69 | 0.65 |
| Los Angeles | 0.80 | 0.79 | 0.76 | 0.72 | 0.69 | 0.64 |
| New York | 0.76 | 0.74 | 0.71 | 0.68 | 0.66 | 0.62 |
| Seattle | 0.85 | 0.81 | 0.79 | 0.74 | 0.71 | 0.69 |
| Percent Difference to <$20,000 | | | | | |  |
| Chicago | 0.00 | 3.21 | 6.14 | 10.64 | 14.09 | 18.70 |
| Houston | 0.00 | 3.18 | 6.08 | 10.52 | 14.02 | 18.54 |
| Los Angeles | 0.00 | 0.90 | 4.96 | 9.82 | 14.07 | 20.48 |
| New York | 0.00 | 3.18 | 6.66 | 10.56 | 13.42 | 18.93 |
| Seattle | 0.00 | 4.20 | 7.54 | 12.69 | 16.33 | 18.98 |
| Percent Difference to $20,000-$49,999 | | | | | |  |
| Chicago | -3.32 | 0.00 | 3.03 | 7.68 | 11.25 | 16.00 |
| Houston | -3.28 | 0.00 | 2.99 | 7.58 | 11.20 | 15.86 |
| Los Angeles | -0.91 | 0.00 | 4.10 | 9.00 | 13.29 | 19.75 |
| New York | -3.28 | 0.00 | 3.60 | 7.62 | 10.58 | 16.27 |
| Seattle | -4.38 | 0.00 | 3.49 | 8.86 | 12.67 | 15.42 |
| Percent Difference to $50,000-$99,999 | | | | | |  |
| Chicago | -6.54 | -3.12 | 0.00 | 4.80 | 8.47 | 13.38 |
| Houston | -6.47 | -3.09 | 0.00 | 4.73 | 8.46 | 13.26 |
| Los Angeles | -5.22 | -4.27 | 0.00 | 5.11 | 9.58 | 16.32 |
| New York | -7.14 | -3.73 | 0.00 | 4.17 | 7.24 | 13.15 |
| Seattle | -8.15 | -3.61 | 0.00 | 5.57 | 9.51 | 12.37 |
| Percent Difference to $100,000-$150,000 | | | | | |  |
| Chicago | -11.91 | -8.32 | -5.04 | 0.00 | 3.86 | 9.01 |
| Houston | -11.75 | -8.20 | -4.96 | 0.00 | 3.92 | 8.96 |
| Los Angeles | -10.89 | -9.89 | -5.38 | 0.00 | 4.71 | 11.82 |
| New York | -11.80 | -8.25 | -4.36 | 0.00 | 3.20 | 9.37 |
| Seattle | -14.53 | -9.72 | -5.90 | 0.00 | 4.17 | 7.20 |
| Percent Difference to >$150,000 - $200,000 | | |  |  |  |  |
| Chicago | -16.41 | -12.67 | -9.26 | -4.02 | 0.00 | 5.36 |
| Houston | -16.31 | -12.61 | -9.24 | -4.08 | 0.00 | 5.25 |
| Los Angeles | -16.37 | -15.32 | -10.60 | -4.95 | 0.00 | 7.45 |
| New York | -15.50 | -11.83 | -7.81 | -3.31 | 0.00 | 6.37 |
| Seattle | -19.52 | -14.50 | -10.51 | -4.36 | 0.00 | 3.16 |
| Percent Difference to $>200k | |  |  |  |  |  |
| Chicago | -23.00 | -19.05 | -15.44 | -9.91 | -5.66 | 0.00 |
| Houston | -22.75 | -18.85 | -15.29 | -9.84 | -5.54 | 0.00 |
| Los Angeles | -25.75 | -24.61 | -19.50 | -13.40 | -8.05 | 0.00 |
| New York | -23.36 | -19.44 | -15.14 | -10.33 | -6.80 | 0.00 |
| Seattle | -23.42 | -18.24 | -14.11 | -7.76 | -3.26 | 0.00 |

Table F2. Percent difference values for in degree values among different racial-ethnic groups

| Feb 3^rd^, 2020 - Feb 8^th^, 2020 | | | | | | | | | | | | |  |
| --- | --- | --- | --- | --- | --- | --- | --- | --- | --- | --- | --- | --- | --- |
|  | | White | Black or African American | | American Indian or Alaska Native | | Asian | | Native Hawaiian/ Other Pacific Islander | | Hispanic or Latino | |  |
| Chicago | | 1.03 | 1.03 | | 1.01 | | 1.03 | | 1.01 | | 1.01 | |  |
| Houston | | 1.02 | 1.02 | | 1.01 | | 1.02 | | 1.01 | | 1.01 | |  |
| Los Angeles | | 1.04 | 1.04 | | 1.02 | | 1.04 | | 1.01 | | 1.01 | |  |
| New York | | 1.05 | 1.06 | | 1.02 | | 1.06 | | 1.01 | | 1.02 | |  |
| Seattle | | 1.05 | 1.05 | | 1.03 | | 1.05 | | 1.03 | | 1.02 | |  |
| Percent Difference to White | | | | | | | | | | | | |  |
| Chicago | | 0.00 | -0.46 | | 1.38 | | 0.11 | | 1.67 | | 1.67 | |  |
| Houston | | 0.00 | 0.21 | | 0.96 | | 0.25 | | 1.00 | | 0.90 | |  |
| Los Angeles | | 0.00 | 0.33 | | 2.14 | | -0.10 | | 2.61 | | 2.82 | |  |
| New York | | 0.00 | -1.28 | | 2.55 | | -0.88 | | 3.71 | | 3.28 | |  |
| Seattle | | 0.00 | 0.47 | | 2.03 | | -0.16 | | 2.16 | | 3.38 | |  |
| Percent Difference to Black or African American | | | | | | | | | | | | |  |
| Chicago | | 0.46 | 0.00 | | 1.84 | | 0.58 | | 2.13 | | 2.13 | |  |
| Houston | | -0.21 | 0.00 | | 0.76 | | 0.05 | | 0.80 | | 0.70 | |  |
| Los Angeles | | -0.33 | 0.00 | | 1.82 | | -0.43 | | 2.29 | | 2.50 | |  |
| New York | | 1.27 | 0.00 | | 3.78 | | 0.40 | | 4.93 | | 4.50 | |  |
| Seattle | | -0.47 | 0.00 | | 1.57 | | -0.63 | | 1.70 | | 2.92 | |  |
| Percent Difference to American Indian or Alaska Native | | | | | | | | | | | | |  |
| Chicago | | -1.40 | -1.87 | | 0.00 | | -1.28 | | 0.30 | | 0.30 | |  |
| Houston | | -0.97 | -0.76 | | 0.00 | | -0.71 | | 0.04 | | -0.06 | |  |
| Los Angeles | | -2.19 | -1.85 | | 0.00 | | -2.29 | | 0.48 | | 0.69 | |  |
| New York | | -2.62 | -3.93 | | 0.00 | | -3.52 | | 1.19 | | 0.74 | |  |
| Seattle | | -2.08 | -1.60 | | 0.00 | | -2.24 | | 0.13 | | 1.37 | |  |
| Percent Difference to Asian | | | | | | | | | | | | |  |
| Chicago | | -0.12 | -0.58 | | 1.27 | | 0.00 | | 1.56 | | 1.56 | |  |
| Houston | | -0.26 | -0.05 | | 0.71 | | 0.00 | | 0.75 | | 0.65 | |  |
| Los Angeles | | 0.10 | 0.43 | | 2.24 | | 0.00 | | 2.71 | | 2.92 | |  |
| New York | | 0.87 | -0.40 | | 3.40 | | 0.00 | | 4.55 | | 4.12 | |  |
| Seattle | | 0.16 | 0.63 | | 2.19 | | 0.00 | | 2.32 | | 3.53 | |  |
| Percent Difference to Native Hawaiian/ Other Pacific Islander | | | | | | | | | | | | |  |
| Chicago | | -1.70 | -2.17 | | -0.30 | | -1.58 | | 0.00 | | 0.00 | |  |
| Houston | | -1.01 | -0.80 | | -0.04 | | -0.75 | | 0.00 | | -0.10 | |  |
| Los Angeles | | -2.68 | -2.35 | | -0.48 | | -2.79 | | 0.00 | | 0.21 | |  |
| New York | | -3.85 | -5.18 | | -1.20 | | -4.77 | | 0.00 | | -0.45 | |  |
| Seattle | | -2.21 | -1.73 | | -0.13 | | -2.37 | | 0.00 | | 1.25 | |  |
| Percent Difference to Hispanic or Latino | | | | |  | |  | |  | |  | |  |
| Chicago | | -1.70 | -2.17 | | -0.30 | | -1.58 | | 0.00 | | 0.00 | |  |
| Houston | | -0.91 | -0.70 | | 0.06 | | -0.66 | | 0.10 | | 0.00 | |  |
| Los Angeles | | -2.90 | -2.56 | | -0.70 | | -3.01 | | -0.21 | | 0.00 | |  |
| New York | | -3.39 | -4.71 | | -0.75 | | -4.30 | | 0.45 | | 0.00 | |  |
| Seattle | | -3.50 | -3.01 | | -1.39 | | -3.66 | | -1.26 | | 0.00 | |  |
| March 2^nd^, 2020 - March 7^th^, 2020 | | | | | | | | | | | | | |
|  | White | | | Black or African American | | American Indian or Alaska Native | | Asian | | Native Hawaiian/ Other Pacific Islander | | Hispanic or Latino | |
| Chicago | 1.24 | | | 1.20 | | 1.06 | | 1.22 | | 1.02 | | 1.02 | |
| Houston | 1.16 | | | 1.11 | | 1.03 | | 1.08 | | 1.01 | | 1.01 | |
| Los Angeles | 1.24 | | | 1.20 | | 1.08 | | 1.22 | | 1.04 | | 1.01 | |
| New York | 1.36 | | | 1.30 | | 1.10 | | 1.31 | | 1.02 | | 1.01 | |
| Seattle | 1.28 | | | 1.25 | | 1.14 | | 1.27 | | 1.11 | | 1.01 | |
| Percent Difference to White | | | | | | | | | | | | | |
| Chicago | 0.00 | | | 3.02 | | 14.33 | | 2.17 | | 18.27 | | 18.27 | |
| Houston | 0.00 | | | 3.50 | | 11.26 | | 6.10 | | 12.36 | | 12.69 | |
| Los Angeles | 0.00 | | | 3.23 | | 12.87 | | 1.30 | | 15.86 | | 18.51 | |
| New York | 0.00 | | | 4.17 | | 19.34 | | 3.46 | | 24.93 | | 25.51 | |
| Seattle | 0.00 | | | 2.46 | | 10.92 | | 0.84 | | 13.21 | | 21.41 | |
| Percent Difference to Black or African American | | | | | | | | | | | | | |
| Chicago | -3.11 | | | 0.00 | | 11.66 | | -0.88 | | 15.73 | | 15.73 | |
| Houston | -3.62 | | | 0.00 | | 8.04 | | 2.69 | | 9.19 | | 9.53 | |
| Los Angeles | -3.33 | | | 0.00 | | 9.97 | | -1.99 | | 13.06 | | 15.79 | |
| New York | -4.35 | | | 0.00 | | 15.83 | | -0.74 | | 21.67 | | 22.26 | |
| Seattle | -2.52 | | | 0.00 | | 8.68 | | -1.66 | | 11.02 | | 19.42 | |
| Percent Difference to American Indian or Alaska Native | | | | | | | | | | | | | |
| Chicago | -16.72 | | | -13.20 | | 0.00 | | -14.19 | | 4.61 | | 4.61 | |
| Houston | -12.68 | | | -8.74 | | 0.00 | | -5.81 | | 1.25 | | 1.62 | |
| Los Angeles | -14.77 | | | -11.07 | | 0.00 | | -13.28 | | 3.43 | | 6.47 | |
| New York | -23.97 | | | -18.80 | | 0.00 | | -19.68 | | 6.94 | | 7.65 | |
| Seattle | -12.26 | | | -9.50 | | 0.00 | | -11.32 | | 2.56 | | 11.77 | |
| Percent Difference to Asian | | | | | | | | | | | | | |
| Chicago | -2.22 | | | 0.87 | | 12.43 | | 0.00 | | 16.46 | | 16.46 | |
| Houston | -6.49 | | | -2.77 | | 5.50 | | 0.00 | | 6.68 | | 7.02 | |
| Los Angeles | -1.32 | | | 1.95 | | 11.72 | | 0.00 | | 14.75 | | 17.43 | |
| New York | -3.59 | | | 0.73 | | 16.44 | | 0.00 | | 22.24 | | 22.83 | |
| Seattle | -0.84 | | | 1.64 | | 10.17 | | 0.00 | | 12.47 | | 20.74 | |
| Percent Difference to Native Hawaiian/ Other Pacific Islander | | | | | | | | | | | | | |
| Chicago | -22.36 | | | -18.66 | | -4.83 | | -19.70 | | 0.00 | | 0.00 | |
| Houston | -14.11 | | | -10.12 | | -1.26 | | -7.15 | | 0.00 | | 0.37 | |
| Los Angeles | -18.85 | | | -15.02 | | -3.56 | | -17.31 | | 0.00 | | 3.14 | |
| New York | -33.22 | | | -27.66 | | -7.46 | | -28.60 | | 0.00 | | 0.76 | |
| Seattle | -15.22 | | | -12.38 | | -2.63 | | -14.25 | | 0.00 | | 9.45 | |
| Percent Difference to Hispanic or Latino | | | | | |  | |  | |  | |  | |
| Chicago | -22.36 | | | -18.66 | | -4.83 | | -19.70 | | 0.00 | | 0.00 | |
| Houston | -14.53 | | | -10.53 | | -1.64 | | -7.55 | | -0.37 | | 0.00 | |
| Los Angeles | -22.71 | | | -18.75 | | -6.91 | | -21.11 | | -3.24 | | 0.00 | |
| New York | -34.24 | | | -28.64 | | -8.28 | | -29.59 | | -0.77 | | 0.00 | |
| Seattle | -27.24 | | | -24.11 | | -13.34 | | -26.17 | | -10.43 | | 0.00 | |

| March 30^th^, 2020 - April 5^th^, 2020 | | | | | | |
| --- | --- | --- | --- | --- | --- | --- |
|  | White | Black or African American | American Indian or Alaska Native | Asian | Native Hawaiian/ Other Pacific Islander | Hispanic or Latino |
| Chicago | 0.78 | 0.86 | 0.97 | 0.82 | 1.01 | 0.88 |
| Houston | 0.77 | 0.84 | 0.99 | 0.86 | 1.01 | 0.87 |
| Los Angeles | 0.78 | 0.88 | 0.96 | 0.81 | 0.98 | 0.85 |
| New York | 0.93 | 1.04 | 0.98 | 0.97 | 0.96 | 0.83 |
| Seattle | 0.80 | 0.93 | 0.96 | 0.83 | 0.97 | 0.89 |
| Percent Difference to White | | | | | | |
| Chicago | 0.00 | -11.01 | -24.43 | -5.71 | -29.59 | -14.04 |
| Houston | 0.00 | -9.05 | -27.66 | -11.93 | -30.50 | -13.24 |
| Los Angeles | 0.00 | -11.60 | -21.90 | -3.88 | -25.07 | -7.82 |
| New York | 0.00 | -12.09 | -5.41 | -5.01 | -3.59 | 10.83 |
| Seattle | 0.00 | -16.57 | -20.65 | -4.84 | -21.62 | -11.32 |
| Percent Difference to Black or African American | | | | | | |
| Chicago | 9.92 | 0.00 | -12.09 | 4.78 | -16.74 | -2.73 |
| Houston | 8.30 | 0.00 | -17.06 | -2.64 | -19.66 | -3.84 |
| Los Angeles | 10.40 | 0.00 | -9.23 | 6.92 | -12.06 | 3.39 |
| New York | 10.79 | 0.00 | 5.96 | 6.32 | 7.58 | 20.45 |
| Seattle | 14.22 | 0.00 | -3.49 | 10.07 | -4.33 | 4.50 |
| Percent Difference to American Indian or Alaska Native | | | | | | |
| Chicago | 19.63 | 10.79 | 0.00 | 15.05 | -4.15 | 8.35 |
| Houston | 21.67 | 14.58 | 0.00 | 12.32 | -2.22 | 11.30 |
| Los Angeles | 17.97 | 8.45 | 0.00 | 14.78 | -2.59 | 11.56 |
| New York | 5.13 | -6.34 | 0.00 | 0.38 | 1.72 | 15.41 |
| Seattle | 17.11 | 3.38 | 0.00 | 13.11 | -0.80 | 7.73 |
| Percent Difference to Asian | | | | | | |
| Chicago | 5.40 | -5.01 | -17.71 | 0.00 | -22.59 | -7.88 |
| Houston | 10.66 | 2.57 | -14.05 | 0.00 | -16.59 | -1.16 |
| Los Angeles | 3.74 | -7.43 | -17.34 | 0.00 | -20.39 | -3.78 |
| New York | 4.77 | -6.75 | -0.38 | 0.00 | 1.35 | 15.08 |
| Seattle | 4.61 | -11.20 | -15.08 | 0.00 | -16.01 | -6.19 |
| Percent Difference to Native Hawaiian/ Other Pacific Islander | | | | | | |
| Chicago | 22.83 | 14.34 | 3.98 | 18.43 | 0.00 | 12.00 |
| Houston | 23.37 | 16.43 | 2.17 | 14.23 | 0.00 | 13.23 |
| Los Angeles | 20.04 | 10.77 | 2.53 | 16.94 | 0.00 | 13.79 |
| New York | 3.47 | -8.20 | -1.75 | -1.36 | 0.00 | 13.93 |
| Seattle | 17.78 | 4.15 | 0.80 | 13.80 | 0.00 | 8.47 |
| Percent Difference to Hispanic or Latino | | |  |  |  |  |
| Chicago | 12.31 | 2.66 | -9.11 | 7.30 | -13.64 | 0.00 |
| Houston | 11.69 | 3.69 | -12.74 | 1.15 | -15.24 | 0.00 |
| Los Angeles | 7.25 | -3.51 | -13.07 | 3.65 | -16.00 | 0.00 |
| New York | -12.15 | -25.71 | -18.21 | -17.76 | -16.18 | 0.00 |
| Seattle | 10.17 | -4.72 | -8.38 | 5.83 | -9.25 | 0.00 |

| April 6^th^, 2020 - April 12^th^, 2020 | | | | | | |
| --- | --- | --- | --- | --- | --- | --- |
|  | White | Black or African American | American Indian or Alaska Native | Asian | Native Hawaiian/ Other Pacific Islander | Hispanic or Latino |
| Chicago | 0.82 | 0.89 | 0.98 | 0.86 | 1.01 | 0.89 |
| Houston | 0.78 | 0.85 | 0.99 | 0.87 | 1.01 | 0.87 |
| Los Angeles | 0.76 | 0.86 | 0.95 | 0.80 | 0.98 | 0.83 |
| New York | 0.99 | 1.11 | 1.00 | 1.04 | 0.96 | 0.82 |
| Seattle | 0.86 | 0.97 | 0.99 | 0.90 | 0.99 | 0.89 |
| Percent Difference to White | | | | | | |
| Chicago | 0.00 | -9.34 | -19.36 | -5.09 | -23.03 | -8.43 |
| Houston | 0.00 | -8.47 | -25.81 | -11.02 | -28.35 | -11.09 |
| Los Angeles | 0.00 | -12.25 | -24.18 | -5.33 | -28.20 | -8.44 |
| New York | 0.00 | -12.02 | -0.55 | -5.33 | 2.69 | 16.89 |
| Seattle | 0.00 | -12.51 | -14.75 | -4.23 | -15.52 | -3.95 |
| Percent Difference to Black or African American | | | | | | |
| Chicago | 8.54 | 0.00 | -9.17 | 3.88 | -12.52 | 0.83 |
| Houston | 7.81 | 0.00 | -15.98 | -2.35 | -18.33 | -2.41 |
| Los Angeles | 10.92 | 0.00 | -10.62 | 6.17 | -14.21 | 3.40 |
| New York | 10.73 | 0.00 | 10.24 | 5.97 | 13.13 | 25.81 |
| Seattle | 11.12 | 0.00 | -1.99 | 7.36 | -2.68 | 7.61 |
| Percent Difference to American Indian or Alaska Native | | | | | | |
| Chicago | 16.22 | 8.40 | 0.00 | 11.96 | -3.07 | 9.16 |
| Houston | 20.51 | 13.78 | 0.00 | 11.75 | -2.03 | 11.70 |
| Los Angeles | 19.47 | 9.60 | 0.00 | 15.18 | -3.24 | 12.67 |
| New York | 0.55 | -11.41 | 0.00 | -4.76 | 3.22 | 17.34 |
| Seattle | 12.85 | 1.95 | 0.00 | 9.17 | -0.67 | 9.42 |
| Percent Difference to Asian | | | | | | |
| Chicago | 4.84 | -4.04 | -13.58 | 0.00 | -17.07 | -3.18 |
| Houston | 9.93 | 2.30 | -13.31 | 0.00 | -15.61 | -0.06 |
| Los Angeles | 5.06 | -6.58 | -17.90 | 0.00 | -21.72 | -2.96 |
| New York | 5.06 | -6.35 | 4.54 | 0.00 | 7.62 | 21.10 |
| Seattle | 4.06 | -7.94 | -10.09 | 0.00 | -10.83 | 0.27 |
| Percent Difference to Native Hawaiian/ Other Pacific Islander | | | | | | |
| Chicago | 18.72 | 11.13 | 2.98 | 14.58 | 0.00 | 11.87 |
| Houston | 22.09 | 15.49 | 1.98 | 13.50 | 0.00 | 13.45 |
| Los Angeles | 22.00 | 12.44 | 3.14 | 17.84 | 0.00 | 15.41 |
| New York | -2.76 | -15.11 | -3.33 | -8.24 | 0.00 | 14.59 |
| Seattle | 13.44 | 2.61 | 0.67 | 9.78 | 0.00 | 10.02 |
| Percent Difference to Hispanic or Latino | | | | | | |
| Chicago | 7.77 | -0.84 | -10.08 | 3.08 | -13.47 | 0.00 |
| Houston | 9.98 | 2.36 | -13.25 | 0.06 | -15.54 | 0.00 |
| Los Angeles | 7.78 | -3.52 | -14.51 | 2.87 | -18.22 | 0.00 |
| New York | -20.32 | -34.78 | -20.98 | -26.74 | -17.08 | 0.00 |
| Seattle | 3.80 | -8.24 | -10.40 | -0.28 | -11.14 | 0.00 |
